# Supplementary material for: Patients’ and physiotherapists’ perspectives on implementing a tailored stratified treatment approach for low back pain in Nigeria: a qualitative study
Source: BMJ Open. 2022 Jun 20;12(6):e059736. doi: 10.1136/bmjopen-2021-059736 (PMC9214370; doi:10.1136/bmjopen-2021-059736)
Supplement: Supplementary data [file bmjopen-2021-059736supp001.pdf]

Supplemental Table 1: Interview guideline for patients

Key Question	Maintenance question	Potential follow-up questions
1. Experiences (Warm-up): Tell me something about the LBP treatment you received so far.	- What else?<br>- And so on?<br>- Is there anything else	a) What is your expectation of a successful treatment for your LBP?<br>b) What are your concerns regarding the treatment of LBP?
2. Exploration: What is your opinion on the SB approach?	- What else?<br>- And so on?<br>- Is there anything else?	c) How would you feel being classified into a subgroup based on the SB tool ?<br>d) What would be your reaction if you were offered to be treated with this approach?<br>e) What can you say about the questions in the SB tool?
3. Factors: How does this approach compare with the current treatment you receive?	- What else?<br>- And so on?<br>- Is there anything else?	f) Mention any specific aspect of the SB approach which interests you?<br>g) What are your opinions regarding the tool process?<br>h) What do you think about communication with the therapist in this approach?<br>i) In the video, patients with a low likelihood of poor outcome receive one treatment session with examination and advice, how do you feel about this?<br>j) In the video, patients with a high likelihood of poor outcomes receive physical and psychological treatment, how do you feel about this?<br>k) In your opinion, what do you expect from physiotherapists who use this approach e.g. attitude, training, expertise?<br>l) What is your opinion on a patient's responsibility regarding this approach?<br>m) In your opinion, tell me what is required to use this approach in Nigeria?<br>n) Tell me if other enablers or barriers come to your mind when you think of this approach?
4. Related influences: What do you think might happen when this approach is used?	- What else?<br>- And so on?<br>- Is there anything else	o) How do you think this approach affects you as a patient with LBP?<br>p) How does this approach affect how you view treatment for LBP in Nigeria?

LBP: Low back pain; SB: STarT-Back
