# Supplementary material for: Patients’ and physiotherapists’ perspectives on implementing a tailored stratified treatment approach for low back pain in Nigeria: a qualitative study
Source: BMJ Open. 2022 Jun 20;12(6):e059736. doi: 10.1136/bmjopen-2021-059736 (PMC9214370; doi:10.1136/bmjopen-2021-059736)
Supplement: Supplementary data [file bmjopen-2021-059736supp002.pdf]

Supplemental Table 2: Interview guideline for physiotherapists

Key Question	Maintenance question	Potential follow-up questions
1. Exploration: What is your perception of the SB approach?	• What else?<br>• And so on?<br>• Is there anything else?	a) Tell me something about your current approach to treatment for non-specific LBP.<br>b) How do you consider the idea of classifying patients into subgroups based on the SB tool?<br>c) How would you feel about allocating treatments in this treatment approach?
2. Factors: How does this approach compare with the current treatment for non-specific LBP in Nigeria?	• What else?<br>• And so on?<br>• Is there anything else?	d) Mention any specific attributes of the SB approach that might interest you.<br>e) What can you say about the SB tool?<br>f) What is your opinion about the patient-centeredness of this approach?
3. Strategies: Tell me any possible requirements for implementing this approach in Nigeria?	• What else?<br>• And so on?<br>• Is there anything else?	g) What are your ideas on the handling of the tool?<br>h) In your opinion, what is required for the implementation of the High-risk approach?<br>i) In your opinion, what is required for the implementation of the Low-risk approach?<br>j) What are your ideas about incentives for using this approach?<br>k) Tell me your ideas about training on this approach.<br>l) What comes to your mind when you think about the distribution of this approach in Nigeria?<br>m) Tell me if other enablers and barriers come to your mind when you think of this approach?
4. Related influences: What effects do you think might result from its use?	• What else?<br>• And so on?<br>• Is there anything else?	n) How might this approach affect patients receiving physiotherapy in Nigeria?<br>o) How might it affect interprofessional relations in Nigeria?

LBP: Low back pain; SB: STarT-Back
