# Supplementary material for: Patients’ and physiotherapists’ perspectives on implementing a tailored stratified treatment approach for low back pain in Nigeria: a qualitative study
Source: BMJ Open. 2022 Jun 20;12(6):e059736. doi: 10.1136/bmjopen-2021-059736 (PMC9214370; doi:10.1136/bmjopen-2021-059736)
Supplement: Supplementary data [file bmjopen-2021-059736supp003.pdf]

Supplemental Table 3: Coding agenda with category descriptions

**Category 1: Resistance to change.**<br><br>**Themes of variation**	**Category Description: A mixed category because it describes views from patients and physiotherapists. It describes the resistance perceived and experienced by physiotherapists and patients concerning introducing a stratified care approach. It describes the physiotherapists and patients' perceptions of challenges to the paradigm shift and strategies to overcome resistance.**		
	**Resistance to change**	**Explanation**	**Quotes***
**Tradition of treatment**<br>This describes components of the usual care, highlighting gaps in practice and aspects that can be modified to optimise care.	Incentives attached to the usual practice	Physiotherapists tradition of treatment was a major challenge, it seemed to be an overwhelming issue sustained by Incentives attached to the usual practice. Key aspects are ego, self-confidence in their current practice experience and finance. There is a long-held tradition of treatment with non-evidence-based methods, diagnosis and radiographs that the patient has gotten used to and this tradition remains deeply entrenched in practice.	***PTf7:*** *I would say it has a lot to do with ego, they won't want you to tell them what to do... If I go for training and come back with a new trend, I would start using it immediately, but your colleagues, now avoiding it because it comes from your colleague.*<br><br>***PTf7**: Physiotherapists usually shy away when there is a new protocol, implementing it will just be for record purposes, they think it will stress them and is time-wasting.*
**Evolution to a new system**<br>This discusses the key enablers needed to introduce a new practice aimed at optimising care.	Overcoming patients' expectations	Therapists see patient expectation *as* a primary deterrent to the implementation of stratified care that needs to be addressed. It feels to them like all their suggestions and efforts to optimising practice will not work because the patients come with a certain expectation. They expect a strong touch, massage or something physical that would bring the pain down to zero. There is, however, an enabling situation seen by therapists and patients, that these expectations can be adjusted and overcome.	***PatF1:*** *I wish it can go, I wish my pain can go… I think it will need some kind of strong force for it to go, maybe I should like a fall or something to put it in position.*<br><br>***PTm5:*** *I think it's just marrying the expectations of the patient with what the physiotherapist thinks is realistic, and with that, there could be some kind of trade-off.*
**Experiences**<br>This describes instances where attempts to install change have been made and the effects	Organisational culture	From the experiences of physiotherapists and patients, the organisational culture preponderant in Nigeria makes treatment less efficient and less effective. They have limitations in their ability to ease the changes beyond a certain point like organising situations, follow-ups or influence patient's working conditions.	***PatM2:*** *If you tell me to modify my workstation and I know that I cannot do it you are wasting your time. but if you tell me the office seat needs to be changed and in one week someone brings the right one I will have the right attitude.*<br><br>***PTm9**: There might be challenges, the kind of environment and mentality here in Nigeria is different.*
**Strategies for implementation**<br>This highlights the participants' key ideas on strategies for the implementation of the stratified care approach.	Targeting attitudinal change	A change in the attitude of physiotherapists and patients is seen as a major strategy to improve the chances of successful implementation. The physiotherapist and patients see themselves as mostly complacent. The patients blamed the physiotherapists for not being attentive to their needs and the physiotherapists blamed the patient's attitude as a deterrent for being unable to improve their condition.	***PatM8:*** *The period I was there I don't think there was a lot of Patient load, we were not more than ten patients attending physiotherapy so I think it is their attitude that needs to change.*<br><br>***PTf8:*** *Well, I have tried to push it, and it didn't work…some patients have an attitude like you are trying to test their knowledge…so somehow this attitude has affected the use by physiotherapists.*

* Physiotherapists' and patient's quotes with pseudonymous designations comprising the numerical order of interviews.

**Category 2: Acceptance of innovation**<br><br>**Themes of variation**	**Category description: This is a category most relevant to physiotherapists. It describes a positive reaction from physiotherapists to the idea of implementation of stratified care in Nigeria, highlighting their perspectives on the best settings and enabling conditions for implementation with inputs from patients.**		
	**Acceptance of innovation**	**Explanation**	**Quotes***
**Tradition of treatment**<br>This describes components of the usual care, highlighting gaps in practice and aspects that can be modified to optimise care.	Ease of transition	Some physiotherapists reported similarity of stratified care to what they already know (tradition of treatment) and practice in their management of LBP, they have background knowledge of the biopsychosocial approach and vary treatment for patients. These are individual aspects of stratified care and participants reported that this makes it easy to fully adopt the stratified care in its entirety.	***PTf7:*** *You are just giving it a name, it is comparable to what we do already here…. well, now all patients are treated more individually, we do not use the same intervention for every LBP patient anymore we have improved.*<br><br>***PTf11:*** *Yes, just as I said it's not really different from what we do here, …we do not have a name for it but it is essentially the same as what this approach is about, but I can speak for my centre only. because I will say my centre is about the best in Nigeria in terms of the content of practice so far.*
**Evolution to a new system**<br>This discusses the key enablers needed to introduce a new practice aimed at optimising care.	Open to new knowledge	Physiotherapists realising this approach is fundamentally new, uncommon and not used in its entity in practice, yet they were open to new knowledge describing stratified care as an improvement to current care and welcome development.	***PTm1****: So far, I think it is a welcome development because the approach is nice because it tends to categorise individuals with LBP in two categories people that have low risk moderate and high categorising individuals into group will give it a definition into the line of management or better outcome.*<br><br>***PTf4:*** *My perception in summary about the approach is that it is a good one and that it would help physiotherapist a lot.*
**Experiences**<br>This describes instances where attempts to install change have been made and the effects	Steps towards optimising practice	From their experiences, participants came across situations where they made steps towards optimising practice, instances where innovations, ideas or approaches have been introduced in their various work settings, the process that helped and the positive responses from colleagues. Key aspects were the use of clinical discussions, through physiotherapists cadre and time-saving measures for the patient. They think the stratified care being introduced in a similar stepwise manner will be successful.	***PTf7:*** *Since everyone is on board, especially our head of department, he likes such things, if he sees the evidence he would say 'from today we are using this approach and it must be used and expedite the use.*<br><br>***PTm9:*** *(In cases of innovation in my clinical setting) the same strategy, we discuss it at seminars and everyone brings their opinions and decides to use it and see the result. only then can it become a norm.*
**Strategies for implementation**<br>This highlights the participants' key ideas on strategies for the implementation of the stratified care approach.	Work Settings	There were differing perspectives on the most viable work setting for targeting the implementation of stratified care since there exist different situations and ideologies at the private and government establishments in Nigeria. Participants felt it would be more applicable if targeted at the government hospitals since the therapists are paid by the government a fixed amount and more treatments do not lead to more money.	***PTf12:*** *To be honest the government hospitals make it easier to use these approaches and questionnaires. Because most of the private settings didn't have these things available, so most times I have to source for them to use, but in the government hospitals they were printed and ready for use.*<br><br>***PTm3:*** *Especially in the private practice where you need more patients to have more income, so the more the patients the more the income, so that is the case, it means the physiotherapist might not want to use that approach but, in the government-owned hospitals where that might not be the case, they may be more willing to go in that direction.*

LBP: Low back pain
* Physiotherapists' quotes with pseudonymous designations comprising the numerical order of interviews.

**Category 3: Adapting practice**<br><br>**Themes of variation**	**Category Description: This category is relevant to patients and physiotherapists. Here participants highlight their opinions and experiences on ways to adopt the stratified care approach. This category describes the suggestions on adaptations in terms of creating awareness and modification of the approach to suit the Nigerian context and enhance its use.**		
	**Adapting practice**	**Explanation**	**Quotes***
**Tradition of treatment**<br>This describes components of the usual care, highlighting gaps in practice and aspects that can be modified to optimise care.	Need of standard for regulating the practice	Participants highlighted the absence of a standard of practice tailored to the needs of Nigerians. This situation led to a clamouring for standard to guide practice and regulation. Adherence to such a standard of practice would enhance adaptation for implementation.	***PTf7:*** *In Nigeria, we do not have a standard way of doing things. what hospital A is doing is different from what Hospital B is doing… everyone is just doing what they want.*<br><br>***PatM8:*** *With proper supervision, if the hospital management focuses on what happens in the hospital I am sure there will be room for improvement, for both the staff and the facility.*
**Evolution to a new system**<br>This discusses the key enablers needed to introduce a new practice aimed at optimising care.	Cultural adaptations	Socio-cultural adaptations relating to language, the culture of respect and hierarchical implementation from the seniors to juniors was highlighted as a viable means of adapting practice at the introductory phases.	***PatF5****: The clinician speaks our language…reads to her…or when she comes home the children do.*<br><br>***PTm2:*** *When the chiefs and assistant directors in each unit champion this, then junior physiotherapists will use it.*
**Experiences**<br>This describes instances where attempts to install change have been made and the effects	Use of communication	Communication was seen as a major way to inform adaptation and improve the patient-therapist relationship. When communication between patient and physiotherapist was used it was seen to produce positive effects.	***PTf8****: It is feasible, I always use conversations to help my Patients see the benefits.*<br><br>***PatM2:*** *Well it is communication; a patient needs to know why they are allocated to a particular group.*
**Strategies for implementation**<br>This highlights the participants' key ideas on strategies for the implementation of the stratified care approach.	Awareness for patients and physiotherapists	Participants saw creating awareness about the approach as a major strategy for implementation. While some opined this can be done at undergraduate and post-graduate levels for physiotherapists, patient's awareness was achievable through outreaches.	***PatF9****: And awareness…when I went for their free medical seminar I was able to meet the physiotherapist guy that came for outreach, … which encouraged me.*<br><br>***PTF9:*** *Also, there should be health promotion done to create awareness about it.*

* Physiotherapists' and patient's quotes with pseudonymous designations comprising the numerical order of interviews.

**Category 4: Patient's Learning Journey Themes of variation**	**Category Description: This category is relevant to patients and physiotherapists. Here, patients have experienced care from various fronts and have formed their opinions about what they have experienced to be helpful to their condition. This was corroborated by the physiotherapist opinions about the patients learning experiences from their perspective.**		
	**Patient's Learning Journey**	**Explanation**	**Quotes***
**Tradition of treatment** This describes components of the usual care, highlighting gaps in practice and aspects that can be modified to optimise care.	Needing a complement to usual care	For patients, the usual care was seen to be insufficient and there are aspects that patients feel would need some form of compliment, they feel this can be achieved by treatment from traditional bone setters care which is seen as a compliment or an alternative.	***PTm5****: Most people prefer to go to bonesetters and the likes because the people around them would have told them that this is what someone used and the person got better so.*<br><br>***PatM2:*** *There is a lot in it (traditional bone setters care) that complements orthodox medicine'…I can manage my pain better.*
**Evolution to a new system** This discusses the key enablers needed to introduce a new practice aimed at optimising care.	Recognising unhelpful treatments	Participants report that over the past recent years, patients have gradually become mentally equipped to recognise unhelpful treatments by themselves and developing the ability to differentiate between what is beneficial and what isn't. This realisation is seen as a viable enabler to the implementation of stratified care.	***PatM2:*** *The former treatment was more like going thru a particular regimen and go back to the same condition that brought the pain but the approach …suggests lifestyle changes which if sustained gives permanent relief.*<br><br>***PatM3:*** *Well, to me I tried it and it made matters worse, so I wouldn't advise anyone to do that, to me that is a no-go area because they are working blindly.*
**Experiences** This describes instances where attempts to install change have been made and the effects.	Learning to live with pain	Participants explain how the debilitating condition of the patient's experiences have resulted in personal difficulties. They resolved in their minds to change their outlook and accept the condition and this has improved their outlook.	***PatF9****: I feel depressed most times, it affects me so much, … I think it is what I would work on now, psychologically I know it is affecting me because I am thinking so much about it and it is giving me so much concern.*<br><br>***PatM3:*** *It affected me psychologically, I was a worrying bit I have learned to live with it, I think I am better at managing myself …so also, I have learned, I have concluded that no kind of intrusive treatment can work for this my case, that I just need to be careful with how I live my life.*
**Strategies for implementation** This highlights the participants' key ideas on strategies for the implementation of the stratified care approach.	Taking charge	Patients have gathered knowledge of self from various treatment exposures, from self-care, traditional bone setters' treatments. They have now decided that taking charge of their treatment is a vital strategy to help themselves.	***PatF7:*** *Yes, what I can generally say is it helped me to know how to better manage the back by myself.*<br><br>***PatF9:*** *I would love to do it (treatment) myself…rather than going to the hospital.*

* Physiotherapists' and patient's quotes with pseudonymous designations comprising the numerical order of interviews.

**Category 5: Trusting the physiotherapist**<br><br>**Themes of variation**	**Category Description: This category is most relevant to patients. It describes the patient's views of the physiotherapist as a dynamic expert with who they need to give their trust and co-operation based on the efforts of the physiotherapist to help their situation and their experiences with treatments.**		
	**Trusting the physiotherapist**	**Explanation**	**Quotes***
**Tradition of treatment**<br>This describes components of the usual care, highlighting gaps in practice and aspects that can be modified to optimise care.	Getting some help	The aspects of the usual care focused on exercise and advice was seen to provide some relief to patients, even though not permanent they relate some improvement in their condition and trust that based on this result more can be expected.	***PatF7****: The therapy I underwent helped though because before I started the treatment I could not even lift myself from the bed …but the first week of the therapy I would say I experienced relief.*<br><br>***PatM3****: I started getting relief because my clinicians were trained, even the advice I received worked to some extent. So, I believe that clinicians treating patients need the training to further update their treatment methods.*
**Evolution to a new system**<br>This discusses the key enablers needed to introduce a new practice aimed at optimising care.	Learning with practice	The patients feel that the physiotherapists will gradually evolve in their practice and that some therapists are already on the right track, learning to change their approach with practice. They feel exploring ideas like this through research is needed to evolve more.	***PatM3:*** *What you are doing is good, research is the main factor so just keep it up. there is no way around it, to give patients the best available treatment there is only one way to make sure of it and that is research. continue to make research that's the future of the medical practice.*<br><br>***PatM4****: It is possible they know these things already, because you know they are experts and they have been dealing with people having the same issues with me for a long time, so they can juxtapose and see if that is the problem or a factor.*
**Experiences**<br>This describes instances where attempts to install change have been made and the effects	Therapists doing their best	The patients acknowledge that the physiotherapists are putting in efforts to optimise care in ways they can and they appreciate the efforts, they notice when some more is done to help their condition and that encourages them to focus more on the efforts.	***PatF9:*** *That therapist tried, he did a lot, I told him I appreciated his work very well…. they tried a lot, I am satisfied with their level of experience.*<br><br>***PatM8****: He talked about some exercise but he advised me majorly about my sleeping position, which has helped so much.*
**Strategies for implementation**<br>This highlights the participants' key ideas on strategies for the implementation of the stratified care approach.	Co-operating with the therapists	The patients consider the physiotherapist's role as providing care and support for the patient when needed and their role as patients is not to interfere with the process. They feel they need to co-operate with the physiotherapist but largely leaving the aspect of care for the therapists.	***PatM5****: For me, I believe that their field and they are the specialists. I can't say, I try as much as possible not to go into areas where I have low competence.*<br><br>***PatM3****: You know when the pain becomes unbearable, you need to consult a specialist, at least to know what is happening and have their support.*

* Patient's quotes with pseudonymous designations comprising the numerical order of interviews.

**Category 6: Needing conviction**<br><br>**Themes of variation**	**Category Description: This category is most relevant to patients. It describes patients exposure to false information sources, hence the need for physiotherapists to provide trustworthy education to get their full co-operation. This equips patients on their road to self-discovery and guides them to conviction.**		
	**Needing conviction**	**Explanation**	**Quotes***
**Tradition of treatment**<br>This describes components of the usual care, highlighting gaps in practice and aspects that can be modified to optimise care.	Reliance on investigations	Physiotherapists are already used to a diagnosis-based approach, using radiographs and passive treatments. Since the patients have become used to that, they need some form of conviction to accept the change. Some patients enjoy passive treatments and would not only prefer that but insist it is the right thing to do.	***PTf10****: There is the likelihood that patients will not accept this, they will go after the treatment to another therapist…some tell you how much they enjoy the sessions…they keep coming back to enjoy the sessions.*<br><br>***PatM8****: Investigations are not done. I had to muscle my way to do an MRI by myself, nobody told me about that I just knew it was necessary and I decided to do that.*
**Evolution to a new system**<br>This discusses the key enablers needed to introduce a new practice aimed at optimising care.	Patient education	For patients to be able to accept change, they need to be informed properly to a level of conviction. Patients have multiple sources of information and this needs to be presented clearly and reasonably to influence attitudes and lifestyle. If patients can be educated and the procedures explained properly, it will go a long way to influence their expectations and help them become more receptive to a change in approach.	***PTm9****: It is possible they might also accept the treatment if it costs less but they need to be educated, in such a situation one has to spend more than that ten minutes to convince them.*<br><br>***PatM2****: You see, the patient attitude is determined by the amount of information that he has, so if you can clearly explain the approach and how it works you are likely to get a positive attitude from the patient.*
**Experiences**<br>This describes instances where attempts to install change have been made and the effects	Self-discovery	Patients relate situations when they needed to be convinced from within, even though their therapist tried by advising them they still needed to see for themselves to be successfully convinced to overcome the initial resistance. Based on their experiences, they relate that their outlook changed and they experience better outcomes.	***PatF7****: The clinician asked me the kind of bed I used… I told him it cannot be my bed, and all that but you know after the whole thing I was querying things, then I travelled for two weeks and I used another bed then I was able to conclude it was the bed.*<br><br>***PatF9****: I do exercise often but I stopped, I fear that what I am doing is even increasing the pain. I don't want to worsen the condition, and my husband told me to stop… but when I went to the clinic they did something similar and it subsided.*
**Strategies for implementation**<br>This highlights the participants' key ideas on strategies for the implementation of the stratified care approach.	Struggle against false information	Patients relate that there is a variety of information sources and many of which are misleading. These can come from other patients, from the internet or clinicians with certain motives. There is a constant struggle against wrong information by the patients which the therapists need to drive against to optimise care.	***PatM3****: Yes, they do mislead patients sometimes for financial implications, they may end up telling you to do something that is not necessary they can advise the patient to go for that treatment even though they know it might not help, so it requires a medical practitioner with integrity to be able to come out truthfully.*<br><br>***PatM2****: Yes, so be careful of the advice you get from clinicians because you might be getting it from quacks, make sure you get the advice from the right source.*

* Physiotherapists' and patient's quotes with pseudonymous designations comprising the numerical order of interviews.

**Category 7: Recognising the need for change**<br><br>**Themes of variation**	**Category Description: This category is relevant for physiotherapists and patients. It describes participants attention to deficiencies and deterrents in physiotherapists knowledge and practice, further corroborated by the patient's dissatisfaction with the current treatment outcomes which can be enabled by acceptance of the stratified care approach and proper funding.**		
	**Recognising the need for change**	**Explanation**	**Quotes***
**Tradition of treatment**<br>This describes components of the usual care, highlighting gaps in practice and aspects that can be modified to optimise care.	Lack of training to give psychologically informed therapy	Physiotherapists admitted having gaps in knowledge and lapses in training that might affect their ability to deliver optimal biopsychosocial care, this has resulted in sticking to the more familiar tradition of treatment. This issue of competency was a major setback. Physiotherapists felt incompetent to handle such an approach especially the prognostic approach and psychologically informed therapy.	***PTf4****: Most therapists do know about the prognosis of LBP condition, they need to be educated better on prognosis because you can't classify patients using this approach if you do not know'*<br><br>***PTf8****: Although general psychology was taught in school, this one relating to health care isn't taught. they do not know what exactly to do and how to do it. we just use the common-sense approach.*
**Evolution to a new system**<br>This discusses the key enablers needed to introduce a new practice aimed at optimising care.	Embracing a different approach	Physiotherapists and patients need to recognise the potential to optimise care to be open to accepting a different approach. The potential advantages need to be clear to participants creating an undeniable appeal of a different approach.	**PTm6**: *A model like this … I feel if we embrace it one way or the other it would make a difference.*<br><br>***PTf4****: I think it will make life easier for patient and Physiotherapist if they can adopt it will go a long way to help,*
**Experiences**<br>This describes instances where attempts to install change have been made and the effects	No complete relief	Patients have undergone a series of treatments to change their condition but with no complete relieved some now seek solutions elsewhere. They express disappointment with their situation and frustration with the care they receive. They all feel that things need to change in the way physiotherapists treat patients.	***PatM4****: Drugs, physiotherapy, I am not completely relieved, I just got tired of going to the hospital. because I have been going for physiotherapy for the past 2-3 years*<br><br>***PatM8****: I thought by now I should have been fine…I feel the physiotherapy and medication wasn't doing anything to help my situation…I just felt I wasted my money and time,*
**Strategies for implementation**<br>This highlights the participants' key ideas on strategies for the implementation of the stratified care approach.	The role of funding	Participants complain of general issues relating to infrastructure, equipment and patient load. These issues are systemic constraints that are neither the fault of the patients nor the therapists. Participants jointly suggest improved funding to the health sector will improve services and aid implementation.	***PTm6****: Facility and equipment are some of the main issues. No physiotherapist would be willing to use his funds to go print questionnaires for patients, most of our offices do not have computers, and printers available.*<br><br>***PatM8****: Very poor, the infrared light they use on me everyone else has to wait until they are done with me, they have two and one is bad. So, if I needed it for ten mins it might be cut down to two or three minutes so that others can also use it.*

LBP: Low back pain

* Physiotherapists' and patient's quotes with pseudonymous designations comprising the numerical order of interviews.
