# Supplementary material for: Patients’ and physiotherapists’ perspectives on implementing a tailored stratified treatment approach for low back pain in Nigeria: a qualitative study
Source: BMJ Open. 2022 Jun 20;12(6):e059736. doi: 10.1136/bmjopen-2021-059736 (PMC9214370; doi:10.1136/bmjopen-2021-059736)
Supplement: Author's manuscript [file bmjopen-2021-059736.draft_revisions.pdf]

BMJ Open is committed to open peer review. As part of this commitment we make the peer review history of every article we publish publicly available.

When an article is published we post the peer reviewers' comments and the authors' responses online. We also post the versions of the paper that were used during peer review. These are the versions that the peer review comments apply to.

The versions of the paper that follow are the versions that were submitted during the peer review process. They are not the versions of record or the final published versions. They should not be cited or distributed as the published version of this manuscript.

BMJ Open is an open access journal and the full, final, typeset and author-corrected version of record of the manuscript is available on our site with no access controls, subscription charges or pay-per-view fees (http://bmjopen.bmj.com).

If you have any questions on BMJ Open's open peer review process please email info.bmjopen@bmj.com

BMJ Open

Patients' and physiotherapists' perspectives of implementing a tailored stratified treatment approach for low back pain in Nigeria: a qualitative study.

Journal:	*BMJ Open*
Manuscript ID	bmjopen-2021-059736
Article Type:	Original research
Date Submitted by the Author:	30-Nov-2021
Complete List of Authors:	Adje, Mishael; Trier University of Applied Sciences; Institute of Family Medicine, University of Lübeck, Germany<br>Steinhäuser, Jost; Institute of Family Medicine, University of Lübeck, Germany<br>Stevenson, Kay; The Medical School Keele University<br>Mbada, Chidozie Emmanuel; Obafemi Awolowo University, Department of Medical Rehabilitation<br>Karstens, Sven; Trier University of Applied Sciences, Therapeutic Sciences, Department of Computer Science
Keywords:	QUALITATIVE RESEARCH, Back pain < ORTHOPAEDIC & TRAUMA SURGERY, HEALTH SERVICES ADMINISTRATION & MANAGEMENT, Musculoskeletal disorders < ORTHOPAEDIC & TRAUMA SURGERY, PAIN MANAGEMENT

SCHOLARONE™
Manuscripts

I, the Submitting Author has the right to grant and does grant on behalf of all authors of the Work (as defined in the below author licence), an exclusive licence and/or a non-exclusive licence for contributions from authors who are: i) UK Crown employees; ii) where BMJ has agreed a CC-BY licence shall apply, and/or iii) in accordance with the terms applicable for US Federal Government officers or employees acting as part of their official duties; on a worldwide, perpetual, irrevocable, royalty-free basis to BMJ Publishing Group Ltd ("BMJ") its licensees and where the relevant Journal is co-owned by BMJ to the co-owners of the Journal, to publish the Work in this journal and any other BMJ products and to exploit all rights, as set out in our licence.

The Submitting Author accepts and understands that any supply made under these terms is made by BMJ to the Submitting Author unless you are acting as an employee on behalf of your employer or a postgraduate student of an affiliated institution which is paying any applicable article publishing charge ("APC") for Open Access articles. Where the Submitting Author wishes to make the Work available on an Open Access basis (and intends to pay the relevant APC), the terms of reuse of such Open Access shall be governed by a Creative Commons licence – details of these licences and which Creative Commons licence will apply to this Work are set out in our licence referred to above.

Other than as permitted in any relevant BMJ Author's Self Archiving Policies, I confirm this Work has not been accepted for publication elsewhere, is not being considered for publication elsewhere and does not duplicate material already published. I confirm all authors consent to publication of this Work and authorise the granting of this licence.

For peer review only

Patients' and physiotherapists' perspectives of implementing a tailored stratified treatment approach for low back pain in Nigeria: a qualitative study.

Adje Mishael[1,2], Jost Steinhäuser[2], Kay Stevenson[3], Chidozie Mbada[4], Sven Karstens[5]

[1]Trier University of Applied Sciences, Trier, Germany. [2] Institute of Family Medicine, University of Luebeck, Germany, [3]The Impact Accelerator Unit, The Medical School Keele University, Keele, UK, [4] Department of Medical Rehabilitation, Obafemi Awolowo University, Ile-Ife, Nigeria, [5]Therapeutic Sciences, Department of Computer Science, Trier University of Applied Sciences, Trier, Germany.

Correspondence to Mishael Adje; adjem@hochschule-trier.de.

Abstract

Background: Stratified care has the potential to be efficient in addressing the physical and psychosocial components of low back pain (LBP) and optimise treatment outcomes essential in low-income countries. This study was aimed to investigate the perceptions of physiotherapists and patients in Nigeria towards stratified care for the treatment of LBP, exploring barriers and enablers to implementation.

Methods: A qualitative design with semi-structured individual telephone interviews for physiotherapists and patients with LBP comprising research evidence and information on stratified care was adopted. Preceding the interviews, patients completed the STarT-Back Tool. The interviews were recorded, transcribed and analysed following grounded theory methodology.

Results: Twelve physiotherapists and thirteen patients with LBP participated in the study (11 female, mean age 42.8 (SD 11.47) years). Seven key categories emerged: *recognising the need for change, acceptance of innovation, resistance to change, adapting practice, patients learning journey, trusting the therapist and needing conviction.* Physiotherapists perceived stratified care to be a familiar approach based on their background training. The prevalent treatment tradition and the patient expectations were seen as major barriers to implementation of stratified care by the physiotherapists. Patients see themselves as more informed than therapists realise, yet they need conviction through communication and education to co-operate with their therapist using this approach. Viable facilitators were also identified as patients trust in the physiotherapist and adaptations in terms of training and modification of the approach to enhance its use.

Conclusion: Key barriers identified are the patient's treatment expectations and physiotherapists' adherence to the tradition of practice. Physiotherapists might facilitate implementation of the stratified care by communication, hierarchical implementation and utilisation of patients' trust. Possibilities to develop a consensus on key strategies to overcome barriers and on utilization of facilitators should be tested in future research.

Keywords: Qualitative research, Low back pain, Physiotherapy, Grounded theory, Stratified Care

For peer review only

Strengths and limitations of this study

- The barriers and enablers to implementation of stratified care in low income settings like Nigeria is explored from the perspective of patients and physiotherapists in this study.
- This study adhered to a rigorous circular grounded theory method to systematically draw out comparisons and variations in perspectives of 25 participants until saturation was reached.
- Participants were motivated to engage in productive discussions and involved in co-designing introductory materials and tailoring interview guides.
- The need for stakeholder awareness in areas of divergent perspectives, to inform quality communication and patient conviction seen in this study is in concordance with current evidence on stratified care implementation in other contexts.
- Tailored strategies should be developed in a structured manner in order to handle identified barriers and enablers.

For peer review only

Introduction

Low back pain (LBP) is among the most frequent reasons for visits to outpatient physiotherapy clinics in Nigeria (1–3). It is a prominent cause of disability and the effects have been immense in terms of cost, time and productivity loss (4–7).

In recent decades, there has been remodelling in the management of LBP. Current literature corroborated by multiple guidelines recommend the biopsychosocial model of care (8–13). These resources highlight the prognostic significance of psychosocial factors such as depression, fear-avoidance behaviours, catastrophising, distress among many in individual patients and their culpability in worsening musculoskeletal conditions (14, 13).

Stratified care encompasses specific concepts in the management of LBP, differentiating and targeting prognostic subgroups, aligning the risk of an unfavourable treatment outcome with specific evidence-based treatment procedures (15). Its principles lean towards prognosis, de-emphasising pain, disputing unhelpful beliefs and equipping patients on self-management strategies (13, 8, 9). It aims to guide clinicians to identify psychosocial risk factors hindering patient recovery and fast track accurate treatment decisions (16, 17).

One well-known stratification approach with demonstrated effectiveness in both clinical and cost outcomes is the Subgroups for Targeted Treatment (STarT-Back) approach (18, 19). It involves categorising LBP patients into low, medium and high risk categories using the STarT-Back tool (SBT) and allocating matched treatments to participants in each category (20).

The SBT was developed for primary health care settings, it is a validated tool that can be administered paper-based or online-based. It consists of 9-items, designed to assess modifiable risk factors (20). The first four questions address aspects of the patient's physical characteristics asking about the spread of the pain towards the leg, to the neck or shoulders, and disability in patients' dressing or walking. The next five questions comprise the psychosocial subscale relating to patients' psychological characteristics, including fear of movement, anxiety, catastrophising, depression and bothersomeness (20).

In a large scale study evaluating the effectiveness of the STarT-Back approach, the score from the SBT was used to classify patients into three subgroups (19). Depending on the subgroup, there are treatment recommendations. If a patient scores between 0 and 3 in total on the SBT, they are allocated to the low-risk subgroup. These comprise patients who will improve with little or no intervention. They are helped with advice and pain medication. If patients score between 4 and 9 on the grouping tool but have 3 or fewer of the five sub-scale, they are allocated to the medium-risk subgroup. These have a moderate likelihood of poor treatment outcomes, they have lesser physical and psychological components of pain. The emphasis here is on physiotherapy and self-management (21). Patients who score 4 of 5 on the psychological subscale are allocated to the high-risk subgroup. These patients possess complex

For peer review only

prognostic factors impeding recovery and need more care due to these. Such care comprises psychologically informed physiotherapy, aimed to resolve physical and psychosocial components of pain (15, 19). All target treatments are aimed to reduce disability, reduce pain where possible, improve psychological functioning and enable the patient to manage ongoing and/or future episodes of back pain (21).

Studies from the UK and New Zealand reveal that the implementation of the STarT-Back model of care has the potential to reduce disability, save costs, improve patient satisfaction, reduce the number of patient visits and increase clinician's competence (19, 22, 23) except for a US study indicating no effect on patient outcomes (24). A recent systematic review shows that this approach provides benefits in terms of clinical outcomes and health-related cost savings for all three subgroups of patients and patients in the high-risk subgroup who received psychologically informed treatment had significant improvements in pain, disability, depression and general health (25). The SB approach is described as best practice in the UK international guideline for the management of LBP (26, 11) and has been recommended from studies in the UK and other countries to be efficacious and have a positive impact on LBP outcomes (18, 21, 19). However, these are mostly high-income countries (UK, Denmark) with financially sound health care systems, further care is taken to ensure proper implementation in countries in Africa

Studies on implementation efforts in higher-income countries exhibit challenges further amplified when considering contextual factors present in lower/middle-income countries (18, 19, 15). A study shows, that socio-economic status is an important treatment effect modifier when using the SB approach (27). Differences in settings can also play a major role in determining the outcome of implementation (24). Unique circumstances make it challenging for some physiotherapists to consider practice guideline recommendations when planning treatment (28). This leads to the omission of psychosocial risk factors, which undermines treatment outcome (29) and is present in over 60% of patients with chronic LBP in Nigeria (30). In combating this, implementation of the STarT-Back model of care promises a viable solution. Since there is no universal instruction manual for implementation, it is recommended that stratified care for LBP will first need adaptation in terms of setting before wider implementation is considered. The first step to this is identifying the contextual barriers and enablers to implementation (15).

This study was aimed to explore the perspectives of physiotherapists and patients with LBP, identifying perceived barriers and enablers to the implementation of stratified care in Nigeria.

Methods

A qualitative design was adopted in this study. Semi-structured individual telephone interviews for patients and physiotherapists were extensively executed following grounded theory methodology (31,

For peer review only

32). The Consolidated Criteria for Reporting Qualitative Research (COREQ) was employed for the study report (33).

Interviewer/Facilitator

The research team was made up of five researchers: MA (male), a Nigerian physiotherapist with experience in musculoskeletal health; SK (male), a physiotherapist with experience in qualitative and health services research; JS (male), a general practitioner with experience in medicine and qualitative research; KS (female), a physiotherapist with experience in health promotion and implementation of the STarT-Back approach; and CM (male) a physiotherapist with experience in musculoskeletal research in Nigeria. The interview guidelines were prepared and reviewed by MA, SK, JS and the interviews were conducted by MA having no direct relationship with the participants (34).

Participant recruitment

Participants were theoretically sampled for the interview rounds (35, 32). Participants were contacted following set inclusion criteria specifically comprising physiotherapists and patients living in Nigeria.

Physiotherapist participants were from diverse specialities in diverse work settings, with varying work experience and levels of training. Patients were with LBP of varying severity and any episodic duration spread across the three risk subgroups in the STarT-Back classification and receiving treatment from any health institution. These were approached and invited by the physiotherapists who have agreed to participate (36).

Sociodemographic details comprising age, sex, work status were obtained from participants. The STarT-Back risk categories were determined. Physiotherapy participants provided details on age, sex, previous training, working experience (overall/with LBP-patients), qualification and work-setting. Participants were recruited until saturation was reached (37).

For the introduction of the concept of stratified care to patients and physiotherapists, interactive videos were prepared and presented before the interviews (19). The videos were designed with a voice layover and interactive questions embedded within to keep participants involved and to help them understand key aspects. These videos were prepared following the guidelines for instructional videos by Norman (2017) (38) with input from the STarT-Back Tool development group in the UK. The content was developed for two specialized videos for the participating physiotherapist and one video for the patient with underlying principles from Main et al. (2012) (39) as shown in Table 1. Patient video was co-produced with patient input, using lay understandable language and physiotherapists videos were co-created with input from physiotherapists. Stakeholder input informed the content, timing, use of language and length of presentation (40).

Data Collection

Interviews were conducted over the phone at the convenience of the participants.

For peer review only

Table 1: Content of the introductory presentation adapted from Main et al. (2012).

For patients	For physiotherapists
The rationale for using SB approach, including potential clinical benefits	The rationale for SB approach
	Psychosocial barriers to progress
Psychosocial components of LBP	Grouping using the SB Tool
Description of the SB approach	- Content and purpose
- Content and purpose	- Definition of subgroups
- Definition of subgroups	How to use and score the tool
- Matched treatments	Scientific underpinning of the approach
Treatment outcome using the approach	Clinical and economic benefits

SB: STarT-Back, LBP: Low Back Pain

The interview guides for patients and physiotherapists were prepared in four phases; brainstorming, collection, sorting, examining questions and consultations as described in research (41, 42). The questions were drafted in the backdrop of categories in the Consolidated Framework for Advancing Implementation Research (CFIR) (43).

Patient guideline: Developed questions were re-phrased to simpler forms for patients and to reveal the patient's perspective (online supplemental table 1). Then sorted repeatedly based on categories (44), allowing for open but thematically structured interview questions (45). They were then reviewed by a patient with LBP and modified by inputs (40). Four key questions were developed with additional maintenance questions and follow-up questions for patients with LBP.

Physiotherapist guideline: The questions for physiotherapists were re-phrased to reflect physiotherapists' perspectives (online supplemental table 2). Two physiotherapists with experience in qualitative interviewing reviewed the questions and made inputs. These were adopted into the guideline ready for use. Four key questions were developed with additional maintenance questions and follow-up questions for physiotherapists.

A pilot test of the interview guidelines was carried out on a sub-sample of the target population (n=5) (3 physiotherapists and 2 patients with LBP). Verbalisation feedback by participants informed the modification of the interview questions and approach strategy, as exemplified in a study by Pepper et al. (2018) (46). The focus of the interviews was hence modified simultaneously with theoretical sampling as outlined in Box 1 (40) .

The telephone interviews were conducted by MA following the pattern of a problem-centred interview (45). The interviews were tape-recorded and handwritten notes were taken during the interviews. Verbal consent was received for each participant before commencement. Questions were flexibly rearranged to express a dialogue and improve the flow of the interview, beginning with warm-up questions. These questions drew insight into the expert's background to create a context and relaxing warm-up atmosphere.

For peer review only

Box 1: Patient and public involvement according to BMJ guidance (40)

How was the development of the research questions and outcome measures informed by patients?

The circulatory process of qualitative research using grounded theory ensured that patients and physiotherapists as public partners played a key role in theoretical sampling and developing the interview guideline. The use of the STarT-Back tool was informed by collaborative efforts and consultations of research team members including KS working intensively with patient consultants.

How did you involve patients in the design of this study?

Patients were consulted in development and tailoring of the information videos and two patients were part of the pilot team for the interview guidelines. They informed the burden of information, time allocated for participation, direction of further enquiry under the grounded theory methodology.

Were patients involved in the recruitment to and conduct of the study?

Patients contributions and referrals contributed to the ideas guiding further recruitment. Patients and physiotherapists as public partners helped in the tailoring of the interview questions comprising key questions, maintenance and follow up questions. This included adapting wording, and sentence structures and including simple patient specific information. After the interviews, summary notes were read back to patients for confirmation and they gave feedback on the interviews.

Notes were summarised back to the participants after each interview and memos were written about each interview. This process was repeated for the various rounds until saturation, where no new categories emerged (37).

Data Analysis

A simple transcription method as described by Dresing and Pehl (2012) was used (47) with the help of Easy transcript software (48). Pseudonyms were used in each transcript making it recognisable during the coding process and differences between patient and physiotherapist. Transcripts were plain text only to facilitate the coding process and were read separately by a second researcher with experience in qualitative analysis.

Coding

Transcripts were read and re-read by MA and SK. A detailed coding agenda was developed with code and category descriptions to guide the coding process (online supplemental table 3) and distributed to all authors for feedback. Thereafter, categories were identified inductively.

Code labels were lifted directly from participants quotes identifying descriptive categories first (32). Higher-level abstract categories were systematically identified separately by the MA and SK as coding progressed, tested against the data and consolidated repeatedly. Variations among categories were derived as recommended by Sbaraini et al. (2011) (49). The data was broken down, intrinsically compared to explain variations and re-combined in an abstract manner describing the relationships.

For peer review only

The line-by-line open coding method was used while the RQDA package in the R software was used for the coding process (50).

Ethical approval

Ethical approval was obtained from the Trier University of Applied Sciences and the Obafemi Awolowo University Teaching Hospital, Ile-Ife Nigeria (registration ID: IRB/IEC/0004553). Approval for telephone interviews was also obtained from the Trier University of Applied Sciences. All participants were sent information sheets and provided written informed consent before participation.

Results

A total of 33 participants were contacted and at different stages by email, and 25 consented to participate. Of the eight remaining participants, two patients were uninterested, while six physiotherapists could not find the time for the study. This resulted in a response rate of 81% for patients and 70% for physiotherapists. All consented participants were interviewed, 14 male and 11 female (12 physiotherapists and 13 patients), lasting an average of 50 minutes per individual. Physiotherapists had backgrounds in various specialisations and represented a variety of work settings. Patient participants were classified as ‘high risk, medium and low’ based on the STarT-Back tool.

Tables 2 and 3 show detailed information on participants’ practice experience, experience with LBP, qualification, speciality, work setting and STarT-Back classification.

Table 2: Patients sample description

Set[a]	Title[b]	Age group (y)	Sex	Work status	STarT-Back Classification[c]
1	Pat1	>50-60	M	Paid work	High risk
2	Pat2	>50-60	M	Paid work	Low risk
	Pat3	>40-50	M	Paid work	High risk
	Pat4	>70-80	M	Retired	High risk
	Pat5	>60-70	M	Retired	Low risk
3	Pat6	>60-70	F	Self-employed	Medium risk
	Pat7	>30-40	F	Self-employed	Medium risk
	Pat8	>40-50	M	Paid work	High risk
	Pat9	>40-50	F	Self-employed	High risk
	Pat10	>50-60	F	Self-employed	Medium risk
4	Pat11	>30-40	F	Paid work	High risk
	Pat12	>40-50	M	Paid work	Medium risk
	Pat13	>30-40	M	Self-employed	High risk

For peer review only

Pat: Patient

a First, second, third and fourth iterative interview rounds.

b Patients' pseudonymous designation comprising the numerical order of interviews used to reference patients' quotes.

c Based on the STarT-Back Tool. Patients who score 0-3 were allocated to the low-risk subgroup, 4-9 but 3 or fewer of the five sub-scale are medium risk, 4 of 5 on the psychological subscale are high risk. Higher scores indicate an increasing complexity of the condition.

Table 3: Physiotherapists sample description

Set[a]	Title[b]	Age group (y)	Sex	Practice	Experience with LBP	Level of education	Area of interest	Work Setting
1	PT1	>30-40	M	>10-20	>10-20	MSc	Orthopaedics, Private Practice	Physiotherapy Training Institute
	PT2	>30-40	M	>0-10	>0-10	BSc	Orthopaedics, Private Practice	Primary Health
	PT3	>20-30	F	>0-10	>0-10	BSc	Women's Health	Private Practitioner
	PT4	>30-40	M	>0-10	>0-10	PhD	Orthopaedics, Educator	Physiotherapy Training Institute
2	PT5	>30-40	M	>0-10	>0-10	BSc	Orthopaedics, Paediatrics	Teaching Hospital
	PT6	>30-40	M	>0-10	>0-10	BSc	General Practice	Specialist Hospital
	PT7	>30-40	F	>0-10	>0-10	BSc	Neurology, Paediatrics, Geriatrics	Teaching Hospital
3	PT8	>30-40	F	>10-20	>10-20	BSc	CRP, Orthopaedics	Teaching Hospital
	PT9	>30-40	M	>0-10	>0-10	BSc	Paediatrics	Teaching Hospital
	PT10	>20-30	F	>0-10	>0-10	MSc	Ergonomics, Occupational	Corporate Organisation
4	PT11	>30-40	F	>0-10	>0-10	BSc	CRP	Teaching Hospital
	PT12	>30-40	F	>10-20	>10-20	MSc	Orthopaedics	Physiotherapy Training Institute

CRP: Cardiorespiratory physiotherapist, BSc: Bachelor of Science; MSc: Master of Science; PhD: Doctor of Philosophy. PT: Physiotherapist,

a first, second, third and fourth iterative interview rounds.

b Physiotherapists' pseudonymous designation comprising the numerical order of interviews used to reference physiotherapists' quotes.

For peer review only

The perspectives of physiotherapists and patients to the implementation of a tailored stratified care approach were captured in seven main hierarchical categories:1) recognising the need for change, 2) acceptance of innovation, 3) resistance to change, 4) adapting practice, 5) patients learning journey, 6) trusting the therapist and 7) needing conviction (Table 3). These categories varied based on four themes: a) tradition of treatment, b) evolution to a new system, c) experiences, d) strategies for implementation (Table 4). While some categories focused only on physiotherapists others focused only on patients and still others on both physiotherapists and patients.

Table 4: Categories and Themes of variation

**Categories**	**Themes of variation**<br>**a)**<br>**Tradition of treatment**	**b)**<br>**Evolution to a new system**	**c)**<br>**Experiences**	**d)**<br>**Strategies for implementation**
**1) Resistance to change**	Incentives attached to the usual practice	Overcoming patients' expectations	Organisational culture	Targeting attitudinal change
**2) Acceptance of innovation**	Ease of transition	Open to new knowledge	Steps towards optimising practice	Work Settings
**3) Adapting practice**	Need of standard for regulating the practice	Cultural adaptations	Use of communication	Awareness for patients and PT
**4) Patient's Learning Journey**	Needing a complement to usual care	Recognising unhelpful treatments	Learning to live with pain	Taking charge
**5) Trusting the PT**	Getting some help	Learning with practice	Therapists doing their best	Co-operating with the PT
**6) Needing conviction**	Reliance on investigations	Patient education	Self-discovery	Struggle against false information
**7) Recognising the need for change**	Lack of training to give psychologically informed therapy	Embracing a different approach	No complete relief	The role of funding

Resistance to Change

This is a mixed category as it contains aspects focusing on patients and physiotherapists. It describes the resistance perceived and experienced by physiotherapists and patients concerning the implementation of the stratified care approach. It further describes the physiotherapists' and patients' perception of challenges to the fundamental change and strategies to overcome resistance.

Participants opined that physiotherapists' *tradition of treatment* was a major challenge, it seemed to be an overwhelming issue sustained by *incentives attached to the usual practice*. Key aspects are ego, self-confidence in their current practice experience and finance. There is a long-held tradition of treatment

For peer review only

with non-evidence-based methods, diagnosis and radiographs that the patient has got used to and this tradition remains deeply entrenched in practice.

> *PTm3: You need more patients to have more income, so the more the patients the more the income, so that is the case.*

The variation *strategies for implementation* indicates *targeting the attitude of physiotherapists and patients* which is seen by participants as a major strategy to improve the chances of successful implementation. The physiotherapist and patients see themselves as mostly complacent. The patients blamed the physiotherapists for not being attentive to their needs and the physiotherapists blamed the patient's attitude as a deterrent for being unable to improve their condition.

> *PatM3: I know patient attitude matters…but at least play your role and if the patient misbehaves you know your job is done. If your recommendations are practicable you will get a good attitude from them.*

Participants' perceptions varied in relation to an *evolution to a new system* as therapists see *patient expectation as* a primary deterrent to implementing stratified care that needs to be addressed. It feels to them like all their suggestions and efforts to optimise practice will not work because the patients come with a certain expectation. They expect a strong touch, massage or something physical that would bring the pain down to zero. There is, however, an enabling situation seen by therapists and patients that these expectations can be adjusted and overcome.

> *PTf4: Then for the patient, … they might not be satisfied because they are expecting more from you'…if you don't explain why you have to do that and you just do it they won't feel happy. They feel they need some form of hard touch, strong touch.*

From the experiences of physiotherapists and patients, the preponderant *organisational culture* makes treatment less efficient and less effective. They have limitations in their ability to ease the changes beyond a certain point, like organising situations, follow-ups or influencing patients' working conditions due to a systemic resistance.

> *PTm6: An example is the government trauma centre I worked in, they had made a move to implement an electronic documentation system for use, but eventually, it never saw the light of day, because the attitude towards it was terrible from the management and recipients.*

Acceptance of Innovation

This is a category focusing on physiotherapists. It describes a positive reaction from physiotherapists to the idea of implementation of stratified care, highlighting their perspectives on the best settings and enabling conditions for implementation with inputs from patients.

Some physiotherapists reported similarity of stratified care to what they already know comprising the usual *tradition of treatment* and practice in their management of LBP, they have background knowledge of the biopsychosocial approach and various treatments for patients. These are individual aspects of

For peer review only

stratified care and participants reported that this *eases the transition* and aids them to adopt the stratified care in its entirety.

> *PTm5: I won't say the knowledge of psychosocial problems associated with LBP is low among physiotherapists any longer. I think there has been a lot of awareness and people are getting to know about those issues. We have been always talking of psychosocial aspects and the likes. I think this brings it more to the fore...I think using this approach helps… it's going to be easier for them to incorporate them into routine practice.*

Physiotherapists also describe an *evolution to a new system* comprising some form of realisation, that this approach is fundamentally new, uncommon and not used in its entity in clinical practice, hence they *were open to new knowledge* describing stratified care as an improvement to current care and welcome development.

> *PTf4: It is very different from what we do here, the treatment here is based on diagnosis, this approach is based on prognosis so yeah, it is different.*

From their *experiences*, participants came across situations where they made *steps towards optimising practice*, instances where innovations, ideas or approaches have been introduced in their various work settings, the process that helped and the positive responses from colleagues. Key aspects were the use of clinical discussions, through physiotherapists' cadre and time-saving measures for the patient. They think the stratified care being introduced in a similar stepwise manner will be successful.

> *PTf11: Many have been introduced to us by different people, I have even tried to introduce one and it was not a big deal if you have a proper literature backup. once it is adopted everyone used it, for instance, when Kinesio-taping was introduced to us, the same thing happened. it was accepted and then everyone practised using it.*

Participants expressed differing perspectives on *strategies for implementation* of stratified care in relation to the most viable *work setting* to target since there exist different situations and ideologies at the private and government establishments. Participants felt it would be more applicable if targeted at the government hospitals since the therapists are paid by the government a fixed amount and more treatments do not lead to more money.

> *PTf10: Then for physiotherapists, working in government hospitals they might be encouraged to use the approach, but not therapists in the private sector… this approach will reduce their revenue and will not be welcomed.*

Adapting Practice

This category applies to patients and physiotherapists. It describes the suggestions on adaptations in terms of creating awareness and modification of the approach to suit the Nigerian context and enhance its use. Here, participants highlight their opinions and experiences on ways to adapt the stratified care approach.

For peer review only

Participants speak of *Socio-cultural adaptations* relating to language, religion, the culture of respect and hierarchical implementation from the seniors to juniors. This was highlighted as a viable means of adapting practice at the introductory phases in attempting the evolution to a new system.

> *PTm9: Our environment is peculiar things have to go through certain paths, we need to convince the seniors. then it can be implemented on a departmental level. It is practically impossible for one person to champion it.*

Most participants mentioned that the *use of communication* was a major way to inform adaptation and improve the patient-therapist relationship. From their *experiences*, when communication between patient and physiotherapist was used, it was seen to produce positive effects.

> *PatF7: In a way, some part of my treatment helped me psychologically, there are days he would talk to me so I can help myself.*

Participants argued that the absence of a standard of practice embedded in their usual *tradition of treatment* and tailored to the needs of Nigerians was a major gap in practice. This situation led to a clamouring for *standards to guide practice and regulation.* Adherence to such a standard of practice would enhance adaptation for implementation.

> *PTf7: In Nigeria, we do not have a standard way of doing things. what hospital A is doing is different from what Hospital B is doing… everyone is just doing what they want.*

Participants confirmed that creating *awareness for patients and physiotherapists* about the approach is a major *strategy for implementation*. While some opined this can be done at undergraduate and post-graduate levels for physiotherapists, patient's awareness was achievable through outreaches.

> *PTf4: Patients…should know that physiotherapy involves advice too and they will feel better. it would also help physiotherapists a lot if they are aware of the approach.*

Patients Learning Journey

This category focuses on patients and physiotherapists. Here, patients have experienced care from various fronts and have formed their opinions about what they have experienced to be helpful to their condition. This was corroborated by the physiotherapist opinions about the patients' learning experiences from their perspective.

Patients here describe having undergone a learning journey, they have gathered added experiences. Participants explain how the debilitating condition of the patient's experiences has resulted in personal difficulties. They resolved in their minds to change their outlook, *learn to live with pain* and accept the condition, and this has improved their outlook.

> *PatM3: It affected me psychologically, I was worrying but I have learned to live with it, I think I am better at managing myself …so also, I have learned, I have concluded that no kind of*

For peer review only

intrusive treatment can work for this my case, that I just need to be careful with how I live my life.

For patients, the usual *tradition of treatment* care was seen to be insufficient and there are aspects that patients feel would *need some form of compliment,* they feel this can be achieved by treatment from traditional bone setters which is seen as a compliment or an alternative.

PatF11: It is not everything science can explain…as some hospitals do not have machines, these strong (traditional) hands can be used instead…so some things the hospital cannot handle, these (bone setters) know where and where to touch.

Participants report that over the past recent years, patients have gradually become mentally equipped to *recognise unhelpful treatments* by themselves and developing the ability to differentiate between what is beneficial and what isn't. This realisation is seen as a major stride in the *evolution to a new system* and a viable enabler to the implementation of stratified care.

PTf8: When they visit these, they come back (from traditional bone setters' treatments) with complications that I have to deal with so it is a barrier, and over the years it was more, but recently the percentage is coming down now it should be about 30 percent but back then some years ago it was about 70 percent. it makes their treatment longer.

Patients mentioned that they have gathered knowledge of self from various treatment exposures, from self-care, traditional bone setters' treatments. They have now decided that *taking charge* of their treatment is a vital *strategy for implementation* and ultimately a strategy to help themselves.

PatM5: My advice is that people should exercise and remain active. I know the positive side of exercise, it is extremely good. That is why I don't miss it at all, no matter what happens.

Trusting the Therapist

This category focuses on patients. It describes the patient's views of the physiotherapist as a dynamic expert with whom they need to give their trust and co-operation based on the efforts of the physiotherapist to help their situation and their experiences with treatments.

Patients agreed that some aspects of the usual *tradition of treatment* especially those focused on exercise and advice provided some relief to patients, they reported they *got some help* from it. Even though not permanent they relate some improvement in their condition and trust that based on this result more can be expected.

PatF9: Just the exercise he prescribed, stretching on the bed, but as I was doing it initially I was feeling the pain much but later it reduced, formerly I counted just 20 counts, but now I can count up to 40 without feeling much pain, the physiotherapist gave me the exercise.

Most patients expressed that the physiotherapists will gradually *evolve to the new system* in their practice and that some therapists are already on the right track, *learning to change their approach with practice*. They feel exploring ideas like this through research is needed to evolve more.

For peer review only

> *PatM5: This can be the case sometimes, in a bid to solve a problem they solve others. That's how they learn, they learn by practice. Experience is the best teacher, so most times experience comes into play.*

The patients acknowledge that the *physiotherapists are doing their best* by putting in efforts to optimise care in ways they can and they appreciate the efforts, they notice from their personal *experiences* that when some more is done to help their condition it encourages them to focus more on the efforts.

> *PatM4: Yes, for me, they did the best they could I would say, for the treatment too… they used to tell us and give us hope, the possibility to get better. they have human sympathy, they advise us sometimes that helps.*

The patients consider the physiotherapist's role as providing care and support for the patient when needed and their role as patients is not to interfere with the process. As a *strategy for implementation,* they felt they need to *co-operate with the physiotherapist* but largely leaving the aspect of care for the therapists.

> *PatF1: When you know this is what your clinician wants to achieve then anything he tells you would do it, do it so that you can get a good result because when you do not follow up well you might think it did not work because didn't follow instructions well, so if you co-operate you would be able to achieve what you want.*

Needing Conviction

This category focuses on patients. It describes the patient's exposure to false information sources and the need for physiotherapists to provide trustworthy education to get their full cooperation. This equips patients on their road to self-discovery and guides them to conviction.

Participants argue that for patients to be able to accept change, they need to be informed properly to a level of conviction. Patients have multiple sources of information and this needs to be presented clearly and reasonably to influencing attitudes and lifestyle. If *patients can be educated* and the procedures explained properly, it will go a long way to influence their expectation, help them become more receptive to a change in approach and a viable enabler in the *evolution to a new system.*

> *PatM2: The patient needs such conviction, he should not leave with the feeling that 'that clinician does not have my interest and doesn't want to treat me let me not waste my time to go back there…. he has to be convinced he is not just assigned to that group because you feel that his problem is not serious enough to be attended to.*

Physiotherapists agree that the *tradition of treatment* they are already used to involves *reliance on investigations*, a diagnosis-based approach, using radiographs and passive treatments. Since the patients have become used to that, they need some form of conviction to accept the change. Some patients enjoy passive treatments and would not only prefer that but insist it is the right thing to do.

For peer review only

PatM3: The only decision you make that will make sense to me is based on evidence from X-rays, tests and the likes not just based on your opinion.

Patients relate situations when they needed to be convinced from within through some form of *self-discovery*, even though their therapist tried by advising them they still needed to see for themselves to be successfully convinced to overcome the initial resistance. Based on their *experiences*, they relate that their outlook changed and they experience better outcomes.

PatM5: They told me to check my body mass index, but I told them it might not be right, for the past 30 years I have never gone below 80 kilograms, so going to that weight I don't know how I would look like. It would be terrible…I did physiotherapy, took medications and went to hospitals several times. Until I discovered that my weight was the cause… I can manage myself now.

Patients emphasise that there is a variety of information sources and many of which are misleading. These can come from other patients, from the internet or clinicians with certain motives. There is a constant *struggle against wrong information* by the patients which the therapists need to drive against to optimise care. To combat this, information pools, research banks were suggested as viable *strategies for implementation.*

PatM5: Yes, although if it is the general internet sometimes it gives false information too. Information pool is needed, as a research bank for publications available to all clinicians so that anyone who needs it can go into such pool in managing their patient.

Recognising the Need for Change

This category focuses on physiotherapists and patients. It describes participants' attention to deficiencies and deterrents in physiotherapists' knowledge and practice, further corroborated by the patient's dissatisfaction with the current treatment outcomes which can be enabled by acceptance of the stratified care approach and proper funding.

Physiotherapists admitted to having gaps in knowledge and *lapses in training* that might affect their ability to deliver optimal biopsychosocial care, this has resulted in sticking to the more familiar *tradition of treatment. This* issue of competency was a major setback. Most physiotherapists felt incompetent to handle such an approach especially the prognostic outlook and psychologically informed therapy.

PTf10: Physiotherapists in Nigeria do not have the training to give psychologically informed therapy, our training does not cover that, except we just use our instinct, most times what physiotherapists do in the name of psychological therapy is just talk and say some things through instinct.

Many patients have *experienced* treatments involving significant efforts with sub-optimal results. Some now seek solutions elsewhere because there has been *no complete relief.* They express disappointment with their situation and frustration with the care they receive. They all feel that things need to change in the way physiotherapists treat patients.

For peer review only

PatM3: I had received treatment for some years until 2016, no relief after physiotherapy and I was contemplating travelling abroad for treatment. Clinicians treating patients need the training to update their treatment methods.

Participants complain of issues of infrastructure and patient load. These issues are systemic constraints that are neither the fault of the patients nor the therapists and have a common underlying denominator as a lack of proper funding. Participants jointly suggest improved *funding* to the health sector as a sound *strategy for implementation* aimed at improving services.

PatM5: There is a proverb in my place 'good soup cost good money'. so, money plays a bigger role in this. It needs to be available for any of these to work.

Physiotherapists and patients agreed they need to recognise the potential to optimise care to be open *to accepting a different approach*. The potential advantages need to be clear to participants, creating an undeniable appeal of a different approach. This is seen as a facilitator for the *evolution of the new system.*

PatM3: Well, I think it is a new approach. It is good, you can then know the kind of treatment and the extent to which you should offer treatment. I believe that this approach goes a long way into what results. It plays a big role.

Discussion

Exploring the perspectives of physiotherapists and patients with LBP in this study has produced insight into the varied barriers and enablers affecting the implementation of stratified care in Nigeria. Findings from this study show that some physiotherapists described stratified care as a familiar approach to be welcomed. This is consistent with previous knowledge, Sanders et al. (2014) reported thoughtful obedience from physiotherapists (51) and Odole et al. (2020) wrote about the willingness of physiotherapists to adopt innovative care methods (52). A reason for this could be having previous knowledge from a standard entry-level training that encompasses basic components of psychosocial care and evidence-based practice with an emphasis on communication as described by Abaraogu et al. (2019) (53). Other studies show physiotherapists had previous knowledge of the biopsychosocial approach (54, 55). This shows the viability of adopting a stratified care approach and ease of transition among physiotherapists.

However, for implementation, the physiotherapists in our study described more challenges with implementing the approach than the patients. Two possibilities could explain this; a negative attitude towards change, as studies reported, clinicians do not always deliver therapy according to the standard they received during training (56, 57, 28). A second possibility could be that they understand the intricacies of use better than the patients (15). Research shows that an understanding of the complexities involved in an intervention creates in itself a capacity for implementation (58, 43) Thus, physiotherapists especially expressed concern on internal and external resistance they face in optimising practice including patient's expectations, patient's attitude, patient-load-to-physiotherapist-ratio and socio-

economic constraints consistent with findings on the implementation of the stratified care in other contexts (59–61).

Despite these perceived barriers, from the interviews, some physiotherapists consider the current practice tradition to be outdated, but this treatment tradition is further enabled by their perception of patients' expectations and the physiotherapist's attitude as well. They are concerned that patients may be unsatisfied with their treatments if they change and thus be swayed towards traditional bone setters, and they might lose funds or relevance while adopting a stratified model of care. Studies show that truly, patients could be unappeased with their treatment with stratified care and this can be significantly linked to their expectations (62). This is consistent with a recent study showing that due to dissatisfaction with their treatment, some patients have gravitated towards treatment by traditional bone setters (63). From the patient's point of view, it can be derived that ideas on treatment goals, preference for passive treatment, how much treatment and the nature of treatment necessary for someone in their condition are being influenced by their clinicians and have strongly shaped their belief system and expectations. This has a bearing on the attitude of patients towards stratified care and their expectations (15).

Patients in this study suggested that it is the responsibility of their clinician to provide the conviction needed to secure their co-operation in stratified care implementation. Some believe that if their clinicians properly introduced it to them via education and meaningful communication, it would be seamless. These findings agree with previous research indicating that patients valued health care professionals who not only listened to them (64, 65), also took time to explain their conditions and options in lay terms and helped them navigate the healthcare system (66). This reveals a mismatch in understanding between patients and physiotherapists and an intrinsic strategy for implementation. The patients have expectations, but these are not as strong as the physiotherapists believe and are fuelled by the therapists themselves. Hence, the therapists can modify the patient's expectations strongly by first, being aware of the areas where these differences in perspectives exist. Studies suggest that with further training, experience and learning from open-minded clinical encounters divergent areas can be identified and managed (67, 68). Secondly, change in physiotherapists beliefs, enhanced communication and strides aimed at patient conviction can contribute to modifying patients' expectations (69).

Another key issue raised as a barrier to implementation is physiotherapist competency in the areas of psychosocial care for high-risk patients. This is a key area of significance paramount in other contexts and among other health practitioners (61, 70). Physiotherapists express concerns about perceived deficiencies in training as seen in previous research (71, 72). Training is required for physiotherapists to deliver a broader biopsychosocial model of care (73) and a key component for successful implementation as shown in research (43, 58). Some question their competence in handling high-risk patients majorly highlighting therapists' need for competency in the psychosocial aspect of patient care.

For peer review only

This is consistent with studies that show physiotherapists today do not feel adequately trained to deal with complex high-risk patients (59, 51).

Patients however trust in the physiotherapist in terms of training and competence. This could be due to their experiences because in seeking help, many have resorted to a variety of care from traditional massage and bone setting to the use of devices. Patients relate they have come to trust their therapists and have discovered from experience that being involved in their care, self-management and an active lifestyle is more beneficial. Such understanding drawn from experience could be a motivation for behaviour change in the direction of implementation (58). This leaves a new role for physiotherapists, providing enablement for their patients instead of dependency (74, 59). Concordant to this, research reveals that this supporting role should include reducing barriers and increasing the patients' capability to self-manage (58).

For physiotherapists, major suggestions made were to enforce paradigm shifts in standardising practice, early undergraduate training and workplace mentorship. These interventions support lasting behavioural change (58) and have a bearing on the success or failure of the implementation efforts (75). Key strategies to implementation are the stepwise introduction of the concept to colleagues especially those of speciality groups relevant to musculoskeletal physiotherapy, emphasising the research results and lobbying the seniors to key in. Some physiotherapists relate that hierarchical implementation has great potential to aid practice among physiotherapists, the driving influence of other clinicians in the higher cadre, senior/supervisors can have a positive impact on the quality of health care delivery offered by junior physiotherapists and hence implementation (76, 77).

Suggestions on where implementation should commence were also paramount in the discussions. Physiotherapists relate that implementation efforts should be focused first on the government hospitals. Regarding the sub-groups, physiotherapists practising in federal settings have the opinions that the aspect of high-risk management will not work in federal settings, due to the high patient load as two studies confirm (78, 79). They feel the assessment and delivery of psychosocial treatment will be a problem due to time. They have the opinion it will work better in the private settings, because private sector physiotherapists have a lesser patient load and perhaps more time. This is consistent with the STarT-Back trial where sufficient assessment time of 45 minutes allocated to patients in the high-risk category contributed to a successful implementation outcome (19).

However, physiotherapists in our study felt the low-risk approach will work better because the therapists are paid by the government and they will welcome the idea as it helps to reduce the patient load (78, 79). The private physiotherapists however opined that they need the patients to keep coming, due to financial reasons, hence the low-risk approach will be undesirable. However, they welcome the high-

For peer review only - http://bmjopen.bmj.com/site/about/guidelines.xhtml

risk approach, as it will improve the quality of care, but the time for assessment will be an issue also as they too have limited time allocated for assessment as research shows (80, 79). Further research reveals that private practice is more satisfactory to patients in terms of interpersonal quality of care, more attentive personnel, and a broader choice of provider (81, 79). The patients in our study, similar to participants in a recent review by Lim et al. (2019), require conviction, quality communication and time for successful implementation (82).

A strength of this study is its design, adhering to a rigorous method to systematically and meticulously draw out comparisons and variations until saturation was reached. The sampling strategy in this study ensured that physiotherapists and patients in both urban and rural settings were contacted giving equal opportunity for referral and response. The sample thus included participants of varied sex and age, physiotherapists from different contexts, patients with acute and chronic health problems, patients who visited the physiotherapist and other health care institutions, and importantly, our sample comprised patients of all risk groups in the STarT-Back risk classifications. These were checked severally until saturation was reached. A challenge that was overcome during this study was getting the participants motivated to engage in uninterrupted productive discussions. This was achieved by emphasizing the relevance of the interviews early during the recruitment process, keeping in mind that stakeholders are often interested in knowledge co-creation and mobilisation when the relevance is succinct (83). Thus, comprehensive and rich interviews were achieved with participants in a comfortable atmosphere.

Conclusion

Perspectives of patients about stratified care differ from that of physiotherapists, and both should be aware of divergent areas needing resolution. Emphasis should thus be placed on quality communication among stakeholders. Such communication requires skill on the part of the physiotherapist and sufficient time to deliver, both of which are considered barriers to implementation of the intervention.

Other meaningful barriers identified in this study include the treatment expectations held by patients regarding the method and success of intervention and the incentivised attachment to the usual practice provided by physiotherapists such as using passive methods.

This study further reveals the use of facilitators like patients' trust, speciality groups and contextual adaptations has the potential to aid the implementation of stratified care. To integrate this into clinical practice, a consensus is required from the physiotherapists on key strategies for tailoring these findings, identifying and prioritising approaches to utilise these facilitators, taking care to overcome the barriers.

For peer review only

Conflicts of interest
The authors declare that there is no conflict of interest

Funding
MA received personal funding from the German Academic Exchange Service (DAAD) and Petroleum Technology Development Fund (PTDF) for his doctoral project.

The authors declare that there is no external source of funding allocated to this project.

Abbreviations

STarT: Subgrouping for Targeted Treatment

SBT: STarT Back Tool

MSK: Musculoskeletal

LBP: Low back pain

Contributions

MA: Designed the study, recruited participants, organised data collection, transcribed, analysed and interpreted the data and drafted the manuscript.

JS: Designed the study, analysed and interpreted the data, critically revised the manuscript.

KS: Designed the study, analysed and interpreted the data, critically revised the manuscript.

CM: Designed the study, recruited participants, critically revised the manuscript.

SK: Designed the study, analysed and interpreted the data, drafted the manuscript, critically revised the document.

MA and SK conceived and supervised all aspects of the project, therefore they are responsible for the overall content as the guarantor.

All authors read and approved the final version of the manuscript.

Acknowledgements

The authors express thanks to Faith Ikpami and Ogbulie Smart for their help to recruit patients; Jonathan Hill and Zipporah Echigeme for their input on the introductory materials; Anneke Wolf for her ideas and help setting up the online survey and Cedric Bender for his ideas on analysis.

Data availability statement

All data relevant to the study are included in the article or uploaded as supplemental information.

For peer review only

References

1. Adegoke OA, Akodu AK, Oyeyemi AL. Work-related musculoskeletal disorders among Nigerian physiotherapists. BMC Musculoskeletal Disorders 2008; 9(1):112.

2. Ayanniyi O, Lasisi O, Adegoke B, Oni-Orisan M. Management of low back pain: attitudes and treatment preferences of physiotherapists in Nigeria. African Journal of Biomedical Research 2009; 10(1):42–9.

3. Oyinloye OI, Abdulkadir AY, Babalola OM. Incidence and patterns of lumbosacral transitional vertebrae, in patients with Low Back Pain in a Nigerian hospital. Nigerian Quarterly Journal of Hospital Medicine 2009; 19(2):95–9.

4. Duthey B. Background Paper. Low back pain: Priority medicines for Europe and the world "A Public Health Approach to Innovation"; 2013 [cited 2020 May 9]. Available from: URL: https://www.who.int.

5. Bello B, Muhammad T. Economic cost of low back pain management in Kano Nigeria. Advances in Life Science and Medicine 2017; 3(1):1–8.

6. German Medical Association. National Programme for Disease Management Guidelines Responsible agencies: Method Report; 2010 [cited 2019 Sep 9]. Available from: URL: http://www.baek.de.

7. Odole A, Akinpelu A, Adekanla A, Obisanya B. Economic burden of low back pain on patients seen at the outpatient physiotherapy clinics of secondary and tertiary health institutions in Ibadan. Journal of the Nigeria Society of Physiotherapy 2011; 18(18):43–8.

8. Buchbinder R, van Tulder M, Öberg B, Costa LM, Woolf A, Schoene M, Croft P. Low back pain: a call for action. The Lancet 2018; 391(10137):2384–8.

9. Hartvigsen J, Hancock MJ, Kongsted A, Louw Q, Ferreira ML, Genevay S, Hoy D, Karppinen J, Pransky G, Sieper J, Smeets R, Underwood M. What low back pain is and why we need to pay attention. The Lancet 2018; 391(10137):2356–67.

10. German Medical Association. National Health Care Guideline Non-specific low back pain long version; 2017 [cited 2020 Jan 10]. Available from: URL: www.kreuzschmerz.versorgungsleitlinien.de.

11. Trauma Programme of Care National Low Back and Radicular Pain Pathway 2017. UK; 2017 [cited 2019 Oct 30]. Available from: URL: https://www.versusarthritis.org.

12. KNGF Guideline. Royal Dutch Society of Physiotherapy [cited 2019 Dec 10]. Available from: URL: http://www.ipts.org.il.

13. Foster NE, Anema JR, Cherkin D, Chou R, Cohen SP, Gross DP, Ferreira PH, Fritz JM, Koes BW, Peul WC, Turner JA, Maher CG, Buchbinder R, Hartvigsen J, Underwood M, van Tulder M. Prevention and treatment of low back pain: evidence, challenges, and promising directions. The Lancet 2018; 391(10137):2368–83.

14. Cruz EB, Canhão H, Fernandes R, Caeiro C, Branco JC, Rodrigues AM, Pimentel-Santos F, Gomes L, Paiva S, Pinto I, Moniz R, Nunes C. Prognostic indicators for poor outcomes in low back pain patients consulted in primary care. PLoS ONE 2020; 15(3):e0229265.

15. Sowden G, Hill JC, Morso L, Louw Q, Foster NE. Advancing practice for back pain through stratified care (STarT Back). Brazilian Journal of Physical Therapy 2018; 22(4):255–64.

16. Whitehurst DG, Bryan S, Lewis M, Hill J, Hay EM. Exploring the cost-utility of stratified primary care management for low back pain compared with current best practice within risk-defined subgroups. Annals of the Rheumatic Diseases 2012; 71(11):1796–802.

17. Fritz JM, Beneciuk JM, George SZ. Relationship between categorization with the STarT Back Screening Tool and prognosis for people receiving physical therapy for Low Back Pain. Physical Therapy 2011; 91(5):722–32.

18. Foster NE, Mullis R, Hill JC, Lewis M, Whitehurst DG, Doyle C, Konstantinou K, Main C, Somerville S, Sowden G, Wathall S, Young J, Hay E. Effect of stratified care for low back pain in family practice (IMPaCT back): A prospective population-based sequential comparison. Annals of Family Medicine 2014; 12(2):102–11.

19. Hill JC, Whitehurst DG, Lewis M, Bryan S, Dunn KM, Foster NE, Konstantinou K, Main C, Mason E, Somerville S, Sowden G, Vohora K, Hay E. Comparison of stratified primary care management for low back

pain with current best practice (STarT Back): A randomised controlled trial. The Lancet 2011; 378(9802):1560–71.

20. Hill JC, Dunn KM, Lewis M, Mullis R, Main CJ, Foster NE, Hay E. A primary care back pain screening tool: Identifying patient subgroups for initial treatment. Arthritis Care and Research 2008; 59(5):632–41.

21. Sowden G, Hill JC, Konstantinou K, Khanna M, Main CJ, Salmon P, Somerville S, Wathall S, Foster N. Targeted treatment in primary care for low back pain: The treatment system and clinical training programmes used in the impact back study. Family Practice 2012; 29(1):50–62.

22. Foster NE, Hill JC, O'Sullivan P, Hancock M. Stratified models of care. Best Practice & Research Clinical Rheumatology 2013; 27(5):649–61.

23. Baxter GD, Chapple C, Ellis R, Hill J, Liu L, Mani R, Reid D, Stokes T, Tumilty S. Six things you need to know about low back pain. Journal of Primary Health Care 2020; 12(3):195–8.

24. Cherkin D, Balderson B, Wellman R, Hsu C, Sherman KJ, Evers SC, Sarah C, Hawkes R, Cook A, Levine M, Piekara D, Rock P, Estlin K, Brewer G, Jensen M, LaPorte A, Yeoman J, Sowden G, Hill J. Effect of low back pain risk-stratification strategy on patient outcomes and care processes: the MATCH randomized trial in primary care. Journal of General Internal Medicine 2018; 33(8):1324–36.

25. Ogbeivor C, Elsabbagh L. Management approach combining prognostic screening and targeted treatment for patients with low back pain compared with standard physiotherapy: A systematic review & meta-analysis Musculoskeletal Care 2021. Epub ahead of print.

26. Bernstein IA, Malik Q, Carville S, Ward S. Low back pain and sciatica: summary of NICE guidance. BMJ Online 2017; 356: 6748.

27. Beneciuk JM, Hill JC, Campbell P, Afolabi E, George SZ, Dunn KM et al. Identifying Treatment Effect Modifiers in the STarT Back Trial: A Secondary Analysis. Journal of Pain 2017; 18(1):54–65.

28. Akodu AK, Kareem RO, Faniyi OR. Management of low back pain: knowledge and adherence to clinical practice guidelines amongst physiotherapists in selected hospitals in Lagos state. Research Journal of Health Sciences 2016; 4(3):203.

29. Igwesi-Chidobe CN, Kitchen S, Sorinola IO, Godfrey EL. "A life of living death": the experiences of people living with chronic low back pain in rural Nigeria. Disability and Rehabilitation 2017; 39(8):779–90.

30. Igwesi-Chidobe CN, Coker B, Onwasigwe CN, Sorinola IO, Godfrey EL. Biopsychosocial factors associated with chronic low back pain disability in rural Nigeria: a population-based cross-sectional study. BMJ Global Health 2017; 2(3):e000284.

31. Willig C, Stainton-Rogers W, editors. The SAGE Handbook of Qualitative Research in Psychology. London England: SAGE Publications; 2008.p 123-140.

32. Corbin JM, Strauss A. Grounded theory research: Procedures, canons, and evaluative criteria. Qualitative Sociology 1990; 13(1):3–21.

33. Tong A, Sainsbury P, Craig J. Consolidated criteria for reporting qualitative research (COREQ): A 32-item checklist for interviews and focus groups. International Journal for Quality in Health Care 2007; 19(6):349–57.

34. Elder NC, Miller WL. Reading and evaluating qualitative research studies. Journal of Family Practice 1995; 41(3):279–85.

35. Palinkas LA, Horwitz SM, Green CA, Wisdom JP, Duan N, Hoagwood K. Purposeful sampling for qualitative data collection and analysis in mixed method implementation research. Administration and Policy in Mental Health 2015; 42(5):533–44.

36. Meerhoff GA, van Dulmen SA, Maas MJM, Bakker-Jacobs A, Nijhuis-Van der Sanden MWG, van der Wees PJ. Exploring the perspective of patients with musculoskeletal health problems in primary care on the use of patient-reported outcome measures to stimulate quality improvement in physiotherapist practice; a qualitative study. Physiotherapy Theory and Practice 2019: 37(9):993-1004.

37. Mason M. Sample size and saturation in PhD studies using qualitative interviews. Qualitative Social Research 2010; 11(3):1–13.

For peer review only

38. Norman MK. Twelve tips for reducing production time and increasing long-term usability of instructional video. Medical Teaching 2017; 39(8):808–12.

39. Main CJ, Sowden G, Hill JC, Watson PJ, Hay EM. Integrating physical and psychological approaches to treatment in low back pain: The development and content of the STarT Back trial's 'high-risk' intervention. Physiotherapy 2012; 98(2):110–6.

40. Staniszewska S, Brett J, Simera I, Seers K, Mockford C, Goodlad S, D Altman, D Moher, R Barber, Dengeri S, Entwistle A, Littlejohns P, Morris C, Suleman R, Thomas V, Tystall C. GRIPP2 reporting checklists: tools to improve reporting of patient and public involvement in research. BMJ (Online) 2017; 358, j3453

41. Heistinger A. Qualitative Interviews – A guide to preparation and implementation including some theoretical notes [cited 2020 Oct 25]. Available from: URL: https://www.uibk.ac.at.

42. Burke LA, Miller MK. Phone interviewing as a means of data collection: lessons learned and practical recommendations. Qualitative Social Research 2001; 2(2):7.

43. Damschroder LJ, Aron DC, Keith RE, Kirsh SR, Alexander JA, Lowery JC. Fostering implementation of health services research findings into practice: a consolidated framework for advancing implementation science. Implementation Science 2009; 4(1):50.

44. Moullin JC, Sabater-Hernández D, Fernandez-Llimos F, Benrimoj SI. A systematic review of implementation frameworks of innovations in healthcare and resulting generic implementation framework. Health Research Policy and Systems 2015; 13(1):1–11.

45. Döringer S. 'The problem-centred expert interview'. Combining qualitative interviewing approaches for investigating implicit expert knowledge. International Journal of Social Research Methodology 2020:1–14.

46. Pepper D, Hodgen J, Lamesoo K, Kõiv P, Tolboom J. Think aloud: using cognitive interviewing to validate the PISA assessment of student self-efficacy in mathematics. International Journal of Research and Method in Education 2018; 41(1):3–16.

47. Dresing, T, Pehl T, editor. Practice book interview and transcription; 2012 [cited 2020 Jan 9]. Available from: URL: https://www.audiotranskription.de/Praxisbuch.

48. Tessier S. From field notes, to transcripts, to tape recordings: evolution or combination? International Journal of Qualitative Methods 2012; 11(4):446–60.

49. Sbaraini A, Carter SM, Evans RW, Blinkhorn A. How to do a grounded theory study: a worked example of a study of dental practices. BMC Medical Research Methodology 2011; 11(1):128.

50. R Development Core Team. R: A tool for statistical computing. Vienna, Austria: R foundation for statistical Computing 2014. [cited 2019 Nov 8]. Available from: URL: http://www.R-project.org.

51. Sanders T, Ong BN, Sowden G, Foster N. Implementing change in physiotherapy: professions, contexts and interventions. Journal of Health Organization and Management 2014; 28(1):96–114.

52. Odole AC, Afolabi KO, Ushie BA, Odunaiya NA. Views of physiotherapists from a low resource setting about physiotherapy at a distance: a qualitative study. European Journal of Physiotherapy 2020; 22(1):14–9.

53. Abaraogu UO, Aguji KR, Duru DO, Okafor UC, Ezeukwu AO, Igwe SE. Physiotherapist-patient communication in entry-level physiotherapy education: A national survey in Nigeria. Hong Kong Physiotherapy Journal 2019; 39(1):77–87.

54. Cowell I, O'Sullivan P, O'Sullivan K, Poyton R, McGregor A, Murtagh G. The perspectives of physiotherapists on managing nonspecific low back pain following a training programme in cognitive functional therapy: A qualitative study. Musculoskeletal Care 2019; 17(1):79–90.

55. Synnott A, O'Keeffe M, Bunzli S, Dankaerts W, O'Sullivan P, Robinson K, O'Sullivan K. Physiotherapists report improved understanding of and attitude toward the cognitive, psychological and social dimensions of chronic low back pain after Cognitive Functional Therapy training: a qualitative study. Journal of Physiotherapy 2016; 62(4):215–21.

56. Odole AC, Oyewole OO, Akinpelu AO. A comparative survey of Nigerian physiotherapists' familiarity with, knowledge of and utilisation of standard outcome measures: 10 years after initial survey. South African Journal of Physiotherapy 2018; 74(1):435.

For peer review only

57. Akindele M, Rabiu M, Useh E. Assessment of the awareness, adherence, and barriers to low back pain clinical practice guidelines by practicing physiotherapists in a low-resourced country. Physiotherapy research international: The Journal for Researchers and Clinicians in Physical Therapy 2019; 25(1):e1811.

58. Michie S, van Stralen MM, West R. The behaviour change wheel: a new method for characterising and designing behaviour change interventions. Implementation Science 2011; 6(1):42.

59. Holopainen R, Piirainen A, Karppinen J, Linton SJ, O'Sullivan P. An adventurous learning journey. Physiotherapists' conceptions of learning and integrating cognitive functional therapy into clinical practice. Physiotherapy Theory and Practice 2020: Epub ahead of print.

60. Holopainen R, Piirainen A, Heinonen A, Karppinen J, O'Sullivan P. From "Non-encounters" to autonomic agency. Conceptions of patients with low back pain about their encounters in the health care system. Musculoskeletal Care 2018; 16(2):269–77.

61. Karstens S, Kuithan P, Joos S, Hill JC, Wensing M, Steinhäuser J, Katja K, Joachim S. Physiotherapists' views of implementing a stratified treatment approach for patients with low back pain in Germany: a qualitative study. BMC Health Services Research 2018; 18(1):1–12.

62. Riis A, Jensen CE, Bro F, Maindal HT, Petersen KD, Bendtsen MD, Jensen MB. A multifaceted implementation strategy versus passive implementation of low back pain guidelines in general practice: a cluster randomised controlled trial. Implementation Science 2016; 11(1):143.

63. Mbada CE, Ojoawo AO, Owoola SO, Okonji AM, Odetunde MO, Adigwe KC Makinde MO, Adegbemigun OD, Fasuyi FO, Idowu OA, Fatoye F. Knowledge and attitude about the practice of traditional bone setters and its use for musculoskeletal disorders in rural areas in Nigeria. Middle East Journal of Rehabilitation and Health Studies 2020; 7(2):e99973.

64. Lewis J, O'Sullivan P. Is it time to reframe how we care for people with non-traumatic musculoskeletal pain? British Journal of Sports Medicine 2018; 52(24):1543–4.

65. Karstens S, Lang S, Saunders B. Patients' views on the implementation potential of a stratified treatment approach for low back pain in Germany: a qualitative study. Health Services Insights 2020; 13:1-8.

66. O'Malley AS, Peikes D, Wilson C, Gaddes R, Peebles V, Day TJ, Janel J. Patients' perspectives of care management: a qualitative study. The American Journal of Managed Care 2017; 23(11):684–9.

67. Bastemeijer CM, van Ewijk JP, Hazelzet JA, Voogt LP. Patient values in physiotherapy practice, a qualitative study. Physiotherapy Research International: The Journal For Researchers And Clinicians In Physical Therapy 2021; 26(1):e1877.

68. Cooper I, Delany C, Jenkins S. Comparing patients' and physiotherapists' views of professionalism and professional standing: an Australian perspective. Physical Therapy Reviews 2016; 21(1):38–73.

69. Le Laekeman M, Sitter H, Basler H. The Pain Attitudes and Beliefs Scale for Physiotherapists: psychometric properties of the German version. Clinical Rehabilitation 2008; 22(6):564–75.

70. Karstens S, Joos S, Hill JC, Krug K, Szecsenyi J, Steinhäuser J. General practitioners' views of implementing a stratified treatment approach for low back pain in Germany: a qualitative study. PLOS ONE 2015; 10(8):e0136119.

71. Abaraogu UO, Onah U, Abaraogu OD, Fawole HO, Kalu ME, Seenan CA. Knowledge, attitudes, and the practice of health promotion among physiotherapists in Nigeria. Physiotherapy Canada 2019; 71(1):92–100.

72. Onyeso OK, Umunnah JO, Ezema CI, Balogun JA, Uchenwoke CI, Nwankwo MJ, Oke K, Bello B, Nwosu I, Adje M. An evaluation of the nature and level of musculoskeletal imaging training in physiotherapy educational programmes in Nigeria. BMC Medical Education 2020; 20(1):252.

73. Cowell I, O'Sullivan P, O'Sullivan K, Poyton R, McGregor A, Murtagh G. Perceptions of physiotherapists towards the management of non-specific chronic low back pain from a biopsychosocial perspective: A qualitative study. Musculoskeletal Science and Practice 2018; 38:113–9.

74. Nijs J, Roussel N, van Paul Wilgen C, Köke A, Smeets R. Thinking beyond muscles and joints: therapists' and patients' attitudes and beliefs regarding chronic musculoskeletal pain are key to applying effective treatment. Manual Therapy 2013; 18(2):96–102.

For peer review only

75. Ulrich G, Homberg A, Karstens S, Mahler C. Attitudes towards interprofessional collaboration in young healthcare professionals. Journal of Interprofessional Care 2019; 33(6):768–73.

76. Ezenwankwo EF, Ezeukwu AO, Abaraogu UO, Manu GP, Mogbolu GA, Ezelioha RN, Ifediora, LP, Uchenwoke CI. Physiotherapy clinical education in the eastern Nigeria: students' and interns' views on clinical educator characteristics, opportunities and learning conditions. European Journal of Physiotherapy 2019; 21(3):153–63.

77. Onigbinde A. The impact of internship programme and its effects on the physiotherapy practice in Nigeria 2007 [cited 2019 Sep 9]. Available from: URL: https://www.semanticscholar.org

78. DO Odebiyi, CB Aiyejusunle, TS Ojo, BA Tella. Comparison of patients satisfaction with physiotherapy care in private and public hospitals. Journal of Nigerian Society of Physiotherapy 2009; 17(1):23–9.

79. Odumodu IJ, Olufunlayo TF, Ogunnowo BE, Kalu ME. Satisfaction with services among attendees of physiotherapy outpatient clinics in tertiary hospitals in Lagos state. Journal of Patient Experience 2020; 7(4):468–78.

80. Onyeso OKK, Umunnah JO, Ezema CI, Anyachukwu CC, Nwankwo MJ, Odole AC, Oke KI, Bello B. Profile of practitioners, and factors influencing home care physiotherapy model of practice in Nigeria. Home Health Care Services Quarterly 2020; 39(3):168–83.

81. Hanson K, Goodman C, Lines JD, Meek S, Bradley D, Mills A. The economics of malaria control interventions. World Health Organization; 2004 [cited 2019 Sep 9]. Available from: URL: http://apps.who.int/iris.

82. Lim YZ, Chou L, Au RT, Seneviwickrama KMD, Cicuttini FM, Briggs AM, Sullivan K, Urquhart DM, Wluka AE. People with low back pain want clear, consistent and personalised information on prognosis, treatment options and self-management strategies: a systematic review. Journal of Physiotherapy 2019; 65(3):124–35.

83. Holmström I, Röing M. The relation between patient-centeredness and patient empowerment: a discussion on concepts. Patient Education and Counseling 2010; 79(2):167–72.

For peer review only

Supplemental Table 1: Interview guideline for patients

Key Question	Maintenance question	Potential follow-up questions
1. Experiences (Warm-up): Tell me something about the LBP treatment you received so far.	- What else?<br>- And so on?<br>- Is there anything else	a) What is your expectation of a successful treatment for your LBP?<br>b) What are your concerns regarding the treatment of LBP?
2. Exploration: What is your opinion on the SB approach?	- What else?<br>- And so on?<br>- Is there anything else?	c) How would you feel being classified into a subgroup based on the SB tool ?<br>d) What would be your reaction if you were offered to be treated with this approach?<br>e) What can you say about the questions in the SB tool?
3. Factors: How does this approach compare with the current treatment you receive?	- What else?<br>- And so on?<br>- Is there anything else?	f) Mention any specific aspect of the SB approach which interests you?<br>g) What are your opinions regarding the tool process?<br>h) What do you think about communication with the therapist in this approach?<br>i) In the video, patients with a low likelihood of poor outcome receive one treatment session with examination and advice, how do you feel about this?<br>j) In the video, patients with a high likelihood of poor outcomes receive physical and psychological treatment, how do you feel about this?<br>k) In your opinion, what do you expect from physiotherapists who use this approach e.g. attitude, training, expertise?<br>l) What is your opinion on a patient's responsibility regarding this approach?<br>m) In your opinion, tell me what is required to use this approach in Nigeria?<br>n) Tell me if other enablers or barriers come to your mind when you think of this approach?
4. Related influences: What do you think might happen when this approach is used?	- What else?<br>- And so on?<br>- Is there anything else	o) How do you think this approach affects you as a patient with LBP?<br>p) How does this approach affect how you view treatment for LBP in Nigeria?

LBP: Low back pain; SB: STarT-Back

Supplemental Table 2: Interview guideline for physiotherapists

Key Question	Maintenance question	Potential follow-up questions
1. Exploration: What is your perception of the SB approach?	• What else?<br>• And so on?<br>• Is there anything else?	a) Tell me something about your current approach to treatment for non-specific LBP.<br>b) How do you consider the idea of classifying patients into subgroups based on the SB tool?<br>c) How would you feel about allocating treatments in this treatment approach?
2. Factors: How does this approach compare with the current treatment for non-specific LBP in Nigeria?	• What else?<br>• And so on?<br>• Is there anything else?	d) Mention any specific attributes of the SB approach that might interest you.<br>e) What can you say about the SB tool?<br>f) What is your opinion about the patient-centeredness of this approach?
3. Strategies: Tell me any possible requirements for implementing this approach in Nigeria?	• What else?<br>• And so on?<br>• Is there anything else?	g) What are your ideas on the handling of the tool?<br>h) In your opinion, what is required for the implementation of the High-risk approach?<br>i) In your opinion, what is required for the implementation of the Low-risk approach?<br>j) What are your ideas about incentives for using this approach?<br>k) Tell me your ideas about training on this approach.<br>l) What comes to your mind when you think about the distribution of this approach in Nigeria?<br>m) Tell me if other enablers and barriers come to your mind when you think of this approach?
4. Related influences: What effects do you think might result from its use?	• What else?<br>• And so on?<br>• Is there anything else?	n) How might this approach affect patients receiving physiotherapy in Nigeria?<br>o) How might it affect interprofessional relations in Nigeria?

LBP: Low back pain; SB: STarT-Back

Supplemental Table 3: Coding agenda with category descriptions

**Category 1: Resistance to change.**<br><br>**Themes of variation**	**Category Description: A mixed category because it describes views from patients and physiotherapists. It describes the resistance perceived and experienced by physiotherapists and patients concerning introducing a stratified care approach. It describes the physiotherapists and patients' perceptions of challenges to the paradigm shift and strategies to overcome resistance.**		
	**Resistance to change**	**Explanation**	**Quotes***
**Tradition of treatment**<br>This describes components of the usual care, highlighting gaps in practice and aspects that can be modified to optimise care.	Incentives attached to the usual practice	Physiotherapists tradition of treatment was a major challenge, it seemed to be an overwhelming issue sustained by Incentives attached to the usual practice. Key aspects are ego, self-confidence in their current practice experience and finance. There is a long-held tradition of treatment with non-evidence-based methods, diagnosis and radiographs that the patient has gotten used to and this tradition remains deeply entrenched in practice.	***PTf7:*** *I would say it has a lot to do with ego, they won't want you to tell them what to do... If I go for training and come back with a new trend, I would start using it immediately, but your colleagues, now avoiding it because it comes from your colleague.*<br><br>***PTf7:*** *Physiotherapists usually shy away when there is a new protocol, implementing it will just be for record purposes, they think it will stress them and is time-wasting.*
**Evolution to a new system**<br>This discusses the key enablers needed to introduce a new practice aimed at optimising care.	Overcoming patients' expectations	Therapists see patient expectation *as* a primary deterrent to the implementation of stratified care that needs to be addressed. It feels to them like all their suggestions and efforts to optimising practice will not work because the patients come with a certain expectation. They expect a strong touch, massage or something physical that would bring the pain down to zero. There is, however, an enabling situation seen by therapists and patients, that these expectations can be adjusted and overcome.	***PatF1:*** *I wish it can go, I wish my pain can go… I think it will need some kind of strong force for it to go, maybe I should like a fall or something to put it in position.*<br><br>***PTm5:*** *I think it's just marrying the expectations of the patient with what the physiotherapist thinks is realistic, and with that, there could be some kind of trade-off.*
**Experiences**<br>This describes instances where attempts to install change have been made and the effects	Organisational culture	From the experiences of physiotherapists and patients, the organisational culture preponderant in Nigeria makes treatment less efficient and less effective. They have limitations in their ability to ease the changes beyond a certain point like organising situations, follow-ups or influence patient's working conditions.	***PatM2:*** *If you tell me to modify my workstation and I know that I cannot do it you are wasting your time. but if you tell me the office seat needs to be changed and in one week someone brings the right one I will have the right attitude.*<br><br>***PTm9****: There might be challenges, the kind of environment and mentality here in Nigeria is different.*
**Strategies for implementation**<br>This highlights the participants' key ideas on strategies for the implementation of the stratified care approach.	Targeting attitudinal change	A change in the attitude of physiotherapists and patients is seen as a major strategy to improve the chances of successful implementation. The physiotherapist and patients see themselves as mostly complacent. The patients blamed the physiotherapists for not being attentive to their needs and the physiotherapists blamed the patient's attitude as a deterrent for being unable to improve their condition.	***PatM8:*** *The period I was there I don't think there was a lot of Patient load, we were not more than ten patients attending physiotherapy so I think it is their attitude that needs to change.*<br><br>***PTf8:*** *Well, I have tried to push it, and it didn't work…some patients have an attitude like you are trying to test their knowledge…so somehow this attitude has affected the use by physiotherapists.*

* Physiotherapists' and patient's quotes with pseudonymous designations comprising the numerical order of interviews.

For peer review only

**Category 2: Acceptance of innovation**<br><br>**Themes of variation**	**Category description: This is a category most relevant to physiotherapists. It describes a positive reaction from physiotherapists to the idea of implementation of stratified care in Nigeria, highlighting their perspectives on the best settings and enabling conditions for implementation with inputs from patients.**		
	**Acceptance of innovation**	**Explanation**	**Quotes***
**Tradition of treatment**<br>This describes components of the usual care, highlighting gaps in practice and aspects that can be modified to optimise care.	Ease of transition	Some physiotherapists reported similarity of stratified care to what they already know (tradition of treatment) and practice in their management of LBP, they have background knowledge of the biopsychosocial approach and vary treatment for patients. These are individual aspects of stratified care and participants reported that this makes it easy to fully adopt the stratified care in its entirety.	***PTf7:*** *You are just giving it a name, it is comparable to what we do already here…. well, now all patients are treated more individually, we do not use the same intervention for every LBP patient anymore we have improved.*<br><br>***PTf11:*** *Yes, just as I said it's not really different from what we do here, …we do not have a name for it but it is essentially the same as what this approach is about, but I can speak for my centre only. because I will say my centre is about the best in Nigeria in terms of the content of practice so far.*
**Evolution to a new system**<br>This discusses the key enablers needed to introduce a new practice aimed at optimising care.	Open to new knowledge	Physiotherapists realising this approach is fundamentally new, uncommon and not used in its entity in practice, yet they were open to new knowledge describing stratified care as an improvement to current care and welcome development.	***PTm1****: So far, I think it is a welcome development because the approach is nice because it tends to categorise individuals with LBP in two categories people that have low risk moderate and high categorising individuals into group will give it a definition into the line of management or better outcome.*<br><br>***PTf4:*** *My perception in summary about the approach is that it is a good one and that it would help physiotherapist a lot.*
**Experiences**<br>This describes instances where attempts to install change have been made and the effects	Steps towards optimising practice	From their experiences, participants came across situations where they made steps towards optimising practice, instances where innovations, ideas or approaches have been introduced in their various work settings, the process that helped and the positive responses from colleagues. Key aspects were the use of clinical discussions, through physiotherapists cadre and time-saving measures for the patient. They think the stratified care being introduced in a similar stepwise manner will be successful.	***PTf7:*** *Since everyone is on board, especially our head of department, he likes such things, if he sees the evidence he would say 'from today we are using this approach and it must be used and expedite the use.*<br><br>***PTm9:*** *(In cases of innovation in my clinical setting) the same strategy, we discuss it at seminars and everyone brings their opinions and decides to use it and see the result. only then can it become a norm.*
**Strategies for implementation**<br>This highlights the participants' key ideas on strategies for the implementation of the stratified care approach.	Work Settings	There were differing perspectives on the most viable work setting for targeting the implementation of stratified care since there exist different situations and ideologies at the private and government establishments in Nigeria. Participants felt it would be more applicable if targeted at the government hospitals since the therapists are paid by the government a fixed amount and more treatments do not lead to more money.	***PTf12:*** *To be honest the government hospitals make it easier to use these approaches and questionnaires. Because most of the private settings didn't have these things available, so most times I have to source for them to use, but in the government hospitals they were printed and ready for use.*<br><br>***PTm3:*** *Especially in the private practice where you need more patients to have more income, so the more the patients the more the income, so that is the case, it means the physiotherapist might not want to use that approach but, in the government-owned hospitals where that might not be the case, they may be more willing to go in that direction.*

LBP: Low back pain
* Physiotherapists' quotes with pseudonymous designations comprising the numerical order of interviews.

For peer review only

**Category 3: Adapting practice**<br><br>**Themes of variation**	**Category Description: This category is relevant to patients and physiotherapists. Here participants highlight their opinions and experiences on ways to adopt the stratified care approach. This category describes the suggestions on adaptations in terms of creating awareness and modification of the approach to suit the Nigerian context and enhance its use.**		
	**Adapting practice**	**Explanation**	**Quotes***
**Tradition of treatment**<br>This describes components of the usual care, highlighting gaps in practice and aspects that can be modified to optimise care.	Need of standard for regulating the practice	Participants highlighted the absence of a standard of practice tailored to the needs of Nigerians. This situation led to a clamouring for standard to guide practice and regulation. Adherence to such a standard of practice would enhance adaptation for implementation.	***PTf7:*** *In Nigeria, we do not have a standard way of doing things. what hospital A is doing is different from what Hospital B is doing… everyone is just doing what they want.*<br><br>***PatM8:*** *With proper supervision, if the hospital management focuses on what happens in the hospital I am sure there will be room for improvement, for both the staff and the facility.*
**Evolution to a new system**<br>This discusses the key enablers needed to introduce a new practice aimed at optimising care.	Cultural adaptations	Socio-cultural adaptations relating to language, the culture of respect and hierarchical implementation from the seniors to juniors was highlighted as a viable means of adapting practice at the introductory phases.	***PatF5****: The clinician speaks our language…reads to her…or when she comes home the children do.*<br><br>***PTm2:*** *When the chiefs and assistant directors in each unit champion this, then junior physiotherapists will use it.*
**Experiences**<br>This describes instances where attempts to install change have been made and the effects	Use of communication	Communication was seen as a major way to inform adaptation and improve the patient-therapist relationship. When communication between patient and physiotherapist was used it was seen to produce positive effects.	***PTf8****: It is feasible, I always use conversations to help my Patients see the benefits.*<br><br>***PatM2:*** *Well it is communication; a patient needs to know why they are allocated to a particular group.*
**Strategies for implementation**<br>This highlights the participants' key ideas on strategies for the implementation of the stratified care approach.	Awareness for patients and physiotherapists	Participants saw creating awareness about the approach as a major strategy for implementation. While some opined this can be done at undergraduate and post-graduate levels for physiotherapists, patient's awareness was achievable through outreaches.	***PatF9****: And awareness…when I went for their free medical seminar I was able to meet the physiotherapist guy that came for outreach, … which encouraged me.*<br><br>***PTF9:*** *Also, there should be health promotion done to create awareness about it.*

* Physiotherapists' and patient's quotes with pseudonymous designations comprising the numerical order of interviews.

For peer review only

Category 4: Patient's Learning Journey<br>Themes of variation	**Category Description: This category is relevant to patients and physiotherapists. Here, patients have experienced care from various fronts and have formed their opinions about what they have experienced to be helpful to their condition. This was corroborated by the physiotherapist opinions about the patients learning experiences from their perspective.**		
	**Patient's Learning Journey**	**Explanation**	**Quotes***
**Tradition of treatment**<br>This describes components of the usual care, highlighting gaps in practice and aspects that can be modified to optimise care.	Needing a complement to usual care	For patients, the usual care was seen to be insufficient and there are aspects that patients feel would need some form of compliment, they feel this can be achieved by treatment from traditional bone setters care which is seen as a compliment or an alternative.	***PTm5***: *Most people prefer to go to bonesetters and the likes because the people around them would have told them that this is what someone used and the person got better so.*<br><br>***PatM2:*** *There is a lot in it (traditional bone setters care) that complements orthodox medicine'…I can manage my pain better.*
**Evolution to a new system**<br>This discusses the key enablers needed to introduce a new practice aimed at optimising care.	Recognising unhelpful treatments	Participants report that over the past recent years, patients have gradually become mentally equipped to recognise unhelpful treatments by themselves and developing the ability to differentiate between what is beneficial and what isn't. This realisation is seen as a viable enabler to the implementation of stratified care.	***PatM2:*** *The former treatment was more like going thru a particular regimen and go back to the same condition that brought the pain but the approach …suggests lifestyle changes which if sustained gives permanent relief.*<br><br>***PatM3:*** *Well, to me I tried it and it made matters worse, so I wouldn't advise anyone to do that, to me that is a no-go area because they are working blindly.*
**Experiences**<br>This describes instances where attempts to install change have been made and the effects.	Learning to live with pain	Participants explain how the debilitating condition of the patient's experiences have resulted in personal difficulties. They resolved in their minds to change their outlook and accept the condition and this has improved their outlook.	***PatF9***: *I feel depressed most times, it affects me so much, … I think it is what I would work on now, psychologically I know it is affecting me because I am thinking so much about it and it is giving me so much concern.*<br><br>***PatM3:*** *It affected me psychologically, I was a worrying bit I have learned to live with it, I think I am better at managing myself …so also, I have learned, I have concluded that no kind of intrusive treatment can work for this my case, that I just need to be careful with how I live my life.*
**Strategies for implementation**<br>This highlights the participants' key ideas on strategies for the implementation of the stratified care approach.	Taking charge	Patients have gathered knowledge of self from various treatment exposures, from self-care, traditional bone setters' treatments. They have now decided that taking charge of their treatment is a vital strategy to help themselves.	***PatF7:*** *Yes, what I can generally say is it helped me to know how to better manage the back by myself.*<br><br>***PatF9:*** *I would love to do it (treatment) myself…rather than going to the hospital.*

* Physiotherapists' and patient's quotes with pseudonymous designations comprising the numerical order of interviews.

For peer review only

**Category 5: Trusting the physiotherapist**<br><br>**Themes of variation**	**Category Description: This category is most relevant to patients. It describes the patient's views of the physiotherapist as a dynamic expert with who they need to give their trust and co-operation based on the efforts of the physiotherapist to help their situation and their experiences with treatments.**		
	**Trusting the physiotherapist**	**Explanation**	**Quotes***
**Tradition of treatment**<br>This describes components of the usual care, highlighting gaps in practice and aspects that can be modified to optimise care.	Getting some help	The aspects of the usual care focused on exercise and advice was seen to provide some relief to patients, even though not permanent they relate some improvement in their condition and trust that based on this result more can be expected.	***PatF7****: The therapy I underwent helped though because before I started the treatment I could not even lift myself from the bed …but the first week of the therapy I would say I experienced relief.*<br><br>***PatM3****: I started getting relief because my clinicians were trained, even the advice I received worked to some extent. So, I believe that clinicians treating patients need the training to further update their treatment methods.*
**Evolution to a new system**<br>This discusses the key enablers needed to introduce a new practice aimed at optimising care.	Learning with practice	The patients feel that the physiotherapists will gradually evolve in their practice and that some therapists are already on the right track, learning to change their approach with practice. They feel exploring ideas like this through research is needed to evolve more.	***PatM3:*** *What you are doing is good, research is the main factor so just keep it up. there is no way around it, to give patients the best available treatment there is only one way to make sure of it and that is research. continue to make research that's the future of the medical practice.*<br><br>***PatM4****: It is possible they know these things already, because you know they are experts and they have been dealing with people having the same issues with me for a long time, so they can juxtapose and see if that is the problem or a factor.*
**Experiences**<br>This describes instances where attempts to install change have been made and the effects	Therapists doing their best	The patients acknowledge that the physiotherapists are putting in efforts to optimise care in ways they can and they appreciate the efforts, they notice when some more is done to help their condition and that encourages them to focus more on the efforts.	***PatF9:*** *That therapist tried, he did a lot, I told him I appreciated his work very well…. they tried a lot, I am satisfied with their level of experience.*<br><br>***PatM8****: He talked about some exercise but he advised me majorly about my sleeping position, which has helped so much.*
**Strategies for implementation**<br>This highlights the participants' key ideas on strategies for the implementation of the stratified care approach.	Co-operating with the therapists	The patients consider the physiotherapist's role as providing care and support for the patient when needed and their role as patients is not to interfere with the process. They feel they need to co-operate with the physiotherapist but largely leaving the aspect of care for the therapists.	***PatM5****: For me, I believe that their field and they are the specialists. I can't say, I try as much as possible not to go into areas where I have low competence.*<br><br>***PatM3****: You know when the pain becomes unbearable, you need to consult a specialist, at least to know what is happening and have their support.*

* Patient's quotes with pseudonymous designations comprising the numerical order of interviews.

For peer review only

**Category 6: Needing conviction**<br><br>**Themes of variation**	**Category Description: This category is most relevant to patients. It describes patients exposure to false information sources, hence the need for physiotherapists to provide trustworthy education to get their full co-operation. This equips patients on their road to self-discovery and guides them to conviction.**		
	**Needing conviction**	**Explanation**	**Quotes***
**Tradition of treatment**<br>This describes components of the usual care, highlighting gaps in practice and aspects that can be modified to optimise care.	Reliance on investigations	Physiotherapists are already used to a diagnosis-based approach, using radiographs and passive treatments. Since the patients have become used to that, they need some form of conviction to accept the change. Some patients enjoy passive treatments and would not only prefer that but insist it is the right thing to do.	***PTf10****: There is the likelihood that patients will not accept this, they will go after the treatment to another therapist…some tell you how much they enjoy the sessions…they keep coming back to enjoy the sessions.*<br><br>***PatM8****: Investigations are not done. I had to muscle my way to do an MRI by myself, nobody told me about that I just knew it was necessary and I decided to do that.*
**Evolution to a new system**<br>This discusses the key enablers needed to introduce a new practice aimed at optimising care.	Patient education	For patients to be able to accept change, they need to be informed properly to a level of conviction. Patients have multiple sources of information and this needs to be presented clearly and reasonably to influence attitudes and lifestyle. If patients can be educated and the procedures explained properly, it will go a long way to influence their expectations and help them become more receptive to a change in approach.	***PTm9****: It is possible they might also accept the treatment if it costs less but they need to be educated, in such a situation one has to spend more than that ten minutes to convince them.*<br><br>***PatM2****: You see, the patient attitude is determined by the amount of information that he has, so if you can clearly explain the approach and how it works you are likely to get a positive attitude from the patient.*
**Experiences**<br>This describes instances where attempts to install change have been made and the effects	Self-discovery	Patients relate situations when they needed to be convinced from within, even though their therapist tried by advising them they still needed to see for themselves to be successfully convinced to overcome the initial resistance. Based on their experiences, they relate that their outlook changed and they experience better outcomes.	***PatF7****: The clinician asked me the kind of bed I used… I told him it cannot be my bed, and all that but you know after the whole thing I was querying things, then I travelled for two weeks and I used another bed then I was able to conclude it was the bed.*<br><br>***PatF9****: I do exercise often but I stopped, I fear that what I am doing is even increasing the pain. I don't want to worsen the condition, and my husband told me to stop… but when I went to the clinic they did something similar and it subsided.*
**Strategies for implementation**<br>This highlights the participants' key ideas on strategies for the implementation of the stratified care approach.	Struggle against false information	Patients relate that there is a variety of information sources and many of which are misleading. These can come from other patients, from the internet or clinicians with certain motives. There is a constant struggle against wrong information by the patients which the therapists need to drive against to optimise care.	***PatM3****: Yes, they do mislead patients sometimes for financial implications, they may end up telling you to do something that is not necessary they can advise the patient to go for that treatment even though they know it might not help, so it requires a medical practitioner with integrity to be able to come out truthfully.*<br><br>***PatM2****: Yes, so be careful of the advice you get from clinicians because you might be getting it from quacks, make sure you get the advice from the right source.*

* Physiotherapists' and patient's quotes with pseudonymous designations comprising the numerical order of interviews.

For peer review only

**Category 7: Recognising the need for change**<br><br>**Themes of variation**	**Category Description: This category is relevant for physiotherapists and patients. It describes participants attention to deficiencies and deterrents in physiotherapists knowledge and practice, further corroborated by the patient's dissatisfaction with the current treatment outcomes which can be enabled by acceptance of the stratified care approach and proper funding.**		
	**Recognising the need for change**	**Explanation**	**Quotes***
**Tradition of treatment**<br>This describes components of the usual care, highlighting gaps in practice and aspects that can be modified to optimise care.	Lack of training to give psychologically informed therapy	Physiotherapists admitted having gaps in knowledge and lapses in training that might affect their ability to deliver optimal biopsychosocial care, this has resulted in sticking to the more familiar tradition of treatment. This issue of competency was a major setback. Physiotherapists felt incompetent to handle such an approach especially the prognostic approach and psychologically informed therapy.	***PTf4****: Most therapists do know about the prognosis of LBP condition, they need to be educated better on prognosis because you can't classify patients using this approach if you do not know'*<br><br>***PTf8****: Although general psychology was taught in school, this one relating to health care isn't taught. they do not know what exactly to do and how to do it. we just use the common-sense approach.*
**Evolution to a new system**<br>This discusses the key enablers needed to introduce a new practice aimed at optimising care.	Embracing a different approach	Physiotherapists and patients need to recognise the potential to optimise care to be open to accepting a different approach. The potential advantages need to be clear to participants creating an undeniable appeal of a different approach.	***PTm6****: A model like this … I feel if we embrace it one way or the other it would make a difference.*<br><br>***PTf4****: I think it will make life easier for patient and Physiotherapist if they can adopt it will go a long way to help,*
**Experiences**<br>This describes instances where attempts to install change have been made and the effects	No complete relief	Patients have undergone a series of treatments to change their condition but with no complete relieved some now seek solutions elsewhere. They express disappointment with their situation and frustration with the care they receive. They all feel that things need to change in the way physiotherapists treat patients.	***PatM4****: Drugs, physiotherapy, I am not completely relieved, I just got tired of going to the hospital. because I have been going for physiotherapy for the past 2-3 years*<br><br>***PatM8****: I thought by now I should have been fine…I feel the physiotherapy and medication wasn't doing anything to help my situation…I just felt I wasted my money and time,*
**Strategies for implementation**<br>This highlights the participants' key ideas on strategies for the implementation of the stratified care approach.	The role of funding	Participants complain of general issues relating to infrastructure, equipment and patient load. These issues are systemic constraints that are neither the fault of the patients nor the therapists. Participants jointly suggest improved funding to the health sector will improve services and aid implementation.	***PTm6****: Facility and equipment are some of the main issues. No physiotherapist would be willing to use his funds to go print questionnaires for patients, most of our offices do not have computers, and printers available.*<br><br>***PatM8****: Very poor, the infrared light they use on me everyone else has to wait until they are done with me, they have two and one is bad. So, if I needed it for ten mins it might be cut down to two or three minutes so that others can also use it.*

LBP: Low back pain

* Physiotherapists' and patient's quotes with pseudonymous designations comprising the numerical order of interviews.

For peer review only

Table 1 Consolidated criteria for reporting qualitative studies (COREQ): 32-item checklist

	No	Item	Guide questions/description
		**Domain 1: Research team and reflexivity**	
		Personal Characteristics	
Pg 5	1.	Interviewer/facilitator	Which author/s conducted the interview or focus group?
Pg 5	2.	Credentials	What were the researcher's credentials? *E.g. PhD, MD*
Pg 5	3.	Occupation	What was their occupation at the time of the study?
Pg 5	4.	Gender	Was the researcher male or female?
Pg 5	5.	Experience and training	What experience or training did the researcher have?
		Relationship with participants	
Pg 5	6.	Relationship established	Was a relationship established prior to study commencement?
Pg 5	7.	Participant knowledge of the interviewer	What did the participants know about the researcher? e.*g. personal goals, reasons for doing the research*
Pg 5	8.	Interviewer characteristics	What characteristics were reported about the interviewer/facilitator? e.g. *Bias, assumptions, reasons and interests in the research topic*
		**Domain 2: study design**	
		Theoretical framework	
Pg 4-5	9.	Methodological orientation and Theory	What methodological orientation was stated to underpin the study? *e.g. grounded theory, discourse analysis, ethnography, phenomenology, content analysis*
		Participant selection	
Pg 5	10.	Sampling	How were participants selected? *e.g. purposive, convenience, consecutive, snowball*
Pg 5	11.	Method of approach	How were participants approached? e.*g. face-to-face, telephone, mail, email*
Pg 8	12.	Sample size	How many participants were in the study?
Pg 8	13.	Non-participation	How many people refused to participate or dropped out? Reasons?
		Setting	
Pg 5	14.	Setting of data collection	Where was the data collected? e.*g. home, clinic, workplace*
Pg 5-6	15.	Presence of non-participants	Was anyone else present besides the participants and researchers?
Pg 8	16.	Description of sample	What are the important characteristics of the sample? *e.g. demographic data, date*
		Data collection	
Pg 6	17.	Interview guide	Were questions, prompts, guides provided by the authors? Was it pilot tested?
Pg 7	18.	Repeat interviews	Were repeat interviews carried out? If yes, how many?
Pg 5-6	19.	Audio/visual recording	Did the research use audio or visual recording to collect the data?
Pg 7	20.	Field notes	Were field notes made during and/or after the interview or focus group?
Pg 8	21.	Duration	What was the duration of the interviews or focus group?
Pg 5, 8	22.	Data saturation	Was data saturation discussed?
Pg 7	23.	Transcripts returned	Were transcripts returned to participants for comment and/or correction?
		**Domain 3: analysis and findings**z	
		Data analysis	
Pg 7	24.	Number of data coders	How many data coders coded the data?
Pg 7	25.	Description of the coding tree	Did authors provide a description of the coding tree?
Pg 7	26.	Derivation of themes	Were themes identified in advance or derived from the data?
Pg 7-8	27.	Software	What software, if applicable, was used to manage the data?
Pg 7	28.	Participant checking	Did participants provide feedback on the findings?
		Reporting	
Pg 10-17	29.	Quotations presented	Were participant quotations presented to illustrate the themes / findings? Was each quotation identified? e.*g. participant number*
Pg 17-19	30.	Data and findings consistent	Was there consistency between the data presented and the findings?
Pg 10	31.	Clarity of major themes	Were major themes clearly presented in the findings?
Pg 10	32.	Clarity of minor themes	Is there a description of diverse cases or discussion of minor themes?

(ii) Participant selection: Researchers should report how participants were selected. Usually purposive sampling is used which involves selecting participants who share particular characteristics and have the potential to provide rich, relevant and diverse data pertinent to the research question [13, 17]. Convenience sampling is less optimal because it may fail to capture important perspectives from difficult-to-reach people [16]. Rigorous attempts to recruit participants and reasons for non-participation should be stated to reduce the likelihood of making unsupported statements [18].

For peer review only - http://bmjopen.bmj.com/site/about/guidelines.xhtml

Downloaded from https://academic.oup.com/intqhc/article-abstract/19/6/349/1791966 by Fachhochschule Trier Hochschulbibliothek Standort Trier user on 22 August 2019

For peer review only

BMJ Open

Patients' and physiotherapists' perspectives of implementing a tailored stratified treatment approach for low back pain in Nigeria: a qualitative study.

Journal:	*BMJ Open*
Manuscript ID	bmjopen-2021-059736.R1
Article Type:	Original research
Date Submitted by the Author:	27-Apr-2022
Complete List of Authors:	Adje, Mishael; Trier University of Applied Sciences; Institute of Family Medicine, University of Lübeck, Germany<br>Steinhäuser, Jost; Institute of Family Medicine, University of Lübeck, Germany<br>Stevenson, Kay; The Medical School Keele University<br>Mbada, Chidozie Emmanuel; Obafemi Awolowo University, Department of Medical Rehabilitation<br>Karstens, Sven; Trier University of Applied Sciences, Therapeutic Sciences, Department of Computer Science
**Primary Subject Heading**:	Health services research
Secondary Subject Heading:	Health services research, Qualitative research
Keywords:	QUALITATIVE RESEARCH, Back pain < ORTHOPAEDIC & TRAUMA SURGERY, HEALTH SERVICES ADMINISTRATION & MANAGEMENT, Musculoskeletal disorders < ORTHOPAEDIC & TRAUMA SURGERY, PAIN MANAGEMENT

SCHOLARONE™ Manuscripts

I, the Submitting Author has the right to grant and does grant on behalf of all authors of the Work (as defined in the below author licence), an exclusive licence and/or a non-exclusive licence for contributions from authors who are: i) UK Crown employees; ii) where BMJ has agreed a CC-BY licence shall apply, and/or iii) in accordance with the terms applicable for US Federal Government officers or employees acting as part of their official duties; on a worldwide, perpetual, irrevocable, royalty-free basis to BMJ Publishing Group Ltd ("BMJ") its licensees and where the relevant Journal is co-owned by BMJ to the co-owners of the Journal, to publish the Work in this journal and any other BMJ products and to exploit all rights, as set out in our licence.

The Submitting Author accepts and understands that any supply made under these terms is made by BMJ to the Submitting Author unless you are acting as an employee on behalf of your employer or a postgraduate student of an affiliated institution which is paying any applicable article publishing charge ("APC") for Open Access articles. Where the Submitting Author wishes to make the Work available on an Open Access basis (and intends to pay the relevant APC), the terms of reuse of such Open Access shall be governed by a Creative Commons licence – details of these licences and which Creative Commons licence will apply to this Work are set out in our licence referred to above.

Other than as permitted in any relevant BMJ Author's Self Archiving Policies, I confirm this Work has not been accepted for publication elsewhere, is not being considered for publication elsewhere and does not duplicate material already published. I confirm all authors consent to publication of this Work and authorise the granting of this licence.

For peer review only

Patients' and physiotherapists' perspectives of implementing a tailored stratified treatment approach for low back pain in Nigeria: a qualitative study.

Adje Mishael[1,2], Jost Steinhäuser[2], Kay Stevenson[3], Chidozie Mbada[4], Sven Karstens[5]

[1]Trier University of Applied Sciences, Trier, Germany. [2] Institute of Family Medicine, University of Luebeck, Germany, [3]The Impact Accelerator Unit, The Medical School Keele University, Keele, UK, [4] Department of Medical Rehabilitation, Obafemi Awolowo University, Ile-Ife, Nigeria, [5]Therapeutic Sciences, Department of Computer Science, Trier University of Applied Sciences, Trier, Germany.

Correspondence to Mishael Adje; adjem@hochschule-trier.de.

Abstract

Background: Stratified care has the potential to be efficient in addressing the physical and psychosocial components of low back pain (LBP) and optimise treatment outcomes essential in low-income countries. This study was aimed to investigate the perceptions of physiotherapists and patients in Nigeria towards stratified care for the treatment of LBP, exploring barriers and enablers to implementation.

Methods: A qualitative design with semi-structured individual telephone interviews for physiotherapists and patients with LBP comprising research evidence and information on stratified care was adopted. Preceding the interviews, patients completed the STarT-Back Tool. The interviews were recorded, transcribed and analysed following grounded theory methodology.

Results: Twelve physiotherapists and thirteen patients with LBP participated in the study (11 female, mean age 42.8 (SD 11.47) years). Seven key categories emerged: *recognising the need for change, acceptance of innovation, resistance to change, adapting practice, patients learning journey, trusting the therapist and needing conviction.* Physiotherapists perceived stratified care to be a familiar approach based on their background training. The prevalent treatment tradition and the patient expectations were seen as major barriers to implementation of stratified care by the physiotherapists. Patients see themselves as more informed than therapists realise, yet they need conviction through communication and education to co-operate with their therapist using this approach. Viable facilitators were also identified as patients trust in the physiotherapist and adaptations in terms of training and modification of the approach to enhance its use.

Conclusion: Key barriers identified are the patient's treatment expectations and physiotherapists' adherence to the tradition of practice. Physiotherapists might facilitate implementation of the stratified care by communication, hierarchical implementation and utilisation of patients' trust. Possibilities to develop a consensus on key strategies to overcome barriers and on utilization of facilitators should be tested in future research.

Keywords: Qualitative research, Low back pain, Physiotherapy, Grounded theory, Stratified Care

For peer review only

Strengths and limitations of this study

- The barriers and enablers to implementation of stratified care in low income settings like Nigeria is explored from the perspective of patients and physiotherapists in this study.
- This study adhered to a rigorous circular grounded theory method to systematically draw out comparisons and variations in perspectives of 25 participants until saturation was reached.
- Participants were motivated to engage in productive discussions and involved in co-designing introductory materials and tailoring interview guides.
- The researchers' own subjectivities, beliefs and experiences might have played a role in shaping the outcome, but this can be seen as an inevitable and integral part of using grounded theory.
- The need for stakeholder awareness in areas of divergent perspectives, to inform quality communication and patient conviction seen in this study is in concordance with current evidence on stratified care implementation in other contexts.

For peer review only

Introduction

Low back pain (LBP) is among the most frequent reasons for visits to outpatient physiotherapy clinics in Nigeria (1–3). It is a prominent cause of disability and the effects have been immense in terms of cost, time and productivity loss (4–7).

In recent decades, there has been remodelling in the management of LBP. Current literature corroborated by multiple guidelines recommend the biopsychosocial model of care (8–13). These resources highlight the prognostic significance of psychosocial factors such as depression, fear-avoidance behaviours, catastrophising, distress among many in individual patients and their culpability in worsening musculoskeletal conditions (14, 13).

Stratified care encompasses specific concepts in the management of LBP, differentiating and targeting prognostic subgroups, aligning the risk of an unfavourable treatment outcome with specific evidence-based treatment procedures (15). Its principles lean towards prognosis, de-emphasising pain, disputing unhelpful beliefs and equipping patients on self-management strategies (13, 8, 9). It aims to guide clinicians to identify psychosocial risk factors hindering patient recovery and fast track accurate treatment decisions (16, 17).

One well-known stratification approach with demonstrated effectiveness in both clinical and cost outcomes is the Subgroups for Targeted Treatment (STarT-Back) approach (18, 19). It involves categorising LBP patients into low, medium and high risk categories using the STarT-Back tool (SBT) and allocating matched treatments to participants in each category (20).

The SBT was developed for primary health care settings, it is a validated tool that can be administered paper-based or online-based. It consists of 9-items, designed to assess modifiable risk factors (20). The first four questions address aspects of the patient's physical characteristics asking about the spread of the pain towards the leg, to the neck or shoulders, and disability in patients' dressing or walking. The next five questions comprise the psychosocial subscale relating to patients' psychological characteristics, including fear of movement, anxiety, catastrophising, depression and bothersomeness (20).

In a large scale study evaluating the effectiveness of the STarT-Back approach, the score from the SBT was used to classify patients into three subgroups (19). Depending on the subgroup, there are treatment recommendations. If a patient scores between 0 and 3 in total on the SBT, they are allocated to the low-risk subgroup. These comprise patients who will improve with little or no intervention. They are helped with advice and pain medication. If patients score between 4 and 9 on the grouping tool but have 3 or fewer of the five sub-scale, they are allocated to the medium-risk subgroup. These have a moderate likelihood of poor treatment outcomes, they have lesser physical and psychological components of pain. The emphasis here is on physiotherapy and self-management (21). Patients who score 4 of 5 on the psychological subscale are allocated to the high-risk subgroup. These patients possess complex

For peer review only

prognostic factors impeding recovery and need more care due to these. Such care comprises psychologically informed physiotherapy, aimed to resolve physical and psychosocial components of pain (15, 19). All target treatments are aimed to reduce disability, reduce pain where possible, improve psychological functioning and enable the patient to manage ongoing and/or future episodes of back pain (21).

Studies from the UK and New Zealand reveal that the implementation of the STarT-Back model of care has the potential to reduce disability, save costs, improve patient satisfaction, reduce the number of patient visits and increase clinician's competence (19, 22, 23) except for a US study indicating no effect on patient outcomes (24). A recent systematic review shows that this approach provides benefits in terms of clinical outcomes and health-related cost savings for all three subgroups of patients and patients in the high-risk subgroup who received psychologically informed treatment had significant improvements in pain, disability, depression and general health (25). The SB approach is described as best practice in the UK international guideline for the management of LBP (26, 11) and has been recommended from studies in the UK and other countries to be efficacious and have a positive impact on LBP outcomes (18, 21, 19). However, these are mostly high-income countries (UK, Denmark) with financially sound health care systems, further care is taken to ensure proper implementation in countries in Africa

Studies on implementation efforts in higher-income countries exhibit challenges further amplified when considering contextual factors present in lower/middle-income countries (18, 19, 15). A study shows, that socio-economic status is an important treatment effect modifier when using the SB approach (27). Differences in settings can also play a major role in determining the outcome of implementation (24). Unique circumstances make it challenging for some physiotherapists to consider practice guideline recommendations when planning treatment (28). This leads to the omission of psychosocial risk factors, which undermines treatment outcome (29) and is present in over 60% of patients with chronic LBP in Nigeria (30). In combating this, implementation of the STarT-Back model of care promises a viable solution. Since there is no universal instruction manual for implementation, it is recommended that stratified care for LBP will first need adaptation in terms of setting before wider implementation is considered. The first step to this is identifying the contextual barriers and enablers to implementation (15).

This study was aimed to explore the perspectives of physiotherapists and patients with LBP, identifying perceived barriers and enablers to the implementation of stratified care in Nigeria.

Methods

A qualitative design was adopted in this study. Semi-structured individual telephone interviews for patients and physiotherapists were extensively executed following grounded theory methodology (31,

For peer review only - http://bmjopen.bmj.com/site/about/guidelines.xhtml

32). The Consolidated Criteria for Reporting Qualitative Research (COREQ) was employed for the study report (33).

Interviewer/Facilitator

The research team was made up of five researchers: MA (male), a Nigerian physiotherapist with experience in musculoskeletal health; SK (male), a physiotherapist with experience in qualitative and health services research; JS (male), a general practitioner with experience in medicine and qualitative research; KS (female), a physiotherapist with experience in health promotion and implementation of the STarT-Back approach; and CM (male) a physiotherapist with experience in musculoskeletal research in Nigeria. The interview guidelines were prepared and reviewed by MA, SK, JS and the interviews were conducted by MA having no direct relationship with the participants (34).

Participant recruitment

Participants were theoretically sampled for the interview rounds (35, 32). Participants were contacted following set inclusion criteria specifically comprising physiotherapists and patients living in Nigeria.

To be included in this study, physiotherapy participants had to be licensed and registered by the regulating body (Medical Rehabilitation Therapist Board) of Nigeria and practising physiotherapy in Nigeria with entry-level qualification and above. They should have worked on the STarT-Back information on the introductory video provided. Patients participants had to be diagnosed as having non-specific low back pain by their physician or physiotherapist and have visited a health care institution for physiotherapy care. They should speak and understand the English language fluently and should be able to fill the STarT-Back questionnaire. All participants must be above 18 years.

Patients with a diagnosis or having any signs or symptoms pointing to a severe disease condition (e.g. cancer) as the cause of their low back pain were excluded from this study.

Physiotherapist participants were from diverse specialities in diverse work settings, with varying work experience and levels of training. Patients were with LBP of varying severity and any episodic duration spread across the three risk subgroups in the STarT-Back classification and receiving treatment from any health institution. These were approached and invited by the physiotherapists who have agreed to participate (36).

Sociodemographic details comprising age, sex, work status were obtained from participants. The STarT-Back risk categories were determined. Physiotherapy participants provided details on age, sex, previous training, working experience (overall/with LBP-patients), qualification and work-setting. Participants were recruited until saturation was reached (37).

For the introduction of the concept of stratified care to patients and physiotherapists, interactive videos were prepared and presented before the interviews (19). The videos were designed with a voice layover and interactive questions embedded within to keep participants involved and to help them understand

For peer review only

key aspects. These videos were prepared following the guidelines for instructional videos by Norman (2017) (38) with input from the STarT-Back Tool development group in the UK. The content was developed for two specialized videos for the participating physiotherapist and one video for the patient with underlying principles from Main et al. (2012) (39) as shown in Table 1. Patient video was co-produced with patient input, using lay understandable language and physiotherapists videos were co-created with input from physiotherapists. Stakeholder input informed the content, timing, use of language and length of presentation (40).

Data Collection

Interviews were conducted over the phone at the convenience of the participants.

Table 1: Content of the introductory presentation adapted from Main et al. (2012).

For patients	For physiotherapists
The rationale for using SB approach, including potential clinical benefits	The rationale for SB approach
Psychosocial components of LBP	Psychosocial barriers to progress
Description of the SB approach	Grouping using the SB Tool
- Content and purpose	- Content and purpose
- Definition of subgroups	- Definition of subgroups
- Matched treatments	How to use and score the tool
Treatment outcome using the approach	Scientific underpinning of the approach
	Clinical and economic benefits

SB: STarT-Back, LBP: Low Back Pain

The interview guides for patients and physiotherapists were prepared in four phases; brainstorming, collection, sorting, examining questions and consultations as described in research (41, 42). In developing the initial interview questions, the aspect of implementation was duly considered(43).

Patient guideline: Developed questions were re-phrased to simpler forms for patients and to reveal the patient's perspective (online supplemental table 1). Then sorted repeatedly based on categories (44), allowing for open but thematically structured interview questions (45). They were then reviewed by a patient with LBP and modified by inputs (40). Four key questions were developed with additional maintenance questions and follow-up questions for patients with LBP.

Physiotherapist guideline: The questions for physiotherapists were re-phrased to reflect physiotherapists' perspectives (online supplemental table 2). Two physiotherapists with experience in qualitative interviewing reviewed the questions and made inputs. These were adopted into the guideline ready for use. Four key questions were developed with additional maintenance questions and follow-up questions for physiotherapists.

For peer review only

A pilot test of the interview guidelines was carried out on a sub-sample of the target population (n=5) (3 physiotherapists and 2 patients with LBP). Verbalisation feedback by participants informed the modification of the interview questions and approach strategy, as exemplified in a study by Pepper et al. (2018) (46). The focus of the interviews was hence modified simultaneously with theoretical sampling (40).

The telephone interviews were conducted by MA following the pattern of a problem-centred interview (45). The interviews were tape-recorded and handwritten notes were taken during the interviews. Verbal consent was received for each participant before commencement. Questions were flexibly rearranged to express a dialogue and improve the flow of the interview, beginning with warm-up questions. These questions drew insight into the expert's background to create a context and relaxing warm-up atmosphere.

Notes were summarised back to the participants after each interview and memos were written about each interview. This process was repeated for the various rounds until saturation, where no new categories emerged (37).

Data Analysis

A simple transcription method as described by Dresing and Pehl (2012) was used (47) with the help of Easy transcript software (48). Pseudonyms were used in each transcript making it recognisable during the coding process and differences between patient and physiotherapist. Transcripts were plain text only to facilitate the coding process and were read separately by a second researcher with experience in qualitative analysis.

Coding

Transcripts were read and re-read by MA and SK. A detailed coding agenda was developed with code and category descriptions to guide the coding process (online supplemental table 3) and distributed to all authors for feedback. Thereafter, categories were identified inductively.

Code labels were lifted directly from participants quotes identifying descriptive categories first (32). Higher-level abstract categories were systematically identified separately by the MA and SK as coding progressed, tested against the data and consolidated repeatedly. Variations among categories were derived as recommended by Sbaraini et al. (2011) (49). The data was broken down, intrinsically compared to explain variations and re-combined in an abstract manner describing the relationships.

The line-by-line open coding method was used while the RQDA package in the R software was used for the coding process (50).

For peer review only

Ethical approval

Ethical approval was obtained from the Trier University of Applied Sciences and the Obafemi Awolowo University Teaching Hospital, Ile-Ife Nigeria (registration ID: IRB/IEC/0004553). Approval for telephone interviews was also obtained from the Trier University of Applied Sciences. All participants were sent information sheets and provided written informed consent before participation.

Patient and public involvement

The circulatory process of qualitative research using grounded theory ensured that patients and physiotherapists as public partners played a key role in theoretical sampling and developing the interview guideline (40). The use of the STarT-Back tool was informed by collaborative efforts and consultations of research team members including KS working intensively with patient consultants.

Specifically, patients were consulted in development and tailoring of the information videos and two patients were part of the pilot team for the interview guidelines. They informed the burden of information, time allocated for participation, direction of further enquiry under the grounded theory methodology. Patients contributions and referrals contributed to the ideas guiding further recruitment. Patients and physiotherapists as public partners helped in the tailoring of the interview questions comprising key questions, maintenance and follow up questions. This included adapting wording, and sentence structures and including simple patient specific information. After the interviews, summary notes were read back to patients for confirmation and they gave feedback on the interviews.

Results

A total of 33 participants were contacted and at different stages by email, and 25 consented to participate. Of the eight remaining participants, two patients were uninterested, while six physiotherapists could not find the time for the study. This resulted in a response rate of 81% for patients and 70% for physiotherapists. All consented participants were interviewed, 14 male and 11 female (12 physiotherapists and 13 patients), lasting an average of 50 minutes per individual. Physiotherapists had backgrounds in various specialisations and represented a variety of work settings. Patient participants were classified as 'high risk, medium and low' based on the STarT-Back tool.

Tables 2 and 3 show detailed information on participants' practice experience, experience with LBP, qualification, speciality, work setting and STarT-Back classification.

For peer review only

Table 2: Patients sample description

Set[a]	Title[b]	Age group (y)	Sex	Work status	STarT-Back Classification[c]
1	Pat1	>50-60	M	Paid work	High risk
2	Pat2	>50-60	M	Paid work	Low risk
	Pat3	>40-50	M	Paid work	High risk
	Pat4	>70-80	M	Retired	High risk
	Pat5	>60-70	M	Retired	Low risk
3	Pat6	>60-70	F	Self-employed	Medium risk
	Pat7	>30-40	F	Self-employed	Medium risk
	Pat8	>40-50	M	Paid work	High risk
	Pat9	>40-50	F	Self-employed	High risk
	Pat10	>50-60	F	Self-employed	Medium risk
4	Pat11	>30-40	F	Paid work	High risk
	Pat12	>40-50	M	Paid work	Medium risk
	Pat13	>30-40	M	Self-employed	High risk

Pat: Patient

[a] First, second, third and fourth iterative interview rounds.

[b] Patients' pseudonymous designation comprising the numerical order of interviews used to reference patients' quotes.

[c] Based on the STarT-Back Tool. Patients who score 0-3 were allocated to the low-risk subgroup, 4-9 but 3 or fewer of the five sub-scale are medium risk, 4 of 5 on the psychological subscale are high risk. Higher scores indicate an increasing complexity of the condition.

For peer review only

Table 3: Physiotherapists sample description

Set[a]	Title[b]	Age group (y)	Sex	Practice	Experience with LBP	Level of education	Area of interest	Work Setting
1	PT1	>30-40	M	>10-20	>10-20	MSc	Orthopaedics, Private Practice	Physiotherapy Training Institute
	PT2	>30-40	M	>0-10	>0-10	BSc	Orthopaedics, Private Practice	Primary Health
	PT3	>20-30	F	>0-10	>0-10	BSc	Women's Health	Private Practitioner
	PT4	>30-40	M	>0-10	>0-10	PhD	Orthopaedics, Educator	Physiotherapy Training Institute
2	PT5	>30-40	M	>0-10	>0-10	BSc	Orthopaedics, Paediatrics	Teaching Hospital
	PT6	>30-40	M	>0-10	>0-10	BSc	General Practice	Specialist Hospital
	PT7	>30-40	F	>0-10	>0-10	BSc	Neurology, Paediatrics, Geriatrics	Teaching Hospital
3	PT8	>30-40	F	>10-20	>10-20	BSc	CRP, Orthopaedics	Teaching Hospital
	PT9	>30-40	M	>0-10	>0-10	BSc	Paediatrics	Teaching Hospital
	PT10	>20-30	F	>0-10	>0-10	MSc	Ergonomics, Occupational	Corporate Organisation
4	PT11	>30-40	F	>0-10	>0-10	BSc	CRP	Teaching Hospital
	PT12	>30-40	F	>10-20	>10-20	MSc	Orthopaedics	Physiotherapy Training Institute

CRP: Cardiorespiratory physiotherapist, BSc: Bachelor of Science; MSc: Master of Science; PhD: Doctor of Philosophy. PT: Physiotherapist,

[a] first, second, third and fourth iterative interview rounds.

[b] Physiotherapists' pseudonymous designation comprising the numerical order of interviews used to reference physiotherapists' quotes.

The perspectives of physiotherapists and patients to the implementation of a tailored stratified care approach were captured in seven main hierarchical categories:1) recognising the need for change, 2) acceptance of innovation, 3) resistance to change, 4) adapting practice, 5) patients learning journey, 6) trusting the therapist and 7) needing conviction (Table 3). These categories varied based on four themes: a) tradition of treatment, b) evolution to a new system, c) experiences, d) strategies for implementation (Table 4). While some categories focused only on physiotherapists others focused only on patients and still others on both physiotherapists and patients.

For peer review only

Table 4: Categories and Themes of variation

Categories	Themes of variation<br>a)<br>Tradition of treatment	b)<br>Evolution to a new system	c)<br>Experiences	d)<br>Strategies for implementation
**1) Resistance to change**	Incentives attached to the usual practice	Overcoming patients' expectations	Organisational culture	Targeting attitudinal change
**2) Acceptance of innovation**	Ease of transition	Open to new knowledge	Steps towards optimising practice	Work Settings
**3) Adapting practice**	Need of standard for regulating the practice	Cultural adaptations	Use of communication	Awareness for patients and PT
**4) Patient's Learning Journey**	Needing a complement to usual care	Recognising unhelpful treatments	Learning to live with pain	Taking charge
**5) Trusting the PT**	Getting some help	Learning with practice	Therapists doing their best	Co-operating with the PT
**6) Needing conviction**	Reliance on investigations	Patient education	Self-discovery	Struggle against false information
**7) Recognising the need for change**	Lack of training to give psychologically informed therapy	Embracing a different approach	No complete relief	The role of funding

For peer review only

Resistance to Change

This is a mixed category as it contains aspects focusing on patients and physiotherapists. It describes the resistance perceived and experienced by physiotherapists and patients concerning the implementation of the stratified care approach. It further describes the physiotherapists' and patients' perception of challenges to the fundamental change and strategies to overcome resistance.

Participants opined that physiotherapists' *tradition of treatment* was a major challenge, it seemed to be an overwhelming issue sustained by *incentives attached to the usual practice*. Key aspects are ego, self-confidence in their current practice experience and finance. There is a long-held tradition of treatment with non-evidence-based methods, diagnosis and radiographs that the patient has got used to and this tradition remains deeply entrenched in practice.

> *PTm3: You need more patients to have more income, so the more the patients the more the income, so that is the case.*

The variation *strategies for implementation* indicates *targeting the attitude of physiotherapists and patients* which is seen by participants as a major strategy to improve the chances of successful implementation. The physiotherapist and patients see themselves as mostly complacent. The patients

blamed the physiotherapists for not being attentive to their needs and the physiotherapists blamed the patient's attitude as a deterrent for being unable to improve their condition.

> *PatM3: I know patient attitude matters…but at least play your role and if the patient misbehaves you know your job is done. If your recommendations are practicable you will get a good attitude from them.*

Participants' perceptions varied in relation to an *evolution to a new system* as therapists see *patient expectation as* a primary deterrent to implementing stratified care that needs to be addressed. It feels to them like all their suggestions and efforts to optimise practice will not work because the patients come with a certain expectation. They expect a strong touch, massage or something physical that would bring the pain down to zero. There is, however, an enabling situation seen by therapists and patients that these expectations can be adjusted and overcome.

> *PTf4: Then for the patient, … they might not be satisfied because they are expecting more from you'…if you don't explain why you have to do that and you just do it they won't feel happy. They feel they need some form of hard touch, strong touch.*

From the experiences of physiotherapists and patients, the preponderant *organisational culture* makes treatment less efficient and less effective. They have limitations in their ability to ease the changes beyond a certain point, like organising situations, follow-ups or influencing patients' working conditions due to a systemic resistance.

> *PTm6: An example is the government trauma centre I worked in, they had made a move to implement an electronic documentation system for use, but eventually, it never saw the light of day, because the attitude towards it was terrible from the management and recipients.*

Acceptance of Innovation

This is a category focusing on physiotherapists. It describes a positive reaction from physiotherapists to the idea of implementation of stratified care, highlighting their perspectives on the best settings and enabling conditions for implementation with inputs from patients.

Some physiotherapists reported similarity of stratified care to what they already know comprising the usual *tradition of treatment* and practice in their management of LBP, they have background knowledge of the biopsychosocial approach and various treatments for patients. These are individual aspects of stratified care and participants reported that this *eases the transition* and aids them to adopt the stratified care in its entirety.

> *PTm5: I won't say the knowledge of psychosocial problems associated with LBP is low among physiotherapists any longer. I think there has been a lot of awareness and people are getting to know about those issues. We have been always talking of psychosocial aspects and the likes. I think this brings it more to the fore...I think using this approach helps… it's going to be easier for them to incorporate them into routine practice.*

For peer review only - http://bmjopen.bmj.com/site/about/guidelines.xhtml

Physiotherapists also describe an *evolution to a new system* comprising some form of realisation, that this approach is fundamentally new, uncommon and not used in its entity in clinical practice, hence they *were open to new knowledge* describing stratified care as an improvement to current care and welcome development.

> *PTf4: It is very different from what we do here, the treatment here is based on diagnosis, this approach is based on prognosis so yeah, it is different.*

From their *experiences*, participants came across situations where they made *steps towards optimising practice*, instances where innovations, ideas or approaches have been introduced in their various work settings, the process that helped and the positive responses from colleagues. Key aspects were the use of clinical discussions, through physiotherapists' cadre and time-saving measures for the patient. They think the stratified care being introduced in a similar stepwise manner will be successful.

> *PTf11: Many have been introduced to us by different people, I have even tried to introduce one and it was not a big deal if you have a proper literature backup. once it is adopted everyone used it, for instance, when Kinesio-taping was introduced to us, the same thing happened. it was accepted and then everyone practised using it.*

Participants expressed differing perspectives on *strategies for implementation* of stratified care in relation to the most viable *work setting* to target since there exist different situations and ideologies at the private and government establishments. Participants felt it would be more applicable if targeted at the government hospitals since the therapists are paid by the government a fixed amount and more treatments do not lead to more money.

> *PTf10: Then for physiotherapists, working in government hospitals they might be encouraged to use the approach, but not therapists in the private sector… this approach will reduce their revenue and will not be welcomed.*

Adapting Practice

This category applies to patients and physiotherapists. It describes the suggestions on adaptations in terms of creating awareness and modification of the approach to suit the Nigerian context and enhance its use. Here, participants highlight their opinions and experiences on ways to adapt the stratified care approach.

Participants speak of *Socio-cultural adaptations* relating to language, religion, the culture of respect and hierarchical implementation from the seniors to juniors. This was highlighted as a viable means of adapting practice at the introductory phases in attempting the evolution to a new system.

> *PTm9: Our environment is peculiar things have to go through certain paths, we need to convince the seniors. then it can be implemented on a departmental level. It is practically impossible for one person to champion it.*

For peer review only - http://bmjopen.bmj.com/site/about/guidelines.xhtml

Most participants mentioned that the *use of communication* was a major way to inform adaptation and improve the patient-therapist relationship. From their *experiences*, when communication between patient and physiotherapist was used, it was seen to produce positive effects.

> *PatF7: In a way, some part of my treatment helped me psychologically, there are days he would talk to me so I can help myself.*

Participants argued that the absence of a standard of practice embedded in their usual *tradition of treatment* and tailored to the needs of Nigerians was a major gap in practice. This situation led to a clamouring for *standards to guide practice and regulation.* Adherence to such a standard of practice would enhance adaptation for implementation.

> *PTf7: In Nigeria, we do not have a standard way of doing things. what hospital A is doing is different from what Hospital B is doing… everyone is just doing what they want.*

Participants confirmed that creating *awareness for patients and physiotherapists* about the approach is a major *strategy for implementation*. While some opined this can be done at undergraduate and post-graduate levels for physiotherapists, patient's awareness was achievable through outreaches.

> *PTf4: Patients…should know that physiotherapy involves advice too and they will feel better. it would also help physiotherapists a lot if they are aware of the approach.*

Patients Learning Journey

This category focuses on patients and physiotherapists. Here, patients have experienced care from various fronts and have formed their opinions about what they have experienced to be helpful to their condition. This was corroborated by the physiotherapist opinions about the patients' learning experiences from their perspective.

Patients here describe having undergone a learning journey, they have gathered added experiences. Participants explain how the debilitating condition of the patient's experiences has resulted in personal difficulties. They resolved in their minds to change their outlook, *learn to live with pain* and accept the condition, and this has improved their outlook.

> *PatM3: It affected me psychologically, I was worrying but I have learned to live with it, I think I am better at managing myself …so also, I have learned, I have concluded that no kind of intrusive treatment can work for this my case, that I just need to be careful with how I live my life.*

For patients, the usual *tradition of treatment* care was seen to be insufficient and there are aspects that patients feel would *need some form of compliment,* they feel this can be achieved by treatment from traditional bone setters which is seen as a compliment or an alternative.

For peer review only

> *PatF11: It is not everything science can explain…as some hospitals do not have machines, these strong (traditional) hands can be used instead…so some things the hospital cannot handle, these (bone setters) know where and where to touch.*

Participants report that over the past recent years, patients have gradually become mentally equipped to *recognise unhelpful treatments* by themselves and developing the ability to differentiate between what is beneficial and what isn't. This realisation is seen as a major stride in the *evolution to a new system* and a viable enabler to the implementation of stratified care.

> *PTf8: When they visit these, they come back (from traditional bone setters' treatments) with complications that I have to deal with so it is a barrier, and over the years it was more, but recently the percentage is coming down now it should be about 30 percent but back then some years ago it was about 70 percent. it makes their treatment longer.*

Patients mentioned that they have gathered knowledge of self from various treatment exposures, from self-care, traditional bone setters' treatments. They have now decided that *taking charge* of their treatment is a vital *strategy for implementation* and ultimately a strategy to help themselves.

> *PatM5: My advice is that people should exercise and remain active. I know the positive side of exercise, it is extremely good. That is why I don't miss it at all, no matter what happens.*

Trusting the Therapist

This category focuses on patients. It describes the patient's views of the physiotherapist as a dynamic expert with whom they need to give their trust and co-operation based on the efforts of the physiotherapist to help their situation and their experiences with treatments.

Patients agreed that some aspects of the usual *tradition of treatment* especially those focused on exercise and advice provided some relief to patients, they reported they *got some help* from it. Even though not permanent they relate some improvement in their condition and trust that based on this result more can be expected.

> *PatF9: Just the exercise he prescribed, stretching on the bed, but as I was doing it initially I was feeling the pain much but later it reduced, formerly I counted just 20 counts, but now I can count up to 40 without feeling much pain, the physiotherapist gave me the exercise.*

Most patients expressed that the physiotherapists will gradually *evolve to the new system* in their practice and that some therapists are already on the right track, *learning to change their approach with practice*. They feel exploring ideas like this through research is needed to evolve more.

> *PatM5: This can be the case sometimes, in a bid to solve a problem they solve others. That's how they learn, they learn by practice. Experience is the best teacher, so most times experience comes into play.*

For peer review only - http://bmjopen.bmj.com/site/about/guidelines.xhtml

The patients acknowledge that the *physiotherapists are doing their best* by putting in efforts to optimise care in ways they can and they appreciate the efforts, they notice from their personal *experiences* that when some more is done to help their condition it encourages them to focus more on the efforts.

> *PatM4: Yes, for me, they did the best they could I would say, for the treatment too… they used to tell us and give us hope, the possibility to get better. they have human sympathy, they advise us sometimes that helps.*

The patients consider the physiotherapist's role as providing care and support for the patient when needed and their role as patients is not to interfere with the process. As a *strategy for implementation,* they felt they need to *co-operate with the physiotherapist* but largely leaving the aspect of care for the therapists.

> *PatF1: When you know this is what your clinician wants to achieve then anything he tells you would do it, do it so that you can get a good result because when you do not follow up well you might think it did not work because didn't follow instructions well, so if you co-operate you would be able to achieve what you want.*

Needing Conviction

This category focuses on patients. It describes the patient's exposure to false information sources and the need for physiotherapists to provide trustworthy education to get their full cooperation. This equips patients on their road to self-discovery and guides them to conviction.

Participants argue that for patients to be able to accept change, they need to be informed properly to a level of conviction. Patients have multiple sources of information and this needs to be presented clearly and reasonably to influencing attitudes and lifestyle. If *patients can be educated* and the procedures explained properly, it will go a long way to influence their expectation, help them become more receptive to a change in approach and a viable enabler in the *evolution to a new system.*

> *PatM2: The patient needs such conviction, he should not leave with the feeling that 'that clinician does not have my interest and doesn't want to treat me let me not waste my time to go back there…. he has to be convinced he is not just assigned to that group because you feel that his problem is not serious enough to be attended to.*

Physiotherapists agree that the *tradition of treatment* they are already used to involves *reliance on investigations*, a diagnosis-based approach, using radiographs and passive treatments. Since the patients have become used to that, they need some form of conviction to accept the change. Some patients enjoy passive treatments and would not only prefer that but insist it is the right thing to do.

> *PatM3: The only decision you make that will make sense to me is based on evidence from X-rays, tests and the likes not just based on your opinion.*

Patients relate situations when they needed to be convinced from within through some form of *self-discovery*, even though their therapist tried by advising them they still needed to see for themselves to

For peer review only

be successfully convinced to overcome the initial resistance. Based on their *experiences*, they relate that their outlook changed and they experience better outcomes.

> *PatM5: They told me to check my body mass index, but I told them it might not be right, for the past 30 years I have never gone below 80 kilograms, so going to that weight I don't know how I would look like. It would be terrible…I did physiotherapy, took medications and went to hospitals several times. Until I discovered that my weight was the cause… I can manage myself now.*

Patients emphasise that there is a variety of information sources and many of which are misleading. These can come from other patients, from the internet or clinicians with certain motives. There is a constant *struggle against wrong information* by the patients which the therapists need to drive against to optimise care. To combat this, information pools, research banks were suggested as viable *strategies for implementation.*

> *PatM5: Yes, although if it is the general internet sometimes it gives false information too. Information pool is needed, as a research bank for publications available to all clinicians so that anyone who needs it can go into such pool in managing their patient.*

Recognising the Need for Change

This category focuses on physiotherapists and patients. It describes participants' attention to deficiencies and deterrents in physiotherapists' knowledge and practice, further corroborated by the patient's dissatisfaction with the current treatment outcomes which can be enabled by acceptance of the stratified care approach and proper funding.

Physiotherapists admitted to having gaps in knowledge and *lapses in training* that might affect their ability to deliver optimal biopsychosocial care, this has resulted in sticking to the more familiar *tradition of treatment. This* issue of competency was a major setback. Most physiotherapists felt incompetent to handle such an approach especially the prognostic outlook and psychologically informed therapy.

> *PTf10: Physiotherapists in Nigeria do not have the training to give psychologically informed therapy, our training does not cover that, except we just use our instinct, most times what physiotherapists do in the name of psychological therapy is just talk and say some things through instinct.*

Many patients have *experienced* treatments involving significant efforts with sub-optimal results. Some now seek solutions elsewhere because there has been *no complete relief.* They express disappointment with their situation and frustration with the care they receive. They all feel that things need to change in the way physiotherapists treat patients.

> *PatM3: I had received treatment for some years until 2016, no relief after physiotherapy and I was contemplating travelling abroad for treatment. Clinicians treating patients need the training to update their treatment methods.*

Participants complain of issues of infrastructure and patient load. These issues are systemic constraints that are neither the fault of the patients nor the therapists and have a common underlying denominator

For peer review only

as a lack of proper funding. Participants jointly suggest improved *funding* to the health sector as a sound *strategy for implementation* aimed at improving services.

> *PatM5: There is a proverb in my place 'good soup cost good money'. so, money plays a bigger role in this. It needs to be available for any of these to work.*

Physiotherapists and patients agreed they need to recognise the potential to optimise care to be open *to accepting a different approach*. The potential advantages need to be clear to participants, creating an undeniable appeal of a different approach. This is seen as a facilitator for the *evolution of the new system.*

> *PatM3: Well, I think it is a new approach. It is good, you can then know the kind of treatment and the extent to which you should offer treatment. I believe that this approach goes a long way into what results. It plays a big role.*

Discussion

Exploring the perspectives of physiotherapists and patients with LBP in this study has produced insight into the varied barriers and enablers affecting the implementation of stratified care in Nigeria. Findings from this study show that some physiotherapists described stratified care as a familiar approach to be welcomed. This is consistent with previous knowledge, Sanders et al. (2014) reported thoughtful obedience from physiotherapists (51) and Odole et al. (2020) wrote about the willingness of physiotherapists to adopt innovative care methods (52). A reason for this could be having previous knowledge from a standard entry-level training that encompasses basic components of psychosocial care and evidence-based practice with an emphasis on communication as described by Abaraogu et al. (2019) (53). Other studies show physiotherapists had previous knowledge of the biopsychosocial approach (54, 55). This shows the viability of adopting a stratified care approach and ease of transition among physiotherapists.

However, for implementation, the physiotherapists in our study described more challenges with implementing the approach than the patients. Two possibilities could explain this; a negative attitude towards change, as studies reported, clinicians do not always deliver therapy according to the standard they received during training (56, 57, 28). A second possibility could be that they understand the intricacies of use better than the patients (15). Research shows that an understanding of the complexities involved in an intervention creates in itself a capacity for implementation (58, 43) Thus, physiotherapists especially expressed concern on internal and external resistance they face in optimising practice including patient's expectations, patient's attitude, patient-load-to-physiotherapist-ratio and socio-economic constraints consistent with findings on the implementation of the stratified care in other contexts (59–61).

For peer review only - http://bmjopen.bmj.com/site/about/guidelines.xhtml

Despite these perceived barriers, from the interviews, some physiotherapists consider the current practice tradition to be outdated, but this treatment tradition is further enabled by their perception of patients' expectations and the physiotherapist's attitude as well. They are concerned that patients may be unsatisfied with their treatments if they change and thus be swayed towards traditional bone setters, and they might lose funds or relevance while adopting a stratified model of care. Studies show that truly, patients could be unappeased with their treatment with stratified care and this can be significantly linked to their expectations (62). This is consistent with a recent study showing that due to dissatisfaction with their treatment, some patients have gravitated towards treatment by traditional bone setters (63). From the patient's point of view, it can be derived that ideas on treatment goals, preference for passive treatment, how much treatment and the nature of treatment necessary for someone in their condition are being influenced by their clinicians and have strongly shaped their belief system and expectations. This has a bearing on the attitude of patients towards stratified care and their expectations (15).

Patients in this study suggested that it is the responsibility of their clinician to provide the conviction needed to secure their co-operation in stratified care implementation. Some believe that if their clinicians properly introduced it to them via education and meaningful communication, it would be seamless. These findings agree with previous research indicating that patients valued health care professionals who not only listened to them (64, 65), also took time to explain their conditions and options in lay terms and helped them navigate the healthcare system (66). This reveals a mismatch in understanding between patients and physiotherapists and an intrinsic strategy for implementation. The patients have expectations, but these are not as strong as the physiotherapists believe and are fuelled by the therapists themselves. Hence, the therapists can modify the patient's expectations strongly by first, being aware of the areas where these differences in perspectives exist. Studies suggest that with further training, experience and learning from open-minded clinical encounters divergent areas can be identified and managed (67, 68). Secondly, change in physiotherapists beliefs, enhanced communication and strides aimed at patient conviction can contribute to modifying patients' expectations (69).

Another key issue raised as a barrier to implementation is physiotherapist competency in the areas of psychosocial care for high-risk patients. This is a key area of significance paramount in other contexts and among other health practitioners (61, 70). Physiotherapists express concerns about perceived deficiencies in training as seen in previous research (71, 72). Training is required for physiotherapists to deliver a broader biopsychosocial model of care (73) and a key component for successful implementation as shown in research (43, 58). Some question their competence in handling high-risk patients majorly highlighting therapists' need for competency in the psychosocial aspect of patient care. This is consistent with studies that show physiotherapists today do not feel adequately trained to deal with complex high-risk patients (59, 51).

For peer review only - http://bmjopen.bmj.com/site/about/guidelines.xhtml

Patients however trust in the physiotherapist in terms of training and competence. This could be due to their experiences because in seeking help, many have resorted to a variety of care from traditional massage and bone setting to the use of devices. Patients relate they have come to trust their therapists and have discovered from experience that being involved in their care, self-management and an active lifestyle is more beneficial. Such understanding drawn from experience could be a motivation for behaviour change in the direction of implementation (58). This leaves a new role for physiotherapists, providing enablement for their patients instead of dependency (74, 59). Concordant to this, research reveals that this supporting role should include reducing barriers and increasing the patients' capability to self-manage (58).

For physiotherapists, major suggestions made were to enforce paradigm shifts in standardising practice, early undergraduate training and workplace mentorship. These interventions support lasting behavioural change (58) and have a bearing on the success or failure of the implementation efforts (75). Key strategies to implementation are the stepwise introduction of the concept to colleagues especially those of speciality groups relevant to musculoskeletal physiotherapy, emphasising the research results and lobbying the seniors to key in. Some physiotherapists relate that hierarchical implementation has great potential to aid practice among physiotherapists, the driving influence of other clinicians in the higher cadre, senior/supervisors can have a positive impact on the quality of health care delivery offered by junior physiotherapists and hence implementation (76, 77).

Suggestions on where implementation should commence were also paramount in the discussions. Physiotherapists relate that implementation efforts should be focused first on the government hospitals. Regarding the sub-groups, physiotherapists practising in federal settings have the opinions that the aspect of high-risk management will not work in federal settings, due to the high patient load as two studies confirm (78, 79). They feel the assessment and delivery of psychosocial treatment will be a problem due to time. They have the opinion it will work better in the private settings, because private sector physiotherapists have a lesser patient load and perhaps more time. This is consistent with the STarT-Back trial where sufficient assessment time of 45 minutes allocated to patients in the high-risk category contributed to a successful implementation outcome (19).

However, physiotherapists in our study felt the low-risk approach will work better because the therapists are paid by the government and they will welcome the idea as it helps to reduce the patient load (78, 79). The private physiotherapists however opined that they need the patients to keep coming, due to financial reasons, hence the low-risk approach will be undesirable. However, they welcome the high-risk approach, as it will improve the quality of care, but the time for assessment will be an issue also as they too have limited time allocated for assessment as research shows (80, 79). Further research reveals that private practice is more satisfactory to patients in terms of interpersonal quality of care, more

For peer review only

attentive personnel, and a broader choice of provider (81, 79). The patients in our study, similar to participants in a recent review by Lim et al. (2019), require conviction, quality communication and time for successful implementation (82).

A strength of this study is its design, adhering to a rigorous method to systematically and meticulously draw out comparisons and variations until saturation was reached. The sampling strategy in this study ensured that physiotherapists and patients in both urban and rural settings were contacted giving equal opportunity for referral and response. The sample thus included participants of varied sex and age, physiotherapists from different contexts, patients with acute and chronic health problems, patients who visited the physiotherapist and other health care institutions, and importantly, our sample comprised patients of all risk groups in the STarT-Back risk classifications. These were checked severally until saturation was reached.

One limitation of this study might be in the presentation of findings. A common challenge with presenting results in Grounded theory is the degree of detail in outcomes (83). However, through constant comparisons, the authors created sufficient connections between the highest levels of abstraction and the data through variations. These were thoroughly explained ensuring all aspects of the data were represented.

This study was carried out in Nigeria, hence care should be taken when generalising results to other countries. However, having context factors in mind we believe that due to the rigour of the study, interesting comparisons based on these results can be made (15) .

Additionally, the researchers' own subjectivities, beliefs and experiences might have played an important role. This is not necessarily a limitation, but can be seen as an inevitable and integral part of using grounded theory compared with other qualitative methods such as content analysis (84, 85). A challenge that was overcome during this study was getting the participants motivated to engage in uninterrupted productive discussions. This was achieved by emphasizing the relevance of the interviews early during the recruitment process, keeping in mind that stakeholders are often interested in knowledge co-creation and mobilisation when the relevance is succinct (86). Thus, comprehensive and rich interviews were achieved with participants in a comfortable atmosphere.

Conclusion

Perspectives of patients about stratified care differ from that of physiotherapists, and both should be aware of divergent areas needing resolution. Emphasis should thus be placed on quality communication among stakeholders. Such communication requires skill on the part of the physiotherapist and sufficient time to deliver, both of which are considered barriers to implementation of the intervention.

For peer review only

Other meaningful barriers identified in this study include the treatment expectations held by patients regarding the method and success of intervention and the incentivised attachment to the usual practice provided by physiotherapists such as using passive methods.

This study further reveals the use of facilitators like patients' trust, speciality groups and contextual adaptations has the potential to aid the implementation of stratified care. To integrate this into clinical practice, a consensus is required from the physiotherapists on key strategies for tailoring these findings, identifying and prioritising approaches to utilise these facilitators, taking care to overcome the barriers.

Conflicts of interest

The authors declare that there is no conflict of interest

Funding

MA received personal funding from the German Academic Exchange Service (DAAD) and Petroleum Technology Development Fund (PTDF) for his doctoral project.

The authors declare that there is no external source of funding allocated to this project.

Abbreviations

STarT: Subgrouping for Targeted Treatment
SBT: STarT Back Tool
MSK: Musculoskeletal
LBP: Low back pain

Contributions

MA: Designed the study, recruited participants, organised data collection, transcribed, analysed and interpreted the data and drafted the manuscript.

JS: Designed the study, analysed and interpreted the data, critically revised the manuscript.

KS: Designed the study, analysed and interpreted the data, critically revised the manuscript.

CM: Designed the study, recruited participants, critically revised the manuscript.

SK: Designed the study, analysed and interpreted the data, drafted the manuscript, critically revised the document.

MA and SK conceived and supervised all aspects of the project, therefore they are responsible for the overall content as the guarantor.

All authors read and approved the final version of the manuscript.

Acknowledgements

The authors express thanks to Faith Ikpami and Ogbulie Smart for their help to recruit patients; Jonathan Hill and Zipporah Echigeme for their input on the introductory materials; Anneke Wolf for her ideas and help setting up the online survey and Cedric Bender for his ideas on analysis.

Data availability statement

All data relevant to the study are included in the article or uploaded as supplemental information.

For peer review only

References

1. Adegoke OA, Akodu AK, Oyeyemi AL. Work-related musculoskeletal disorders among Nigerian physiotherapists. BMC Musculoskeletal Disorders 2008; 9(1):112.

2. Ayanniyi O, Lasisi O, Adegoke B, Oni-Orisan M. Management of low back pain: attitudes and treatment preferences of physiotherapists in Nigeria. African Journal of Biomedical Research 2009; 10(1):42–9.

3. Oyinloye OI, Abdulkadir AY, Babalola OM. Incidence and patterns of lumbosacral transitional vertebrae, in patients with Low Back Pain in a Nigerian hospital. Nigerian Quarterly Journal of Hospital Medicine 2009; 19(2):95–9.

4. Duthey B. Background Paper. Low back pain: Priority medicines for Europe and the world "A Public Health Approach to Innovation"; 2013 [cited 2020 May 9]. Available from: URL: https://www.who.int.

5. Bello B, Muhammad T. Economic cost of low back pain management in Kano Nigeria. Advances in Life Science and Medicine 2017; 3(1):1–8.

6. German Medical Association. National Programme for Disease Management Guidelines Responsible agencies: Method Report; 2010 [cited 2019 Sep 9]. Available from: URL: http://www.baek.de.

7. Odole A, Akinpelu A, Adekanla A, Obisanya B. Economic burden of low back pain on patients seen at the outpatient physiotherapy clinics of secondary and tertiary health institutions in Ibadan. Journal of the Nigeria Society of Physiotherapy 2011; 18(18):43–8.

8. Buchbinder R, van Tulder M, Öberg B, Costa LM, Woolf A, Schoene M, Croft P. Low back pain: a call for action. The Lancet 2018; 391(10137):2384–8.

9. Hartvigsen J, Hancock MJ, Kongsted A, Louw Q, Ferreira ML, Genevay S, Hoy D, Karppinen J, Pransky G, Sieper J, Smeets R, Underwood M. What low back pain is and why we need to pay attention. The Lancet 2018; 391(10137):2356–67.

10. German Medical Association. National Health Care Guideline Non-specific low back pain long version; 2017 [cited 2020 Jan 10]. Available from: URL: www.kreuzschmerz.versorgungsleitlinien.de.

11. Trauma Programme of Care National Low Back and Radicular Pain Pathway 2017. UK; 2017 [cited 2019 Oct 30]. Available from: URL: https://www.versusarthritis.org.

12. KNGF Guideline. Royal Dutch Society of Physiotherapy [cited 2019 Dec 10]. Available from: URL: http://www.ipts.org.il.

13. Foster NE, Anema JR, Cherkin D, Chou R, Cohen SP, Gross DP, Ferreira PH, Fritz JM, Koes BW, Peul WC, Turner JA, Maher CG, Buchbinder R, Hartvigsen J, Underwood M, van Tulder M. Prevention and treatment of low back pain: evidence, challenges, and promising directions. The Lancet 2018; 391(10137):2368–83.

14. Cruz EB, Canhão H, Fernandes R, Caeiro C, Branco JC, Rodrigues AM, Pimentel-Santos F, Gomes L, Paiva S, Pinto I, Moniz R, Nunes C. Prognostic indicators for poor outcomes in low back pain patients consulted in primary care. PLoS ONE 2020; 15(3):e0229265.

15. Sowden G, Hill JC, Morso L, Louw Q, Foster NE. Advancing practice for back pain through stratified care (STarT Back). Brazilian Journal of Physical Therapy 2018; 22(4):255–64.

16. Whitehurst DG, Bryan S, Lewis M, Hill J, Hay EM. Exploring the cost-utility of stratified primary care management for low back pain compared with current best practice within risk-defined subgroups. Annals of the Rheumatic Diseases 2012; 71(11):1796–802.

17. Fritz JM, Beneciuk JM, George SZ. Relationship between categorization with the STarT Back Screening Tool and prognosis for people receiving physical therapy for Low Back Pain. Physical Therapy 2011; 91(5):722–32.

18. Foster NE, Mullis R, Hill JC, Lewis M, Whitehurst DG, Doyle C, Konstantinou K, Main C, Somerville S, Sowden G, Wathall S, Young J, Hay E. Effect of stratified care for low back pain in family practice (IMPaCT back): A prospective population-based sequential comparison. Annals of Family Medicine 2014; 12(2):102–11.

19. Hill JC, Whitehurst DG, Lewis M, Bryan S, Dunn KM, Foster NE, Konstantinou K, Main C, Mason E, Somerville S, Sowden G, Vohora K, Hay E. Comparison of stratified primary care management for low back

For peer review only

pain with current best practice (STarT Back): A randomised controlled trial. The Lancet 2011; 378(9802):1560–71.

20. Hill JC, Dunn KM, Lewis M, Mullis R, Main CJ, Foster NE, Hay E. A primary care back pain screening tool: Identifying patient subgroups for initial treatment. Arthritis Care and Research 2008; 59(5):632–41.

21. Sowden G, Hill JC, Konstantinou K, Khanna M, Main CJ, Salmon P, Somerville S, Wathall S, Foster N. Targeted treatment in primary care for low back pain: The treatment system and clinical training programmes used in the impact back study. Family Practice 2012; 29(1):50–62.

22. Foster NE, Hill JC, O'Sullivan P, Hancock M. Stratified models of care. Best Practice & Research Clinical Rheumatology 2013; 27(5):649–61.

23. Baxter GD, Chapple C, Ellis R, Hill J, Liu L, Mani R, Reid D, Stokes T, Tumilty S. Six things you need to know about low back pain. Journal of Primary Health Care 2020; 12(3):195–8.

24. Cherkin D, Balderson B, Wellman R, Hsu C, Sherman KJ, Evers SC, Sarah C, Hawkes R, Cook A, Levine M, Piekara D, Rock P, Estlin K, Brewer G, Jensen M, LaPorte A, Yeoman J, Sowden G, Hill J. Effect of low back pain risk-stratification strategy on patient outcomes and care processes: the MATCH randomized trial in primary care. Journal of General Internal Medicine 2018; 33(8):1324–36.

25. Ogbeivor C, Elsabbagh L. Management approach combining prognostic screening and targeted treatment for patients with low back pain compared with standard physiotherapy: A systematic review & meta-analysis Musculoskeletal Care 2021. Epub ahead of print.

26. Bernstein IA, Malik Q, Carville S, Ward S. Low back pain and sciatica: summary of NICE guidance. BMJ Online 2017; 356: 6748.

27. Beneciuk JM, Hill JC, Campbell P, Afolabi E, George SZ, Dunn KM et al. Identifying Treatment Effect Modifiers in the STarT Back Trial: A Secondary Analysis. Journal of Pain 2017; 18(1):54–65.

28. Akodu AK, Kareem RO, Faniyi OR. Management of low back pain: knowledge and adherence to clinical practice guidelines amongst physiotherapists in selected hospitals in Lagos state. Research Journal of Health Sciences 2016; 4(3):203.

29. Igwesi-Chidobe CN, Kitchen S, Sorinola IO, Godfrey EL. "A life of living death": the experiences of people living with chronic low back pain in rural Nigeria. Disability and Rehabilitation 2017; 39(8):779–90.

30. Igwesi-Chidobe CN, Coker B, Onwasigwe CN, Sorinola IO, Godfrey EL. Biopsychosocial factors associated with chronic low back pain disability in rural Nigeria: a population-based cross-sectional study. BMJ Global Health 2017; 2(3):e000284.

31. Willig C, Stainton-Rogers W, editors. The SAGE Handbook of Qualitative Research in Psychology. London England: SAGE Publications; 2008.p 123-140.

32. Corbin JM, Strauss A. Grounded theory research: Procedures, canons, and evaluative criteria. Qualitative Sociology 1990; 13(1):3–21.

33. Tong A, Sainsbury P, Craig J. Consolidated criteria for reporting qualitative research (COREQ): A 32-item checklist for interviews and focus groups. International Journal for Quality in Health Care 2007; 19(6):349–57.

34. Elder NC, Miller WL. Reading and evaluating qualitative research studies. Journal of Family Practice 1995; 41(3):279–85.

35. Palinkas LA, Horwitz SM, Green CA, Wisdom JP, Duan N, Hoagwood K. Purposeful sampling for qualitative data collection and analysis in mixed method implementation research. Administration and Policy in Mental Health 2015; 42(5):533–44.

36. Meerhoff GA, van Dulmen SA, Maas MJM, Bakker-Jacobs A, Nijhuis-Van der Sanden MWG, van der Wees PJ. Exploring the perspective of patients with musculoskeletal health problems in primary care on the use of patient-reported outcome measures to stimulate quality improvement in physiotherapist practice; a qualitative study. Physiotherapy Theory and Practice 2019: 37(9):993-1004.

37. Mason M. Sample size and saturation in PhD studies using qualitative interviews. Qualitative Social Research 2010; 11(3):1–13.

For peer review only

38. Norman MK. Twelve tips for reducing production time and increasing long-term usability of instructional video. Medical Teaching 2017; 39(8):808–12.

39. Main CJ, Sowden G, Hill JC, Watson PJ, Hay EM. Integrating physical and psychological approaches to treatment in low back pain: The development and content of the STarT Back trial's 'high-risk' intervention. Physiotherapy 2012; 98(2):110–6.

40. Staniszewska S, Brett J, Simera I, Seers K, Mockford C, Goodlad S, D Altman, D Moher, R Barber, Dengeri S, Entwistle A, Littlejohns P, Morris C, Suleman R, Thomas V, Tystall C. GRIPP2 reporting checklists: tools to improve reporting of patient and public involvement in research. BMJ (Online) 2017; 358, j3453

41. Heistinger A. Qualitative Interviews – A guide to preparation and implementation including some theoretical notes [cited 2020 Oct 25]. Available from: URL: https://www.uibk.ac.at.

42. Burke LA, Miller MK. Phone interviewing as a means of data collection: lessons learned and practical recommendations. Qualitative Social Research 2001; 2(2):7.

43. Damschroder LJ, Aron DC, Keith RE, Kirsh SR, Alexander JA, Lowery JC. Fostering implementation of health services research findings into practice: a consolidated framework for advancing implementation science. Implementation Science 2009; 4(1):50.

44. Moullin JC, Sabater-Hernández D, Fernandez-Llimos F, Benrimoj SI. A systematic review of implementation frameworks of innovations in healthcare and resulting generic implementation framework. Health Research Policy and Systems 2015; 13(1):1–11.

45. Döringer S. 'The problem-centred expert interview'. Combining qualitative interviewing approaches for investigating implicit expert knowledge. International Journal of Social Research Methodology 2020:1–14.

46. Pepper D, Hodgen J, Lamesoo K, Kõiv P, Tolboom J. Think aloud: using cognitive interviewing to validate the PISA assessment of student self-efficacy in mathematics. International Journal of Research and Method in Education 2018; 41(1):3–16.

47. Dresing, T, Pehl T, editor. Practice book interview and transcription; 2012 [cited 2020 Jan 9]. Available from: URL: https://www.audiotranskription.de/Praxisbuch.

48. Tessier S. From field notes, to transcripts, to tape recordings: evolution or combination? International Journal of Qualitative Methods 2012; 11(4):446–60.

49. Sbaraini A, Carter SM, Evans RW, Blinkhorn A. How to do a grounded theory study: a worked example of a study of dental practices. BMC Medical Research Methodology 2011; 11(1):128.

50. R Development Core Team. R: A tool for statistical computing. Vienna, Austria: R foundation for statistical Computing 2014. [cited 2019 Nov 8]. Available from: URL: http://www.R-project.org.

51. Sanders T, Ong BN, Sowden G, Foster N. Implementing change in physiotherapy: professions, contexts and interventions. Journal of Health Organization and Management 2014; 28(1):96–114.

52. Odole AC, Afolabi KO, Ushie BA, Odunaiya NA. Views of physiotherapists from a low resource setting about physiotherapy at a distance: a qualitative study. European Journal of Physiotherapy 2020; 22(1):14–9.

53. Abaraogu UO, Aguji KR, Duru DO, Okafor UC, Ezeukwu AO, Igwe SE. Physiotherapist-patient communication in entry-level physiotherapy education: A national survey in Nigeria. Hong Kong Physiotherapy Journal 2019; 39(1):77–87.

54. Cowell I, O'Sullivan P, O'Sullivan K, Poyton R, McGregor A, Murtagh G. The perspectives of physiotherapists on managing nonspecific low back pain following a training programme in cognitive functional therapy: A qualitative study. Musculoskeletal Care 2019; 17(1):79–90.

55. Synnott A, O'Keeffe M, Bunzli S, Dankaerts W, O'Sullivan P, Robinson K, O'Sullivan K. Physiotherapists report improved understanding of and attitude toward the cognitive, psychological and social dimensions of chronic low back pain after Cognitive Functional Therapy training: a qualitative study. Journal of Physiotherapy 2016; 62(4):215–21.

56. Odole AC, Oyewole OO, Akinpelu AO. A comparative survey of Nigerian physiotherapists' familiarity with, knowledge of and utilisation of standard outcome measures: 10 years after initial survey. South African Journal of Physiotherapy 2018; 74(1):435.

For peer review only

57. Akindele M, Rabiu M, Useh E. Assessment of the awareness, adherence, and barriers to low back pain clinical practice guidelines by practicing physiotherapists in a low-resourced country. Physiotherapy research international: The Journal for Researchers and Clinicians in Physical Therapy 2019; 25(1):e1811.

58. Michie S, van Stralen MM, West R. The behaviour change wheel: a new method for characterising and designing behaviour change interventions. Implementation Science 2011; 6(1):42.

59. Holopainen R, Piirainen A, Karppinen J, Linton SJ, O'Sullivan P. An adventurous learning journey. Physiotherapists' conceptions of learning and integrating cognitive functional therapy into clinical practice. Physiotherapy Theory and Practice 2020: Epub ahead of print.

60. Holopainen R, Piirainen A, Heinonen A, Karppinen J, O'Sullivan P. From "Non-encounters" to autonomic agency. Conceptions of patients with low back pain about their encounters in the health care system. Musculoskeletal Care 2018; 16(2):269–77.

61. Karstens S, Kuithan P, Joos S, Hill JC, Wensing M, Steinhäuser J, Katja K, Joachim S. Physiotherapists' views of implementing a stratified treatment approach for patients with low back pain in Germany: a qualitative study. BMC Health Services Research 2018; 18(1):1–12.

62. Riis A, Jensen CE, Bro F, Maindal HT, Petersen KD, Bendtsen MD, Jensen MB. A multifaceted implementation strategy versus passive implementation of low back pain guidelines in general practice: a cluster randomised controlled trial. Implementation Science 2016; 11(1):143.

63. Mbada CE, Ojoawo AO, Owoola SO, Okonji AM, Odetunde MO, Adigwe KC Makinde MO, Adegbemigun OD, Fasuyi FO, Idowu OA, Fatoye F. Knowledge and attitude about the practice of traditional bone setters and its use for musculoskeletal disorders in rural areas in Nigeria. Middle East Journal of Rehabilitation and Health Studies 2020; 7(2):e99973.

64. Lewis J, O'Sullivan P. Is it time to reframe how we care for people with non-traumatic musculoskeletal pain? British Journal of Sports Medicine 2018; 52(24):1543–4.

65. Karstens S, Lang S, Saunders B. Patients' views on the implementation potential of a stratified treatment approach for low back pain in Germany: a qualitative study. Health Services Insights 2020; 13:1-8.

66. O'Malley AS, Peikes D, Wilson C, Gaddes R, Peebles V, Day TJ, Janel J. Patients' perspectives of care management: a qualitative study. The American Journal of Managed Care 2017; 23(11):684–9.

67. Bastemeijer CM, van Ewijk JP, Hazelzet JA, Voogt LP. Patient values in physiotherapy practice, a qualitative study. Physiotherapy Research International: The Journal For Researchers And Clinicians In Physical Therapy 2021; 26(1):e1877.

68. Cooper I, Delany C, Jenkins S. Comparing patients' and physiotherapists' views of professionalism and professional standing: an Australian perspective. Physical Therapy Reviews 2016; 21(1):38–73.

69. Le Laekeman M, Sitter H, Basler H. The Pain Attitudes and Beliefs Scale for Physiotherapists: psychometric properties of the German version. Clinical Rehabilitation 2008; 22(6):564–75.

70. Karstens S, Joos S, Hill JC, Krug K, Szecsenyi J, Steinhäuser J. General practitioners' views of implementing a stratified treatment approach for low back pain in Germany: a qualitative study. PLOS ONE 2015; 10(8):e0136119.

71. Abaraogu UO, Onah U, Abaraogu OD, Fawole HO, Kalu ME, Seenan CA. Knowledge, attitudes, and the practice of health promotion among physiotherapists in Nigeria. Physiotherapy Canada 2019; 71(1):92–100.

72. Onyeso OK, Umunnah JO, Ezema CI, Balogun JA, Uchenwoke CI, Nwankwo MJ, Oke K, Bello B, Nwosu I, Adje M. An evaluation of the nature and level of musculoskeletal imaging training in physiotherapy educational programmes in Nigeria. BMC Medical Education 2020; 20(1):252.

73. Cowell I, O'Sullivan P, O'Sullivan K, Poyton R, McGregor A, Murtagh G. Perceptions of physiotherapists towards the management of non-specific chronic low back pain from a biopsychosocial perspective: A qualitative study. Musculoskeletal Science and Practice 2018; 38:113–9.

74. Nijs J, Roussel N, van Paul Wilgen C, Köke A, Smeets R. Thinking beyond muscles and joints: therapists' and patients' attitudes and beliefs regarding chronic musculoskeletal pain are key to applying effective treatment. Manual Therapy 2013; 18(2):96–102.

For peer review only

75. Ulrich G, Homberg A, Karstens S, Mahler C. Attitudes towards interprofessional collaboration in young healthcare professionals. Journal of Interprofessional Care 2019; 33(6):768–73.

76. Ezenwankwo EF, Ezeukwu AO, Abaraogu UO, Manu GP, Mogbolu GA, Ezelioha RN, Ifediora, LP, Uchenwoke CI. Physiotherapy clinical education in the eastern Nigeria: students' and interns' views on clinical educator characteristics, opportunities and learning conditions. European Journal of Physiotherapy 2019; 21(3):153–63.

77. Onigbinde A. The impact of internship programme and its effects on the physiotherapy practice in Nigeria 2007 [cited 2019 Sep 9]. Available from: URL: https://www.semanticscholar.org

78. DO Odebiyi, CB Aiyejusunle, TS Ojo, BA Tella. Comparison of patients satisfaction with physiotherapy care in private and public hospitals. Journal of Nigerian Society of Physiotherapy 2009; 17(1):23–9.

79. Odumodu IJ, Olufunlayo TF, Ogunnowo BE, Kalu ME. Satisfaction with services among attendees of physiotherapy outpatient clinics in tertiary hospitals in Lagos state. Journal of Patient Experience 2020; 7(4):468–78.

80. Onyeso OKK, Umunnah JO, Ezema CI, Anyachukwu CC, Nwankwo MJ, Odole AC, Oke KI, Bello B. Profile of practitioners, and factors influencing home care physiotherapy model of practice in Nigeria. Home Health Care Services Quarterly 2020; 39(3):168–83.

81. Hanson K, Goodman C, Lines JD, Meek S, Bradley D, Mills A. The economics of malaria control interventions. World Health Organization; 2004 [cited 2019 Sep 9]. Available from: URL: http://apps.who.int/iris.

82. Lim YZ, Chou L, Au RT, Seneviwickrama KMD, Cicuttini FM, Briggs AM, Sullivan K, Urquhart DM, Wluka AE. People with low back pain want clear, consistent and personalised information on prognosis, treatment options and self-management strategies: a systematic review. Journal of Physiotherapy 2019; 65(3):124–35.

83. Backman, K.; Kyngäs, H. A. Challenges of the grounded theory approach to a novice researcher. In Nursing & health sciences 1999; 1 (3), pp. 147–153.

84. Strübing J. Grounded Theory: Zur sozialtheoretischen und epistemologischen Fundierung des Verfahrens der empirisch begründeten Theoriebildung. Wiesbaden: VS Verlag für Sozialwissenschaften; 2004. (Qualitative Sozialforschung; vol 15).

85. Holstein J, Gubrium J. Active interviewing. In: Silverman D, editor. Qualitative research: theory, method and practice. London: Sage; 1997. p. 113–29

86. Holmström I, Röing M. The relation between patient-centeredness and patient empowerment: a discussion on concepts. Patient Education and Counseling 2010; 79(2):167–72.

For peer review only

Supplemental Table 1: Interview guideline for patients

Key Question	Maintenance question	Potential follow-up questions
1. Experiences (Warm-up): Tell me something about the LBP treatment you received so far.	- What else?<br>- And so on?<br>- Is there anything else	a) What is your expectation of a successful treatment for your LBP?<br>b) What are your concerns regarding the treatment of LBP?
2. Exploration: What is your opinion on the SB approach?	- What else?<br>- And so on?<br>- Is there anything else?	c) How would you feel being classified into a subgroup based on the SB tool ?<br>d) What would be your reaction if you were offered to be treated with this approach?<br>e) What can you say about the questions in the SB tool?
3. Factors: How does this approach compare with the current treatment you receive?	- What else?<br>- And so on?<br>- Is there anything else?	f) Mention any specific aspect of the SB approach which interests you?<br>g) What are your opinions regarding the tool process?<br>h) What do you think about communication with the therapist in this approach?<br>i) In the video, patients with a low likelihood of poor outcome receive one treatment session with examination and advice, how do you feel about this?<br>j) In the video, patients with a high likelihood of poor outcomes receive physical and psychological treatment, how do you feel about this?<br>k) In your opinion, what do you expect from physiotherapists who use this approach e.g. attitude, training, expertise?<br>l) What is your opinion on a patient's responsibility regarding this approach?<br>m) In your opinion, tell me what is required to use this approach in Nigeria?<br>n) Tell me if other enablers or barriers come to your mind when you think of this approach?
4. Related influences: What do you think might happen when this approach is used?	- What else?<br>- And so on?<br>- Is there anything else	o) How do you think this approach affects you as a patient with LBP?<br>p) How does this approach affect how you view treatment for LBP in Nigeria?

LBP: Low back pain; SB: STarT-Back

Supplemental Table 2: Interview guideline for physiotherapists

Key Question	Maintenance question	Potential follow-up questions
1. Exploration: What is your perception of the SB approach?	• What else?<br>• And so on?<br>• Is there anything else?	a) Tell me something about your current approach to treatment for non-specific LBP.<br>b) How do you consider the idea of classifying patients into subgroups based on the SB tool?<br>c) How would you feel about allocating treatments in this treatment approach?
2. Factors: How does this approach compare with the current treatment for non-specific LBP in Nigeria?	• What else?<br>• And so on?<br>• Is there anything else?	d) Mention any specific attributes of the SB approach that might interest you.<br>e) What can you say about the SB tool?<br>f) What is your opinion about the patient-centeredness of this approach?
3. Strategies: Tell me any possible requirements for implementing this approach in Nigeria?	• What else?<br>• And so on?<br>• Is there anything else?	g) What are your ideas on the handling of the tool?<br>h) In your opinion, what is required for the implementation of the High-risk approach?<br>i) In your opinion, what is required for the implementation of the Low-risk approach?<br>j) What are your ideas about incentives for using this approach?<br>k) Tell me your ideas about training on this approach.<br>l) What comes to your mind when you think about the distribution of this approach in Nigeria?<br>m) Tell me if other enablers and barriers come to your mind when you think of this approach?
4. Related influences: What effects do you think might result from its use?	• What else?<br>• And so on?<br>• Is there anything else?	n) How might this approach affect patients receiving physiotherapy in Nigeria?<br>o) How might it affect interprofessional relations in Nigeria?

LBP: Low back pain; SB: STarT-Back

Supplemental Table 3: Coding agenda with category descriptions

**Category 1: Resistance to change.**<br><br>**Themes of variation**	**Category Description: A mixed category because it describes views from patients and physiotherapists. It describes the resistance perceived and experienced by physiotherapists and patients concerning introducing a stratified care approach. It describes the physiotherapists and patients' perceptions of challenges to the paradigm shift and strategies to overcome resistance.**		
	**Resistance to change**	**Explanation**	**Quotes***
**Tradition of treatment**<br>This describes components of the usual care, highlighting gaps in practice and aspects that can be modified to optimise care.	Incentives attached to the usual practice	Physiotherapists tradition of treatment was a major challenge, it seemed to be an overwhelming issue sustained by Incentives attached to the usual practice. Key aspects are ego, self-confidence in their current practice experience and finance. There is a long-held tradition of treatment with non-evidence-based methods, diagnosis and radiographs that the patient has gotten used to and this tradition remains deeply entrenched in practice.	***PTf7:*** *I would say it has a lot to do with ego, they won't want you to tell them what to do... If I go for training and come back with a new trend, I would start using it immediately, but your colleagues, now avoiding it because it comes from your colleague.*<br><br>***PTf7:*** *Physiotherapists usually shy away when there is a new protocol, implementing it will just be for record purposes, they think it will stress them and is time-wasting.*
**Evolution to a new system**<br>This discusses the key enablers needed to introduce a new practice aimed at optimising care.	Overcoming patients' expectations	Therapists see patient expectation *as* a primary deterrent to the implementation of stratified care that needs to be addressed. It feels to them like all their suggestions and efforts to optimising practice will not work because the patients come with a certain expectation. They expect a strong touch, massage or something physical that would bring the pain down to zero. There is, however, an enabling situation seen by therapists and patients, that these expectations can be adjusted and overcome.	***PatF1:*** *I wish it can go, I wish my pain can go… I think it will need some kind of strong force for it to go, maybe I should like a fall or something to put it in position.*<br><br>***PTm5:*** *I think it's just marrying the expectations of the patient with what the physiotherapist thinks is realistic, and with that, there could be some kind of trade-off.*
**Experiences**<br>This describes instances where attempts to install change have been made and the effects	Organisational culture	From the experiences of physiotherapists and patients, the organisational culture preponderant in Nigeria makes treatment less efficient and less effective. They have limitations in their ability to ease the changes beyond a certain point like organising situations, follow-ups or influence patient's working conditions.	***PatM2:*** *If you tell me to modify my workstation and I know that I cannot do it you are wasting your time. but if you tell me the office seat needs to be changed and in one week someone brings the right one I will have the right attitude.*<br><br>***PTm9****: There might be challenges, the kind of environment and mentality here in Nigeria is different.*
**Strategies for implementation**<br>This highlights the participants' key ideas on strategies for the implementation of the stratified care approach.	Targeting attitudinal change	A change in the attitude of physiotherapists and patients is seen as a major strategy to improve the chances of successful implementation. The physiotherapist and patients see themselves as mostly complacent. The patients blamed the physiotherapists for not being attentive to their needs and the physiotherapists blamed the patient's attitude as a deterrent for being unable to improve their condition.	***PatM8:*** *The period I was there I don't think there was a lot of Patient load, we were not more than ten patients attending physiotherapy so I think it is their attitude that needs to change.*<br><br>***PTf8:*** *Well, I have tried to push it, and it didn't work…some patients have an attitude like you are trying to test their knowledge…so somehow this attitude has affected the use by physiotherapists.*

* Physiotherapists' and patient's quotes with pseudonymous designations comprising the numerical order of interviews.

For peer review only

**Category 2: Acceptance of innovation**<br><br>**Themes of variation**	**Category description: This is a category most relevant to physiotherapists. It describes a positive reaction from physiotherapists to the idea of implementation of stratified care in Nigeria, highlighting their perspectives on the best settings and enabling conditions for implementation with inputs from patients.**		
	**Acceptance of innovation**	**Explanation**	**Quotes***
**Tradition of treatment**<br>This describes components of the usual care, highlighting gaps in practice and aspects that can be modified to optimise care.	Ease of transition	Some physiotherapists reported similarity of stratified care to what they already know (tradition of treatment) and practice in their management of LBP, they have background knowledge of the biopsychosocial approach and vary treatment for patients. These are individual aspects of stratified care and participants reported that this makes it easy to fully adopt the stratified care in its entirety.	***PTf7:*** *You are just giving it a name, it is comparable to what we do already here…. well, now all patients are treated more individually, we do not use the same intervention for every LBP patient anymore we have improved.*<br><br>***PTf11:*** *Yes, just as I said it's not really different from what we do here, …we do not have a name for it but it is essentially the same as what this approach is about, but I can speak for my centre only. because I will say my centre is about the best in Nigeria in terms of the content of practice so far.*
**Evolution to a new system**<br>This discusses the key enablers needed to introduce a new practice aimed at optimising care.	Open to new knowledge	Physiotherapists realising this approach is fundamentally new, uncommon and not used in its entity in practice, yet they were open to new knowledge describing stratified care as an improvement to current care and welcome development.	***PTm1****: So far, I think it is a welcome development because the approach is nice because it tends to categorise individuals with LBP in two categories people that have low risk moderate and high categorising individuals into group will give it a definition into the line of management or better outcome.*<br><br>***PTf4:*** *My perception in summary about the approach is that it is a good one and that it would help physiotherapist a lot.*
**Experiences**<br>This describes instances where attempts to install change have been made and the effects	Steps towards optimising practice	From their experiences, participants came across situations where they made steps towards optimising practice, instances where innovations, ideas or approaches have been introduced in their various work settings, the process that helped and the positive responses from colleagues. Key aspects were the use of clinical discussions, through physiotherapists cadre and time-saving measures for the patient. They think the stratified care being introduced in a similar stepwise manner will be successful.	***PTf7:*** *Since everyone is on board, especially our head of department, he likes such things, if he sees the evidence he would say 'from today we are using this approach and it must be used and expedite the use.*<br><br>***PTm9:*** *(In cases of innovation in my clinical setting) the same strategy, we discuss it at seminars and everyone brings their opinions and decides to use it and see the result. only then can it become a norm.*
**Strategies for implementation**<br>This highlights the participants' key ideas on strategies for the implementation of the stratified care approach.	Work Settings	There were differing perspectives on the most viable work setting for targeting the implementation of stratified care since there exist different situations and ideologies at the private and government establishments in Nigeria. Participants felt it would be more applicable if targeted at the government hospitals since the therapists are paid by the government a fixed amount and more treatments do not lead to more money.	***PTf12:*** *To be honest the government hospitals make it easier to use these approaches and questionnaires. Because most of the private settings didn't have these things available, so most times I have to source for them to use, but in the government hospitals they were printed and ready for use.*<br><br>***PTm3:*** *Especially in the private practice where you need more patients to have more income, so the more the patients the more the income, so that is the case, it means the physiotherapist might not want to use that approach but, in the government-owned hospitals where that might not be the case, they may be more willing to go in that direction.*

LBP: Low back pain
* Physiotherapists' quotes with pseudonymous designations comprising the numerical order of interviews.

For peer review only

Category 3: Adapting practice<br><br>Themes of variation	Category Description: This category is relevant to patients and physiotherapists. Here participants highlight their opinions and experiences on ways to adopt the stratified care approach. This category describes the suggestions on adaptations in terms of creating awareness and modification of the approach to suit the Nigerian context and enhance its use.		
	**Adapting practice**	**Explanation**	**Quotes***
**Tradition of treatment**<br>This describes components of the usual care, highlighting gaps in practice and aspects that can be modified to optimise care.	Need of standard for regulating the practice	Participants highlighted the absence of a standard of practice tailored to the needs of Nigerians. This situation led to a clamouring for standard to guide practice and regulation. Adherence to such a standard of practice would enhance adaptation for implementation.	***PTf7:*** *In Nigeria, we do not have a standard way of doing things. what hospital A is doing is different from what Hospital B is doing… everyone is just doing what they want.*<br><br>***PatM8:*** *With proper supervision, if the hospital management focuses on what happens in the hospital I am sure there will be room for improvement, for both the staff and the facility.*
**Evolution to a new system**<br>This discusses the key enablers needed to introduce a new practice aimed at optimising care.	Cultural adaptations	Socio-cultural adaptations relating to language, the culture of respect and hierarchical implementation from the seniors to juniors was highlighted as a viable means of adapting practice at the introductory phases.	***PatF5****: The clinician speaks our language…reads to her…or when she comes home the children do.*<br><br>***PTm2:*** *When the chiefs and assistant directors in each unit champion this, then junior physiotherapists will use it.*
**Experiences**<br>This describes instances where attempts to install change have been made and the effects	Use of communication	Communication was seen as a major way to inform adaptation and improve the patient-therapist relationship. When communication between patient and physiotherapist was used it was seen to produce positive effects.	***PTf8****: It is feasible, I always use conversations to help my Patients see the benefits.*<br><br>***PatM2:*** *Well it is communication; a patient needs to know why they are allocated to a particular group.*
**Strategies for implementation**<br>This highlights the participants' key ideas on strategies for the implementation of the stratified care approach.	Awareness for patients and physiotherapists	Participants saw creating awareness about the approach as a major strategy for implementation. While some opined this can be done at undergraduate and post-graduate levels for physiotherapists, patient's awareness was achievable through outreaches.	***PatF9****: And awareness…when I went for their free medical seminar I was able to meet the physiotherapist guy that came for outreach, … which encouraged me.*<br><br>***PTF9:*** *Also, there should be health promotion done to create awareness about it.*

* Physiotherapists' and patient's quotes with pseudonymous designations comprising the numerical order of interviews.

For peer review only

Category 4: Patient's Learning Journey Themes of variation	Category Description: This category is relevant to patients and physiotherapists. Here, patients have experienced care from various fronts and have formed their opinions about what they have experienced to be helpful to their condition. This was corroborated by the physiotherapist opinions about the patients learning experiences from their perspective.		
	**Patient's Learning Journey**	**Explanation**	**Quotes***
**Tradition of treatment** This describes components of the usual care, highlighting gaps in practice and aspects that can be modified to optimise care.	Needing a complement to usual care	For patients, the usual care was seen to be insufficient and there are aspects that patients feel would need some form of compliment, they feel this can be achieved by treatment from traditional bone setters care which is seen as a compliment or an alternative.	***PTm5***: *Most people prefer to go to bonesetters and the likes because the people around them would have told them that this is what someone used and the person got better so.*<br><br>***PatM2:*** *There is a lot in it (traditional bone setters care) that complements orthodox medicine'…I can manage my pain better.*
**Evolution to a new system** This discusses the key enablers needed to introduce a new practice aimed at optimising care.	Recognising unhelpful treatments	Participants report that over the past recent years, patients have gradually become mentally equipped to recognise unhelpful treatments by themselves and developing the ability to differentiate between what is beneficial and what isn't. This realisation is seen as a viable enabler to the implementation of stratified care.	***PatM2:*** *The former treatment was more like going thru a particular regimen and go back to the same condition that brought the pain but the approach …suggests lifestyle changes which if sustained gives permanent relief.*<br><br>***PatM3:*** *Well, to me I tried it and it made matters worse, so I wouldn't advise anyone to do that, to me that is a no-go area because they are working blindly.*
**Experiences** This describes instances where attempts to install change have been made and the effects.	Learning to live with pain	Participants explain how the debilitating condition of the patient's experiences have resulted in personal difficulties. They resolved in their minds to change their outlook and accept the condition and this has improved their outlook.	***PatF9***: *I feel depressed most times, it affects me so much, … I think it is what I would work on now, psychologically I know it is affecting me because I am thinking so much about it and it is giving me so much concern.*<br><br>***PatM3:*** *It affected me psychologically, I was a worrying bit I have learned to live with it, I think I am better at managing myself …so also, I have learned, I have concluded that no kind of intrusive treatment can work for this my case, that I just need to be careful with how I live my life.*
**Strategies for implementation** This highlights the participants' key ideas on strategies for the implementation of the stratified care approach.	Taking charge	Patients have gathered knowledge of self from various treatment exposures, from self-care, traditional bone setters' treatments. They have now decided that taking charge of their treatment is a vital strategy to help themselves.	***PatF7:*** *Yes, what I can generally say is it helped me to know how to better manage the back by myself.*<br><br>***PatF9:*** *I would love to do it (treatment) myself…rather than going to the hospital.*

* Physiotherapists' and patient's quotes with pseudonymous designations comprising the numerical order of interviews.

For peer review only

Category 5: Trusting the physiotherapist<br><br>Themes of variation	Category Description: This category is most relevant to patients. It describes the patient's views of the physiotherapist as a dynamic expert with who they need to give their trust and co-operation based on the efforts of the physiotherapist to help their situation and their experiences with treatments.		
	**Trusting the physiotherapist**	**Explanation**	**Quotes***
**Tradition of treatment**<br>This describes components of the usual care, highlighting gaps in practice and aspects that can be modified to optimise care.	Getting some help	The aspects of the usual care focused on exercise and advice was seen to provide some relief to patients, even though not permanent they relate some improvement in their condition and trust that based on this result more can be expected.	***PatF7****: The therapy I underwent helped though because before I started the treatment I could not even lift myself from the bed …but the first week of the therapy I would say I experienced relief.*<br><br>***PatM3****: I started getting relief because my clinicians were trained, even the advice I received worked to some extent. So, I believe that clinicians treating patients need the training to further update their treatment methods.*
**Evolution to a new system**<br>This discusses the key enablers needed to introduce a new practice aimed at optimising care.	Learning with practice	The patients feel that the physiotherapists will gradually evolve in their practice and that some therapists are already on the right track, learning to change their approach with practice. They feel exploring ideas like this through research is needed to evolve more.	***PatM3:*** *What you are doing is good, research is the main factor so just keep it up. there is no way around it, to give patients the best available treatment there is only one way to make sure of it and that is research. continue to make research that's the future of the medical practice.*<br><br>***PatM4****: It is possible they know these things already, because you know they are experts and they have been dealing with people having the same issues with me for a long time, so they can juxtapose and see if that is the problem or a factor.*
**Experiences**<br>This describes instances where attempts to install change have been made and the effects	Therapists doing their best	The patients acknowledge that the physiotherapists are putting in efforts to optimise care in ways they can and they appreciate the efforts, they notice when some more is done to help their condition and that encourages them to focus more on the efforts.	***PatF9:*** *That therapist tried, he did a lot, I told him I appreciated his work very well…. they tried a lot, I am satisfied with their level of experience.*<br><br>***PatM8****: He talked about some exercise but he advised me majorly about my sleeping position, which has helped so much.*
**Strategies for implementation**<br>This highlights the participants' key ideas on strategies for the implementation of the stratified care approach.	Co-operating with the therapists	The patients consider the physiotherapist's role as providing care and support for the patient when needed and their role as patients is not to interfere with the process. They feel they need to co-operate with the physiotherapist but largely leaving the aspect of care for the therapists.	***PatM5****: For me, I believe that their field and they are the specialists. I can't say, I try as much as possible not to go into areas where I have low competence.*<br><br>***PatM3****: You know when the pain becomes unbearable, you need to consult a specialist, at least to know what is happening and have their support.*

* Patient's quotes with pseudonymous designations comprising the numerical order of interviews.

For peer review only

Category 6: Needing conviction<br><br>Themes of variation	**Category Description: This category is most relevant to patients. It describes patients exposure to false information sources, hence the need for physiotherapists to provide trustworthy education to get their full co-operation. This equips patients on their road to self-discovery and guides them to conviction.**		
	**Needing conviction**	**Explanation**	**Quotes***
**Tradition of treatment**<br>This describes components of the usual care, highlighting gaps in practice and aspects that can be modified to optimise care.	Reliance on investigations	Physiotherapists are already used to a diagnosis-based approach, using radiographs and passive treatments. Since the patients have become used to that, they need some form of conviction to accept the change. Some patients enjoy passive treatments and would not only prefer that but insist it is the right thing to do.	***PTf10****: There is the likelihood that patients will not accept this, they will go after the treatment to another therapist…some tell you how much they enjoy the sessions…they keep coming back to enjoy the sessions.*<br><br>***PatM8****: Investigations are not done. I had to muscle my way to do an MRI by myself, nobody told me about that I just knew it was necessary and I decided to do that.*
**Evolution to a new system**<br>This discusses the key enablers needed to introduce a new practice aimed at optimising care.	Patient education	For patients to be able to accept change, they need to be informed properly to a level of conviction. Patients have multiple sources of information and this needs to be presented clearly and reasonably to influence attitudes and lifestyle. If patients can be educated and the procedures explained properly, it will go a long way to influence their expectations and help them become more receptive to a change in approach.	***PTm9****: It is possible they might also accept the treatment if it costs less but they need to be educated, in such a situation one has to spend more than that ten minutes to convince them.*<br><br>***PatM2****: You see, the patient attitude is determined by the amount of information that he has, so if you can clearly explain the approach and how it works you are likely to get a positive attitude from the patient.*
**Experiences**<br>This describes instances where attempts to install change have been made and the effects	Self-discovery	Patients relate situations when they needed to be convinced from within, even though their therapist tried by advising them they still needed to see for themselves to be successfully convinced to overcome the initial resistance. Based on their experiences, they relate that their outlook changed and they experience better outcomes.	***PatF7****: The clinician asked me the kind of bed I used… I told him it cannot be my bed, and all that but you know after the whole thing I was querying things, then I travelled for two weeks and I used another bed then I was able to conclude it was the bed.*<br><br>***PatF9****: I do exercise often but I stopped, I fear that what I am doing is even increasing the pain. I don't want to worsen the condition, and my husband told me to stop… but when I went to the clinic they did something similar and it subsided.*
**Strategies for implementation**<br>This highlights the participants' key ideas on strategies for the implementation of the stratified care approach.	Struggle against false information	Patients relate that there is a variety of information sources and many of which are misleading. These can come from other patients, from the internet or clinicians with certain motives. There is a constant struggle against wrong information by the patients which the therapists need to drive against to optimise care.	***PatM3****: Yes, they do mislead patients sometimes for financial implications, they may end up telling you to do something that is not necessary they can advise the patient to go for that treatment even though they know it might not help, so it requires a medical practitioner with integrity to be able to come out truthfully.*<br><br>***PatM2****: Yes, so be careful of the advice you get from clinicians because you might be getting it from quacks, make sure you get the advice from the right source.*

* Physiotherapists' and patient's quotes with pseudonymous designations comprising the numerical order of interviews.

For peer review only

Category 7: Recognising the need for change<br><br>Themes of variation	Category Description: This category is relevant for physiotherapists and patients. It describes participants attention to deficiencies and deterrents in physiotherapists knowledge and practice, further corroborated by the patient's dissatisfaction with the current treatment outcomes which can be enabled by acceptance of the stratified care approach and proper funding.		
	**Recognising the need for change**	**Explanation**	**Quotes***
**Tradition of treatment**<br>This describes components of the usual care, highlighting gaps in practice and aspects that can be modified to optimise care.	Lack of training to give psychologically informed therapy	Physiotherapists admitted having gaps in knowledge and lapses in training that might affect their ability to deliver optimal biopsychosocial care, this has resulted in sticking to the more familiar tradition of treatment. This issue of competency was a major setback. Physiotherapists felt incompetent to handle such an approach especially the prognostic approach and psychologically informed therapy.	***PTf4***: *Most therapists do know about the prognosis of LBP condition, they need to be educated better on prognosis because you can't classify patients using this approach if you do not know'*<br><br>***PTf8***: *Although general psychology was taught in school, this one relating to health care isn't taught. they do not know what exactly to do and how to do it. we just use the common-sense approach.*
**Evolution to a new system**<br>This discusses the key enablers needed to introduce a new practice aimed at optimising care.	Embracing a different approach	Physiotherapists and patients need to recognise the potential to optimise care to be open to accepting a different approach. The potential advantages need to be clear to participants creating an undeniable appeal of a different approach.	***PTm6***: *A model like this … I feel if we embrace it one way or the other it would make a difference.*<br><br>***PTf4***: *I think it will make life easier for patient and Physiotherapist if they can adopt it will go a long way to help,*
**Experiences**<br>This describes instances where attempts to install change have been made and the effects	No complete relief	Patients have undergone a series of treatments to change their condition but with no complete relieved some now seek solutions elsewhere. They express disappointment with their situation and frustration with the care they receive. They all feel that things need to change in the way physiotherapists treat patients.	***PatM4***: *Drugs, physiotherapy, I am not completely relieved, I just got tired of going to the hospital. because I have been going for physiotherapy for the past 2-3 years*<br><br>***PatM8***: *I thought by now I should have been fine…I feel the physiotherapy and medication wasn't doing anything to help my situation…I just felt I wasted my money and time,*
**Strategies for implementation**<br>This highlights the participants' key ideas on strategies for the implementation of the stratified care approach.	The role of funding	Participants complain of general issues relating to infrastructure, equipment and patient load. These issues are systemic constraints that are neither the fault of the patients nor the therapists. Participants jointly suggest improved funding to the health sector will improve services and aid implementation.	***PTm6***: *Facility and equipment are some of the main issues. No physiotherapist would be willing to use his funds to go print questionnaires for patients, most of our offices do not have computers, and printers available.*<br><br>***PatM8***: *Very poor, the infrared light they use on me everyone else has to wait until they are done with me, they have two and one is bad. So, if I needed it for ten mins it might be cut down to two or three minutes so that others can also use it.*

LBP: Low back pain

* Physiotherapists' and patient's quotes with pseudonymous designations comprising the numerical order of interviews.

For peer review only

Table 1 Consolidated criteria for reporting qualitative studies (COREQ): 32-item checklist

	No	Item	Guide questions/description
		**Domain 1: Research team and reflexivity**	
		Personal Characteristics	
Pg 5	1.	Interviewer/facilitator	Which author/s conducted the interview or focus group?
Pg 5	2.	Credentials	What were the researcher's credentials? *E.g. PhD, MD*
Pg 5	3.	Occupation	What was their occupation at the time of the study?
Pg 5	4.	Gender	Was the researcher male or female?
Pg 5	5.	Experience and training	What experience or training did the researcher have?
		Relationship with participants	
Pg 5	6.	Relationship established	Was a relationship established prior to study commencement?
Pg 5	7.	Participant knowledge of the interviewer	What did the participants know about the researcher? e.*g. personal goals, reasons for doing the research*
Pg 5	8.	Interviewer characteristics	What characteristics were reported about the interviewer/facilitator? e.g. *Bias, assumptions, reasons and interests in the research topic*
		**Domain 2: study design**	
		Theoretical framework	
Pg 4-5	9.	Methodological orientation and Theory	What methodological orientation was stated to underpin the study? *e.g. grounded theory, discourse analysis, ethnography, phenomenology, content analysis*
		Participant selection	
Pg 5	10.	Sampling	How were participants selected? *e.g. purposive, convenience, consecutive, snowball*
Pg 5	11.	Method of approach	How were participants approached? e.*g. face-to-face, telephone, mail, email*
Pg 8	12.	Sample size	How many participants were in the study?
Pg 8	13.	Non-participation	How many people refused to participate or dropped out? Reasons?
		Setting	
Pg 5	14.	Setting of data collection	Where was the data collected? e.*g. home, clinic, workplace*
Pg 5-6	15.	Presence of non-participants	Was anyone else present besides the participants and researchers?
Pg 8	16.	Description of sample	What are the important characteristics of the sample? *e.g. demographic data, date*
		Data collection	
Pg 6	17.	Interview guide	Were questions, prompts, guides provided by the authors? Was it pilot tested?
Pg 7	18.	Repeat interviews	Were repeat interviews carried out? If yes, how many?
Pg 5-6	19.	Audio/visual recording	Did the research use audio or visual recording to collect the data?
Pg 7	20.	Field notes	Were field notes made during and/or after the interview or focus group?
Pg 8	21.	Duration	What was the duration of the interviews or focus group?
Pg 5, 8	22.	Data saturation	Was data saturation discussed?
Pg 7	23.	Transcripts returned	Were transcripts returned to participants for comment and/or correction?
		**Domain 3: analysis and findings**z	
		Data analysis	
Pg 7	24.	Number of data coders	How many data coders coded the data?
Pg 7	25.	Description of the coding tree	Did authors provide a description of the coding tree?
Pg 7	26.	Derivation of themes	Were themes identified in advance or derived from the data?
Pg 7-8	27.	Software	What software, if applicable, was used to manage the data?
Pg 7	28.	Participant checking	Did participants provide feedback on the findings?
		Reporting	
Pg 10-17	29.	Quotations presented	Were participant quotations presented to illustrate the themes / findings? Was each quotation identified? e.*g. participant number*
Pg 17-19	30.	Data and findings consistent	Was there consistency between the data presented and the findings?
Pg 10	31.	Clarity of major themes	Were major themes clearly presented in the findings?
Pg 10	32.	Clarity of minor themes	Is there a description of diverse cases or discussion of minor themes?

(ii) Participant selection: Researchers should report how participants were selected. Usually purposive sampling is used which involves selecting participants who share particular characteristics and have the potential to provide rich, relevant and diverse data pertinent to the research question [13, 17]. Convenience sampling is less optimal because it may fail to capture important perspectives from difficult-to-reach people [16]. Rigorous attempts to recruit participants and reasons for non-participation should be stated to reduce the likelihood of making unsupported statements [18].

Downloaded from https://academic.oup.com/intqhc/article-abstract/19/6/349/1791966 by Fachhochschule Trier Hochschulbibliothek Standort Trier user on 22 August 2019

For peer review only
